# Long-Term Safety of Anti-Interleukin-1 Medications in Children with Rheumatic Diseases: a Systematic Review

**DOI:** 10.1007/s40272-025-00712-7

**Published:** 2025-08-20

**Authors:** M. Isa, G. M. Tiller, D. F. L. Liew, W. D. Renton

**Affiliations:** 1 Department of Rheumatology, Northern Health, Melbourne, Australia; 2 Rheumatology Team, Department of General Medicine, The Royal Children’s Hospital, Melbourne, Australia; 3 Department of Paediatric Rheumatology, Monash Children’s Hospital, Melbourne, Australia; 4 Department of Rheumatology, Austin Health, Melbourne, Australia; 5 Department of Clinical Pharmacology and Therapeutics, Austin Health, Melbourne, Australia; 6 Department of Medicine, University of Melbourne, Melbourne, Australia; 7 Faculty of Medicine Nursing and Health Sciences, Monash University, Melbourne, Australia

## Abstract

**Background:**

Anti-interleukin-1 (IL-1) biologic disease-modifying anti-rheumatic drugs are the mainstay for several childhood rheumatic and autoinflammatory diseases. Long-term medication safety is a key consideration for chronic disease management.

**Aim:**

The objective was to synthesise evidence on the long-term safety of anti-IL-1 medications in children and young people with rheumatic diseases, including autoinflammatory diseases.

**Methods:**

The study protocol was registered prospectively (PROSPERO CRD420251000272). Original full text studies of at least ten patients presenting safety data on anti-IL-1 medications in children with rheumatic diseases were eligible for inclusion. Medline, Embase and Web of Science were searched from inception to 27 February 2025. The methodological index for non-randomized studies (MINORS) tool was used to assess risk of bias. All relevant safety outcomes were presented and synthesised. Meta-analysis was not performed owing to study heterogeneity.

**Results:**

A total of 1660 unique records were screened, and 57 unique studies (3690 patients) were included. In total, 31 were retrospective cohort studies, and 10 were prospective interventional trials. Most studies were of moderate (*n* = 31) or high (*n* = 25) risk of bias. Rates of adverse events varied significantly between studies. Injection site reactions (particularly with anakinra) and minor infections were common. Infections were the most common type of serious adverse event. Drug reaction with eosinophilia and systemic symptoms (*n* = 3) and interstitial lung disease (including related conditions) (*n* = 9) were reported in patients with systemic onset juvenile arthritis only. Deaths (*n* = 16) and malignancies (*n* = 7) were uncommon, often occurring long after anti-IL-1 medication discontinuation and were often deemed to be unrelated to the anti-IL-1 medication.

**Conclusions:**

Our results are consistent with the known safety profile of anti-IL-1 medications and show that they are generally safe for use in the context of childhood rheumatic and autoinflammatory diseases. This review of clinical trial and real-world data will help inform clinical decision-making and family counselling when initiating anti-IL-1 medications in children.

**Supplementary Information:**

The online version contains supplementary material available at 10.1007/s40272-025-00712-7.

## Key Points

**Table:** 

This study looked at safety information from 57 studies involving nearly 3700 children and young people who used anti-IL-1 medications for rheumatic or autoinflammatory conditions.
Common side-effects were generally mild and included reactions at the injection site and infections such as viral upper respiratory tract infections.
Serious side-effects including deaths and cancers were very uncommon and often happened long after treatment had stopped, suggesting that anti-IL-1 medications are generally safe for children with rheumatic and autoinflammatory conditions.

## Introduction

The introduction of biologic disease-modifying anti-rheumatic drugs (bDMARDs) for the management of paediatric rheumatic diseases has significantly improved the outlook for many children and young people with these conditions. bDMARDs interrupting the interleukin-1 (IL-1) pathway have been particularly revolutionary for a select group of childhood rheumatic diseases, including several systemic autoinflammatory diseases (SAIDs) [1].

The interleukin-1 (IL-1) family of ligands and receptors are crucial mediators of innate immunity and inflammation. Dysfunctional IL-1 signalling is directly implicated in the pathogenesis of monogenic inflammasomopathies, including familial Mediterranean fever (FMF), cryopyrin associated periodic syndromes (CAPS), mevalonic kinase deficiency (MKD) and tumor necrosis factor receptor-associated periodic syndrome (TRAPS) [2]. IL-1 is also a key mediator in other complex polygenic autoinflammatory syndromes such as systemic onset juvenile arthritis (SJIA)/Still’s disease [3]. These conditions have a marked effect on quality of life and can be associated with high risk of mortality if not effectively treated [2]. bDMARDs targeting the IL-1 pathway are highly effective and now widely used for conditions including Still’s disease [3, 4] and many monogenic SAIDs [5].

These medications may be used for many years or even indefinitely; hence, long-term medication safety is a key consideration when managing these patients. IL-1 plays an important role in host defence against infection, and susceptibility to infection is a potential risk with IL-1-inhibiting medications. Other severe adverse effects such as drug reactions and increased risk of malignancy are also important to consider with any immunomodulatory treatment. Additionally, the widespread use of anti-IL-1 treatments has coincided temporally with an increased incidence of pulmonary disease in patients with SJIA [6]. The relationship between SJIA-associated lung disease and bDMARD treatment remains unclear, and it is not known if this syndrome occurs in other clinical contexts.

We conducted a systematic literature search to synthesise the available evidence on the long-term safety of anti-IL-1 medications in children and young people with rheumatic diseases to provide best available evidence for clinicians and their patients.

## Methods

We performed a systematic review on the long-term safety of anti-interleukin-1 medications in children and young people with rheumatic diseases. A population, intervention, comparator and outcome (PICO) framework was used to define the search. The review was conducted according to the Preferred Reporting Items for Systematic Reviews and Meta-Analyses (PRISMA) 2020 statement [7]. The study protocol was registered prior to data extraction (PROSPERO registry number CRD420251000272).

### Eligibility

Children and young people with rheumatic diseases, including autoinflammatory diseases, were eligible for inclusion. Studies in which the majority of patients were over the age of 18 years at the time of commencing treatment were excluded, unless data specific to the included children and young people were presented.

Interventions were limited to anakinra, canakinumab and rilonacept. Studies required a minimum of 12 months (mean or median) of anti-IL-1 medication exposure to be included. For studies including multiple interventions (e.g. biologic registries reporting on anti-IL-1 bDMARDs and other bDMARDs), data were only included for analysis if disaggregated data relating specifically to anti-IL-1 bDMARDs were presented.

We anticipated that most included studies would be observational in nature; however, all original studies, including randomised control trials (RCTs), were eligible for inclusion. Review articles including systematic reviews were excluded.

Only full text original articles in English were included. We excluded reports with fewer than ten patients. We had no restriction on the date of publication.

### Search Strategy and Selection Process

We performed a search of Medline, Embase and Web of Science (Clarivate) from inception to 27 February 2025. Broad inclusion criteria were used to identify all studies reporting long-term safety data on anti-IL-1 bDMARD treatment in children and young people with rheumatic diseases (Medline search strategy included in Supplementary Information). Records were imported into Endnote™ (Clarivate, PA, USA) and de-duplicated. Results were then imported into Covidence (Veritas Health Innovation, Melbourne, Australia) and again de-duplicated. Two authors (M.I. and W.R.) independently screened titles and abstracts. Two authors (M.I. and W.R.) independently assessed full texts for inclusion in the review. Conflicts were discussed and resolved with input from a third author (G.T.) as needed. No automation was used to screen titles, abstracts or full texts.

To address multiple reporting, included reports were assessed for similarities in trial registration numbers, authors, locations, medications and participant numbers. If reports were deemed likely to overlap with each other, then this was noted.

### Data Collection Process

Data including study details, cohort characteristics and safety outcomes were extracted by single reviewers (G.T., M.I. and W.R.) into a template and checked by at least one other author (M.I. and W.R.) (included study characteristics table in Supplementary Information).

### Data Items

Data items included study name, study identifiers, date of study, study design, condition(s), medication(s), number of patients, age of patients, follow up duration, country, sex, author’s conclusions and adverse effects.

Adverse effects included infections, malignancies, interstitial lung disease, drug reactions (including injection site reactions, anaphylaxis, Stevens–Johnson syndrome, toxic epidermal necrolysis and drug reaction with eosinophilia and systemic symptoms (DRESS)), severe adverse effects (as defined by authors), treatment discontinuations due to adverse effects and deaths.

Where studies reported on multiple anti-IL-1 medications, the total number of anti-IL-1-medication-exposed patients was reported (patients counted once if they were exposed to two anti-IL-1 medications). If a report described the number of patients exposed to individual anti-IL-1 medications without adequately describing how many were exposed to several, then these were assumed to be exclusive groups.

### Study Risk of Bias Assessment

The methodological index for non-randomized studies (MINORS) tool was used to assess risk of bias [8]. A single author reviewed each included study (M.I., G.T. and W.R.) and each was checked by at least one other author (M.I. and W.R.). Risk of bias assessments were documented alongside the study details and results (included studies table in Supplementary Information)

### Synthesis Methods

It was anticipated that a high degree of heterogeneity would be encountered with regard to diagnoses, treatments and outcome measures. Data synthesis was largely descriptive, and studies at lower risk of bias were prioritised. Meta-analysis was not performed given anticipated heterogeneity and high risk of bias. Events per patient-year (PY) of exposure was the preferred effect measure; however, other descriptors were accepted for descriptive analysis.

### Reporting Bias and Certainty Assessments

Reporting bias and certainty assessments were not performed.

## Results

### Study Selection

**Fig. 1:**
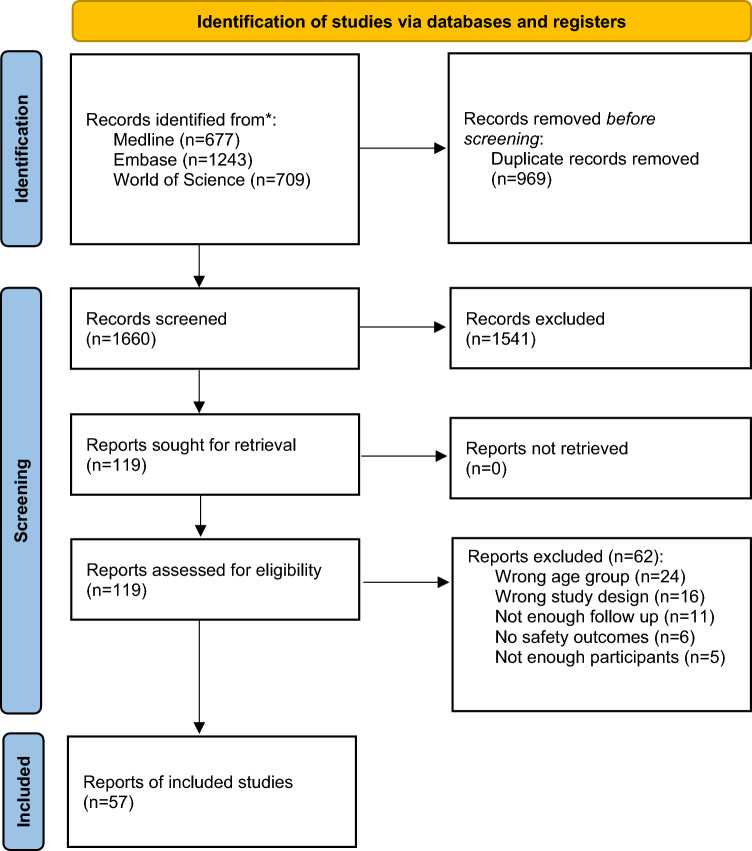
PRISMA flow diagram showing study selection process [7]

A total of 2629 records were identified on the initial search (1660 unique records after de-duplication); 119 full texts were sought for retrieval. All 119 were retrieved and assessed for eligibility. In total, 57 studies were included in the review. Figure 1 details the study selection process using the PRISMA 2020 flow diagram format [7]. Reasons for full text exclusion are documented in the excluded study table in the Supplementary Information.

### Study Characteristics

In total, 57 studies were included in the review, including a total of 3690 anti-IL-1-medication-exposed patients (excluding one study that did not report the total number of included patients [9]). Total duration of medication exposure has not been presented, as many studies did not include mean or total time on treatment. Extracted data and risk of bias assessments are detailed in the included study characteristics tables in the Supplementary Information.

Among the included studies were retrospective cohort studies (*n* = 31), registry based observational studies (*n* = 10), open label prospective studies (*n* = 5), randomised controlled trial (RCT) studies (*n* = 4), RCT long-term extension (LTE) studies (*n* = 4), prospective cohort studies (*n* = 2) and a post-marketing pharmacovigilance reporting system study (*n* = 1).

The smallest included studies were retrospective cohort studies of ten patients [10–12], while the largest was a retrospective cohort study including 335 anti-IL-1-medication-exposed patients [13]. There was also a large Food and Drug Administration Adverse Event Reporting System (FAERS) post-marketing pharmacovigilance study that reported 28,496 canakinumab-related adverse events but did not report the total number of included patients [9]. The median number of included patients per study was 43.

In total, 34 (59.6%) of the included studies had a female predominance, and 18 (31.6%) had a male predominance. The earliest report was published in 2009 [14], while the most recent was in 2025 [9]. Studies were based in Türkiye (*n* = 16), the USA (*n* = 5), France (*n* = 4), Germany (*n* = 4), the UK (*n* = 3), Israel (*n* = 2), Italy (*n* = 2), Japan (*n* = 2), China (*n* = 1), Czech Republic (*n* = 1), Netherlands (*n* = 1), Russia (*n* = 1) and Saudi Arabia (*n* = 1). A total of 14 were performed across multiple countries.

Disease cohorts included juvenile idiopathic arthritis (including SJIA) (24 studies, 2001 patients) and defined SAIDs including familial mediterranean fever (FMF) (11 studies, 338 patients), cryopyrin-associated periodic syndromes (CAPS) (7 studies, 135 patients) and mevalonate kinase deficiency (MKD) (1 study, 74 patients). In total, 14 studies included multiple conditions and/or undifferentiated SAIDs (14 studies, 1142 patients).

A total of 15 studies (1009 patients) reported on anakinra, 23 (1485 patients) on canakinumab, 2 (94 patients) on rilonacept and 17 (1102 patients) reported on multiple anti-IL-1 medications (Table 1).

**Table 1:** Included studies by medication and indication

Condition	Medication			
	Anakinra	Canakinumab	Rilonacept	Multiple anti-IL-1
JIA	*Atemnkeng Ntam 2021* (*n* = 51)^a^ [20] *Brunner 2020* (*n* = 123)^a^ [15] *Foley 2023* (*n* = 21) [49] *Giancane 2022* (*n* = 306) [43] *Ilowite 2009* (*n* = 86) [52] *Lequerre 2008* (*n* = 35) [14] ***Nigrovic 2011*** (*n* = 46) [53] ***Sota 2019*** (*n* = 137)^a^ [29]	*Iwata 2023* (*n* = 19) [33] *Quartier 2021* (*n* = 182) [44] *Ruperto 2018* (*n* = 177)^a^ [16] *Ruperto 2012* (*n* = 23) [34]	**Ilowite 2014** (*n* = 71) [30] *Lovell 2013* (*n* = 23) [45]	***Balci 2020*** (*n* = 15)^b^ [41] *Dumaine 2020* (*n* = 104)^a,^^b^ [18] *Horneff 2017* (*n* = 60)^a,^^b^ [21] ***Kip 2023*** (*n* = 25) [65] *Klein 2020* (*n* = 122)^b^ [22] ***Lainka 2021*** (*n* = 111) [50] ***Sota 2018*** (*n* = 77)^a^ [28] *Tanatar 2023* (*n* = 12) [36] *Thiele 2021* (*n* = 105) [23] *Woerner 2015* (*n* = 70) [32]
FMF		***Berdeli 2019*** (*n* = 22) [66] *Gulez 2020* (*n* = 15) [40] ***Kisla Ekinci 2019*** (*n* = 14) [55] **Ozen 2020** (*n* = 60) [25] *Shehadeh 2024* (*n* = 51) [67] ***Yazilitas 2018*** (*n* = 11) [37] ***Yucel 2021*** (*n* = 65) [56]		*Kurt 2020* (*n* = 25) [57] ***Özçakar 2016*** (*n* = 13) [68] ***Pinchevski-Kadir 2023*** (*n* = 22) [69] ***Sag 2020*** (*n* = 40) [51]
CAPS	*Kullenberg 2016* (*n* = 43)^a^ [26] ***Neven 2010*** (*n* = 10) [10] *Sibley 2012* (*n* = 26)^a^ [27]	*Brogan 2019* (*n* = 17) [58] ***Russo 2014*** (*n* = 10) [11] *Yokota 2017* (*n* = 19) [35] ***Zhu 2024 A*** (*n* = 10) [12]		
MKD		*Jeyaratnam 2022* (*n* = 74) [24]		
Multiple or other	***Al-Mayouf 2016*** (*n* = 23) [38] ***Demir 2022*** (*n* = 33) [60] *Fingerhutova 2022* (*n* = 47) [46] *Garg 2019* (*n* = 22) [59]	*Adıgüzel Dundar 2024* (*n* = 67) [39] ***Alexeeva 2023*** (*n* = 32) [70] ***Cakan 2020*** (*n* = 29) [54] ***Coskuner 2023*** (*n* = 335) [13] *Kilic Konte 2024* (*n* = 189) [42] *Kone-Paut 2024* (*n* = 64) [19] ***Zhang 2025 (n = not reported)*** [9]		*Cabrera 2019* (*n* = 160) ^a,^^b^ [17] ***Jones 2024*** (*n* = 65) [71] ***Nur Sunar Yayla 2024*** (*n* = 76)^b^ [72]

*n* = number of patients treated with individual anti-IL-1 medications

Bold indicates **low risk of bias**, italics indicates *moderate risk of bias*, bold italics indicates ***high risk of bias***

*CAPS* cryopyrin associated periodic syndromes, *FMF* familial mediterranean fever, *JIA* juvenile idiopathic arthritis, MKD mevalonate kinase deficiency

^a^Study with cohort overlapping another study

^b^At risk of double-counting patients, as may have been exposed to two anti-IL-1 medications

Several studies were assessed as likely overlapping with regard to included patients. In total, two studies reported on the long-term effect of canakinumab in patients with SJIA from several trials (NCT00891046, NCT00889863 and NCT00886769) [15, 16]. In total, three studies reported on the multinational JIRcohorte registry [17–19], and four studies reported on the German BIKER registry [20–23]. A total of two studies reported results from the CLUSTER trial; one limited to canakinumab-treated patients with MKD [24] and the other to patients with colchicine-resistant FMF [25]. In total, two studies reported on results from a prospective open label study on anakinra in patients with severe CAPS (NCT00069329) [26, 27]; two studies described outcomes in anakinra-treated patients with SJIA in Italy and shared the same ethics committee reference number (364-16OCT2013) [28, 29].

### Risk of Bias in Studies

Two prospective interventional studies were assessed as being of low risk of bias [25, 30]. In total, 31 studies were assessed as being of moderate risk of bias, while 24 were high risk of bias.

Almost all studies clearly stated their aims; however, other items in the MINORS instrument were frequently absent, not reported or inadequate. Many studies inferred that consecutive patients were included; however, this was usually inadequately described. Almost half of the studies included at least some prospectively collected data. The specific focus of the included studies was very varied, and hence, the endpoints were quite heterogeneous. Most studies included important endpoint measures such as infections and severe adverse events; however, others had very minimal safety data. Blinded evaluation of study endpoints was very uncommon in the included studies. Over half of the included studies had a follow-up period of less than 2 years; while this was suitable for some study endpoints, it was deemed insufficient for some important outcomes. Few studies specifically commented on patients lost to follow-up, and no study prospectively calculated required study size to detect relevant safety outcomes.

### Safety Results of Individual Studies and Results of Syntheses

#### Infections

Infections were the most commonly reported adverse event, and all but eight of the included studies specifically presented data on infections. Methods of reporting and categorising infection were very varied. Rates of infection, overall and for specific infectious syndromes, also varied considerably between studies. Similar to that seen with other bDMARDs in children, most infections were mild, and severe infections were often observed in patients with prior or concomitant immunosuppressive therapy. Notably, no substantive signal has emerged for opportunistic infections that was not identified in the original anakinra registration studies [31].

Upper respiratory tract infections, including nasopharyngitis, rhinitis, tonsillitis and sinusitis, were the most commonly reported type of infection. Other frequently reported infections included gastroenteritis, urinary tract infections, lower respiratory tract infections, cellulitis and lymphadenitis.

Viral infections were common overall but often undifferentiated. Influenza was the most commonly reported viral pathogen. Cases of other common childhood viral infections including varicella [9, 15, 16, 23, 32], Epstein–Barr virus [9, 13, 17, 33–35] and cytomegalovirus [15, 32, 36] were also reported.

Streptococcal infection (mainly pharyngitis) was reported by several studies [9, 16, 26, 27, 30]. Staphylococcal infection was reported infrequently but included one case of peritonitis [37] and sepsis [15]. One study reported two deaths from Gram-negative sepsis [38]. Few fungal skin infections and a single case of fungal peritonitis were reported [37].

Several studies looked at the risk of tuberculosis infection in endemic areas. Two Turkish studies reported high rates of latent tuberculosis infection prior to and during treatment with canakinumab for a range of autoinflammatory conditions; however, no episodes of active *Mycobacterium* tuberculosis infection were observed [39, 40]. Another Turkish study reported two lung tuberculosis infections in children on canakinumab for SJIA [41].

A minority of studies reported an absence of any observed infections, including a relatively large retrospective cohort study of children with SJIA (*n* = 61) on anakinra treatment [29].

#### Malignancies

Malignancy outcomes were specifically reported in 12 studies, of which, seven reported no cases of malignancy in their cohorts. A total of seven events were recorded, although two reports of a patient with acute myeloid leukaemia (AML) may overlap [20, 22]. Observed malignancies often occurred after anti-IL-1 therapy discontinuation and were often deemed to be unrelated to the anti-IL-1 medication. Notably, follow-up was variable and therefore may not consistently capture all long-term risk, although registry and long-term extension data demonstrate no applicable signals.

Haematological malignancies were the most common; three cases of AML were reported [13, 20, 22]. One patient receiving canakinumab for FMF was diagnosed with AML, and canakinumab treatment was discontinued [13]. Another patient was a female who had received anakinra at the age of 9 months for SJIA. She had developed severe macrophage activation syndrome (MAS) during her anakinra treatment, which was treated with steroids, cyclosporin A and etoposide. She developed AML at the age of 4 years and reached remission after allogeneic stem cell transplantation from a family donor [22]. A third patient was diagnosed with myeloid leukaemia 3 years after the last anakinra dose for SJIA. This patient had developed MAS during their anakinra therapy, leading to cessation of anti IL-1 therapy. As a result, the leukaemia was considered to be unrelated to the anakinra treatment [20]. The second and third patients were both from the BIKER registry with several overlapping clinical details suggesting the two reports may refer to the same individual [20, 22].

In total, two cases of lymphoma and one case of an ovarian mass were detected in a large Turkish study looking at the use of canakinumab for a range of paediatric rheumatic diseases [42].

One additional case of anaplastic large cell lymphoma was diagnosed after 113 days of canakinumab treatment for SJIA. The authors determined that the initial articular symptoms and fever were likely paraneoplastic manifestations resulting in an incorrect diagnosis of SJIA. This diagnosis was therefore considered unlikely to be related to canakinumab [15].

#### Interstitial Lung Disease (ILD)

A total of eight studies reported on significant non-infectious respiratory pathology such as interstitial lung disease (ILD) in anti-IL-1-treated patients. All of these events were reported in patients with SJIA, except for one patient with an unclear diagnosis in a cohort consisting of multiple childhood rheumatic diseases (including SJIA) [42].

Overall, three were cases of interstitial lung disease [42–44]. Two of these cases were associated with canakinumab [42, 44], while the other patient had received anakinra [43]. One of these patients on canakinumab for an unknown indication developed ILD and drug reaction with eosinophilia and systemic symptoms (DRESS) syndrome [42]. The other patient on canakinumab for SJIA was reported to have only mild ILD [44]. One patient reportedly had ILD after receiving treatment with anakinra for SJIA for over 24 months [43].

There were another four patients with potentially similar pathology. One patient had MAS and pulmonary fibrosis after treatment with rilonacept for SJIA; however, this was assessed as not related to the drug by treating physicians [45]. There was one case of prolonged eosinophilia with anakinra therapy for SJIA with “associated lung involvement” [46]. In a study on canakinumab for SJIA, there was one death related to lung injury in a patient with MAS events complicated by pulmonary hypertension and interstitial pneumonia [16]. Another patient in the same study had developed acute interstitial pneumonitis and transfusion-related lung injury, which resolved spontaneously but led to the patient discontinuing the study [16].

There were two cases of pulmonary alveolar proteinosis, a high-risk lung condition which more recently has been observed to occur in patients with SJIA with severe disease [47, 48]. One severe case developed in the context of canakinumab therapy in a patient with SJIA that led to study discontinuation for that individual [44]. A second patient was a 17-month-old female with treatment-refractory SJIA and smouldering MAS who had received treatment with both anti-IL6 and anti-IL1 blockade, in addition to cyclosporine, before progressing to etoposide as a bridge to allogeneic haematopoietic stem cell transplantation [49].

Another patient with SJIA in the same study had patchy ground glass changes bi-basally at diagnosis and achieved remission and remained off all treatment within 2.7 years after being commenced on anakinra within 3 months of diagnosis [49].

#### Drug Reactions

Injection site reactions were one of the most frequently reported adverse event. Almost all of the studies examining anakinra reported injection site reactions, while only a minority of the canakinumab studies did. Of the studies that included patients using anakinra and canakinumab, rates of injection site reaction were generally higher with anakinra use, and several patients were switched from anakinra to canakinumab for this reason [36, 50, 51].

One randomised controlled trial of anakinra for SJIA reported drug application site reactions in 64 of 86 (74%) patients [52]. This included pain in 29 (33%), pruritus in 26 (30%), rash in 14 (16%), ecchymosis in 12 (14%), reaction in 10 (12%), oedema in 9 (11%) and inflammation in 8 (9%). One retrospective cohort study reported injection site reactions in 20 of 45 (44%) patients [53], while several others observed rates closer to 25% [26, 36, 46, 51]. Several studies made note that anakinra-associated injection site reactions tended to improve over time [14, 46, 52].

Of the two RCTs that included patients on rilonacept, one reported injection site reactions in one third of patients during the double-blind phase. Injection-site erythema was only noted in rilonacept-treated patients, while injection-site bruising was reported both in patients receiving placebo and in patients receiving rilonacept [45]. Canakinumab was generally well tolerated [11, 34, 40, 54, 55] with few reported injection site reactions [12, 56].

Severe drug reactions were uncommon. Only one registry-based study on SJIA reported a case of anaphylaxis in 1 of 51 (2.0%) canakinumab-treated patients. Another study on juvenile arthritis reported hypersensitivity in 2 of 60 (3.3%) anti-IL-1-medications-treated patients (drug not specified, cohort included anakinra and canakinumab) but did not provide further detail on the nature of these events [21]. Additionally, there were single reports of angioedema [27] and allergic reaction with severe disseminated rash [57] with anakinra treatment.

Two studies reported a total of three cases of DRESS syndrome. One RCT LTE study on canakinumab for SJIA (*n* = 177) reported two cases of DRESS syndrome (1.1% of patients, event rate 0.42/100 PY). One retrospective cohort study reported a case of DRESS and interstitial lung disease among 189 canakinumab-treated patients. This cohort included a range of diseases including SJIA and several monogenic autoinflammatory diseases [42].

#### Severe Adverse Effects

In total, 39 studies reported on severe or serious adverse events (SAE), although there was wide variation with regard to the definition of SAE. A total of eight of these studies reported no SAEs in their cohorts; nine studies specified their definition of SAE. Medical Dictionary for Regulatory Activities (MedDRA) classification was utilised in three studies [17, 43, 58], and one study used Common Terminology Criteria for Adverse Events (CTCAE) to indicate the severity of severe infectious adverse events [18].

The most commonly reported SAE was infection, with a total of 131 cases of serious infection reported. Viral infections were most commonly reported (*n* = 19) [14, 16, 30, 32, 34, 35, 46, 58, 59], with seven cases of varicella zoster virus [16, 30, 32, 59]. Other serious viral infections included two cases of EBV [34, 35], two cases of cytomegalovirus [32], two cases of viral gastroenteritis [16], one case of influenza [58] and one case of severe acute respiratory syndrome coronavirus 1 (SARS-CoV-2) coronavirus infection [46]. There were a total of eight confirmed bacterial serious infections including streptococcal [16, 30], salmonella [32] and *Borrelia burgdorferi* [32] infections. One case of visceral *Leishmania* infection was reported [14].

Additionally, there were 31 respiratory infections and 10 cases of skin and soft tissue infections where the causative organism was not specified, and the infection was assessed as a serious adverse event. The most common severe respiratory infection was pneumonia (*n* = 28) [16, 26, 29, 32, 35, 42, 43, 58]. There were a range of skin and soft tissue infections including wound infection (*n* = 5) [26, 27, 58], cellulitis (*n* = 2) [26, 59], subcutaneous abscess (*n* = 2) [16] and lower limb ulcers (*n* = 1) [29].

There were 42 cases of MAS among patients using anakinra (*n* = 16) [20, 26, 27, 29, 32, 43] and canakinumab (*n* = 26) [13, 16, 32, 42]. There were no reported cases of MAS with rilonacept; however, there was one case of haemophagocytic lymphohistiocytosis (HLH) [30]. Other haematological manifestations included non-specific blood and lymphatic disorders (*n* = 11) [33, 44], neutropenia requiring hospital presentation (*n* = 3) [59], lymphadenopathy (2 cases) [16] and AML (*n* = 1) [13].

There were nine cases of serious injection related reactions related to anakinra [43, 59]. There were 43 cases of flare of inflammatory condition [24, 25, 42, 45].

Although the majority of the studies did not comment on the incidence rate of SAEs, two studies reported declining rates of SAE with time [15, 26].

#### Treatment Discontinuations Due to Adverse Effects

Half of the included studies reported on treatment discontinuations due to adverse effects. In total, nine studies (two anakinra and seven canakinumab) stated that they had none [12, 24, 26, 27, 33–35, 54, 58].

A few studies reported rates of treatment discontinuation due to adverse effects of over 10%; however, most were significantly lower. A multinational LTE study on patients treated with canakinumab for SJIA noted that 14 of 123 (11.4%) patients discontinued the medication owing to events including MAS, EBV infection and anaplastic large cell lymphoma.

Injection site reactions, intolerance and trypanophobia were common reasons for discontinuing anakinra [14, 51–53, 59, 60]. A multicentre RCT and LTE study of canakinumab for SJIA reported that 19 of 177 (10.7%) patients discontinued treatment owing to intolerance but did not specify the nature of intolerance [16]. Injection site reactions were otherwise quite uncommon in canakinumab- and rilonacept-treated patients.

Several studies reported discontinuation due to hepatic issues including eosinophilic hepatitis (anakinra) [53], moderate-to-severe hepatic failure (anakinra) [36], hepatic failure (canakinumab) [13] and raised liver transaminase levels (rilonacept) [30].

Other reasons for treatment discontinuation that involved multiple patients included development of Crohn-like disease (canakinumab) [13], headache (anakinra) [57], MAS (canakinumab, rilonacept) [42, 44, 45] and disease flares (canakinumab) [42, 44].

#### Death

In total, eight studies reported deaths among their patient cohorts. A total of 16 patients died, of whom, only 5 were on anti-IL-1 medication at the time of death.

A multinational RCT LTE study reported three deaths among 177 patients with SJIA treated with canakinumab [16]. One death occurred during the open-label treatment phase due to MAS and severe pulmonary hypertension while on treatment with canakinumab. Another patient died due to MAS 2 days after discontinuing the randomised withdrawal phase due to MAS and urosepsis (over 164 days after her last dose of canakinumab). Another patient died from disease progression 3 months after discontinuing the long-term extension phase due to unsatisfactory therapeutic effect (not on canakinumab). Additionally, a fourth death was reported in the reference RCT publication; a 19-year-old woman died more than 2 years after receiving the last dose of canakinumab from streptococcus pneumoniae sepsis [61].

A multinational prospective open-label study evaluating canakinumab in SJIA (*n* = 23) reported that no deaths were reported during the study period; however, a 22-year-old female patient died of pneumococcal sepsis 2.3 years after the last canakinumab injection, having only received two doses during the study [34].

A multinational registry noted three deaths among 306 anakinra-treated patients with SJIA; however, none were on anakinra at the time of death (death occurred 0.5, 3 and 5 years after discontinuation) [43].

A Saudi Arabian-based retrospective cohort study exploring a range of biologic therapies for many conditions reported two deaths among 134 biologic-treated children (23 of whom were on anakinra) [38]. Both patients died from septic shock with Gram-negative sepsis while taking anakinra.

A Czech retrospective cohort study including multiple paediatric onset diseases (*n* = 47) reported one death of an adult patient [46]. They were described as a polymorbid patient with Muckle–Wells syndrome, and they died at 56 years of age after 2 months of anakinra treatment from a relapse of pre-existing pancreatitis and acute pulmonary embolism.

A UK-based retrospective cohort study of anakinra-treated patients with undifferentiated autoinflammatory disease reported three deaths of 22 (13.6%) patients [59]. One patient died owing to MAS while on treatment with anakinra; two patients died after discontinuing anakinra, one due to multiorgan failure from chronic atypical neutrophilic dermatosis with lipodystrophy and elevated temperature (CANDLE) syndrome and one from multi-organ failure from periodic fever, immunodeficiency and thrombocytopenia (PFIT) syndrome.

A Turkish retrospective cohort study of canakinumab for FMF reported one death among 11 patients. The death occurred 1 year after canakinumab cessation due to sepsis related with mixed fungal and staphylococcal peritonitis [37].

In addition to the cases above, a large post-marketing pharmacovigilance study of children and adults treated with canakinumab noted 1604 deaths reported to the US Food and Drug Administration Adverse Event Reporting System [9]. This accounted for 5.6% of all extracted adverse events. Cause of death was reported in some cases with the most common causes being pneumonia (*n* = 45), coronavirus disease 2019 (COVID-19) (*n* = 24), condition aggravation (*n* = 12) and infection (*n* = 12).

## Discussion

To our knowledge, this is the first systematic review synthesising data on the long-term safety of anti-IL medications in children and young people with rheumatic diseases. It includes data on commonly used anti-IL-1 medications for a range of different indications including SJIA, FMF and CAPS. Data have been derived from a range of clinical trials and real world studies providing results that should be generalisable to the population of interest. Across the included studies, anti IL-1 medications were generally well tolerated, with no new safety signals identified beyond those already known to occur in children and adult populations and an adverse event profile similar to other bDMARDs in children and young people.

Rates of safety events varied considerably between studies, and this may be due to varying interventions and populations. The most common adverse events were generally mild in nature. Injection site reactions were very common, particularly with anakinra. Common childhood infections including upper respiratory tract infections were also frequently observed. Two included RCTs that presented data from the double blind phase showed no major difference in the rates of common childhood illnesses between anti-IL-1- and placebo-treated patients; however, numbers were limited [30, 52].

Serious adverse events were relatively uncommon in most studies. Few deaths were observed, and most were not seemingly related to the anti-IL-1 medication. Of note, many of the deceased patients had discontinued anti-IL medication many months or years before their death. Severe lung pathology including interstitial lung disease was observed in several patients with SJIA but not otherwise. Rates of malignancy were very low and in many instances were deemed to be unrelated to the medication of interest. A range of severe liver pathology including hepatic failure was observed and led to treatment discontinuation in several individuals.

Discontinuations due to adverse events, a helpful proxy for medication tolerability, was generally low, at less than 10% in most studies and at 0% in many. Injection site reaction was the most common adverse event leading to discontinuation.

Injection site reactions were more commonly reported in studies on anakinra compared with canakinumab. This is in keeping with large adult trials that show high rates of injection site reactions, thought to be mainly due to vehicle constituents [62]. Anakinra is also dosed more frequently compared with the other anti-IL-1 medications, and this is likely to add to the overall burden of treatment. Analysis of quality of life measures has previously suggested that canakinumab has a favourable impact on psychosocial concepts when compared with anakinra [63]. There were no other clear signals of differing safety profiles between different anti-IL medications.

Differences between the study populations may also explain some of the discrepant safety results. Direct comparisons between populations were difficult; however, overall there were no clear signals to suggest a different safety profile. The one exception was DRESS and ILD that were only reported in patients with SJIA (noting that one study that included patients with SJIA and AID reported a single patient with DRESS and ILD but did not specify that patient’s underlying disease). These are conditions that are known to be associated with SJIA, and the impact of bDMARDs, including anti-IL medications, is unclear [64]. One study compared canakinumab safety outcomes in patients with SJIA (*n* = 55) and other SAID (*n* = 280) with no clear differences noted [13].

This study is not without limitations. First, the majority of included studies were observational cohort studies, limiting the ability to draw strong causal inferences regarding adverse events. Second, long-term safety data remain limited, with most included studies having a mean follow-up duration of less than 4 years. While this timeframe should be adequate to assess many outcomes, it may be insufficient to accurately assess important outcomes such as risk of malignancy which may not eventuate for many years or even decades. Third, there was considerable heterogeneity in safety reporting across included studies, and few used standardized adverse event classification systems, making direct comparisons challenging.

## Conclusions

We identified a large set of data reporting on long-term safety outcomes in children and young people with rheumatic diseases undergoing treatment with anti-IL-1 medications. The identified adverse event profile was similar to other bDMARDs in children and young people. Severe adverse effects were relatively uncommon; however, clinicians should maintain regular surveillance for infections, disease specific complications (such as DRESS and ILD in SJIA) and injection site reactions, particularly early in the course of treatment. While most included studies were of moderate-to-high risk of bias, the findings from this review can help inform clinical decision-making and family counselling when initiating anti-IL-1 medications in paediatric populations. Further high-quality studies with longer periods of follow-up are required to truly understand the risk profile of these medications.

## Supplementary Information

Below is the link to the electronic supplementary material.

Supplementary file1 (PDF 940 KB)

## Declarations

### Data and Materials Availability

All data generated or analysed during this study are included in this published article and its supplementary information file. Additional details are available from the corresponding author on reasonable request.
