# Supplementary material for: Long-Term Safety of Anti-Interleukin-1 Medications in Children with Rheumatic Diseases: a Systematic Review
Source: Paediatr Drugs. 2025 Aug 20;27(6):693–705. doi: 10.1007/s40272-025-00712-7 (PMC12534318; doi:10.1007/s40272-025-00712-7)
Supplement: Supplementary file 1 — Supplementary file1 (PDF 940 KB) [file 40272_2025_712_MOESM1_ESM.pdf]

Supplementary Information

Long-term Safety of Anti-Interleukin-1 Medications in Children with Rheumatic Diseases: A Systematic Review

Isa M, Tiller GM, Liew DFL, Renton WD

Pediatric Drugs, 2025

**MEDLINE search strategy**	**3**
**Excluded Studies table**	**5**
**Included Study Characteristics tables**	**11**

MEDLINE search strategy

1. exp *rheumatic diseases/
2. *Macrophage Activation Syndrome/
3. *Lymphohistiocytosis, Hemophagocytic/
4. exp *vasculitis/
5. exp *Hereditary Autoinflammatory Diseases/
6. exp *connective tissue diseases/
7. exp *sarcoidosis/
8. exp *myositis/
9. *Immunoglobulin G4-Related Disease/
10. (arthritis or Macrophage-activation-syndrome or Haemophagocytic-Lymphocytic-Histiocytosis or Vasculitis or Inflammatory-Central-Nervous-System-Disorder* or Behcet-Disease or Periodic-fever-syndrome* or Autoinflammatory-disease* or auto-inflammatory-disease* or autoinflammatory-syndrome or auto-inflammatory-syndrome or Familial-Mediterranean-Fever or Tumour-necrosis-associated-periodic-syndrome or Tumor-necrosis-associated-periodic-syndrome or Mevalonic-kinase-deficienc* or Hyperimmunoglobulinaemia-D or Cryoporin-associated-periodic-syndrome or Muckle-Wells-Syndrome or Neonatal-onset-multisystem-disease* or Chronic-infantile-neurological-cutaneous-and-articular-syndrome or Pyogenic-arthritis-with-pyoderma-gangrenosum-and-acne or Deficiency-of-adenosine-deaminase* or Syndrome-of-enterocolitis-and-auto-inflammation-associated-with-mutation-in-NLRC4 or Haploinsufficiency-of-A20 or Chronic-atypical-neutrophilic-dermatosis-with-lipodystrophy-and-elevated-temperature or STING-associated-vasculopathy-with-onset-in-early-infancy or Idiopathic-recurrent-pericarditis or Autoinflammatory-bone-disease* or Auto-inflammatory-bone-disease* or Chronic-recurrent-multifocal-osteomyelitis or Chronic-non-bacterial-osteomyelitis or Connective-tissue-disease* or sarcoidosis or Systemic-Lupus-Erythematosus or Dermatomyositis or Idiopathic-inflammatory-myopath* or systemic-sclerosis or Localised-scleroderma or Eosinophilic-fasciitis or Sjogren-Syndrome or Immunoglobulin-G4-disease*).tw,kf.
11. rheumat*.tw,kf,hw.
12. 1 or 2 or 3 or 4 or 5 or 6 or 7 or 8 or 9 or 10 or 11
13. *Interleukin 1 Receptor Antagonist Protein/
14. (9013duq28k or il-1ra or il1-febrile-inhibitor* or interleukin-1-receptor-antagonist-protein or urine- derived-il1-inhibitor* or anakinra or alta-2530 or alta2530 or amg-719 or amg719 or antril- or kineret or osp-101 or osp101 or p-130934 or p130934 or pb-ra-2010 or pbra2010 or raleukin).tw,kf.
15. (acz-885 or acz885 or cmab-816 or cmab816 or ilaris or canakinumab).tw,kf.
16. (arcalyst or il-1-trap or kpl-914 or kpl914 or rgn-303 or rgn303 or rilonacept).tw,kf.
17. 13 or 14 or 15 or 16
18. (newborn* or new-born* or baby or babies or neonat* or neo-nat* or infan* or toddler* or pre-schooler* or preschooler* or kinder or kinders or kindergarten* or kinder-aged or boy or boys or girl or girls or child* or pediatric* or paediatric* or school-age* or schoolage* or schoolchild* or schoolgirl* or schoolboy* or pre-teen* or

preteen* or tween or tweens or pre-adolescen* or preadolescen* or pre-puberty or prepuberty or pre-pubescen* or prepubescen* or adolescen* or youth or youths or teen or teens or teenage* or student* or young-adult* or young-people* or young-person* or emerging-adult* or emerging-people* or emerging-person* or AYA or AYAs or CAYA or CAYAs or juvenile).tw,kf,hw.

19. 12 and 17 and 18

20. (exp animals/ or (rat or rats or mouse or mice or rodent* or swine or porcine or murine or sheep or lamb or lambs or pig or pigs or piglet or piglets or rabbit or rabbits or cat or cats or dog or dogs or cattle or bovine or monkey or monkeys or trout or marmoset or marmosets).ti.) not human*.sh.

21. 19 not 20

22. limit 21 to (case reports or comment or editorial or guideline or letter or practice guideline or preprint)

23. 21 not 22

Excluded Studies table

Title	Authors	Year	DOI	Reason for exclusion
**Efficacy and safety of canakinumab as a second line biologic after tocilizumab treatment failure in children with systemic juvenile idiopathic arthritis: A single-centre cohort study using routinely collected health data**	Alexeeva, E.; Krekhova, E.; Dvoryakovskaya, T.; Isaeva, K.; Chomakhidze, A.; Chistyakova, E.; Lomakina, O.; Denisova, R.; Mamutova, A.; Fetisova, A.; Gautier, M.; Vankova, D.; Kriulin, I.; Saygitov, R.	2023	10.3389/fped.2023.1114207	Not enough follow up
**Systematic Review of Safety and Efficacy of IL-1-Targeted Biologics in Treating Immune-Mediated Disorders**	Arnold, D. D.; Yalamanoglu, A.; Boyman, O.	2022	10.3389/fimmu.2022.888392	Wrong study design
**Rilonacept pharmacokinetics in children with systemic juvenile idiopathic arthritis**	Autmizguine, J.; Cohen-Wolkowiez, M.; Ilowite, N.; Investigators, Rapport	2015	10.1002/jcph.372	Not enough follow up
**Unveiling the Efficacy, Safety, and Tolerability of Anti-Interleukin-1 Treatment in Monogenic and Multifactorial Autoinflammatory Diseases**	Bettiol, A.; Lopalco, G.; Emmi, G.; Cantarini, L.; Urban, M. L.; Vitale, A.; Denora, N.; Lopalco, A.; Cutrignelli, A.; Lopedota, A.; Venerito, V.; Fornaro, M.; Vannacci, A.; Rigante, D.; Cimaz, R.; Iannone, F.	2019	10.3390/ijms20081898	Wrong study design
**The risk of hospitalized infection following initiation of biologic agents versus methotrexate in the treatment of juvenile idiopathic arthritis**	Beukelman, T.; Xie, F.; Baddley, J. W.; Chen, L.; Mannion, M. L.; Saag, K. G.; Zhang, J.; Curtis, J. R.	2016	10.1186/s13075-016-1109-8	Not enough follow up
**On-demand anakinra treatment is effective in mevalonate kinase deficiency**	Bodar, E. J.; Kuijk, L. M.; Drenth, J. P.; van der Meer, J. W.; Simon, A.; Frenkel, J.	2011	10.1136/ard.2011.149922	Wrong age group
**The benefit-risk balance for biological agents in juvenile idiopathic arthritis: a meta-analysis of randomized clinical trials**	Cabrera, N.; Avila-Pedretti, G.; Belot, A.; Larbre, J. P.; Mainbourg, S.; Duquesne, A.; Janiaud, P.; Kassai, B.; Cucherat, M.; Lega, J. C.	2020	10.1093/rheumatology/keaa170	Wrong study design
**Safety profile of biologic agents for Behcet's disease in a multicenter observational cohort study**	Cantarini, L.; Talarico, R.; Generali, E.; Emmi, G.; Lopalco, G.; Costa, L.; Silvestri, E.; Caso, F.; Franceschini, R.; Cimaz, R.; Iannone, F.; Galeazzi, M.; Selmi, C.	2017	10.1111/1756-185X.12732	Wrong age group
**Efficacy of interleukin-1 targeting treatments in patients with familial mediterranean Fever**	Cetin, P.; Sari, I.; Sozeri, B.; Cam, O.; Birlik, M.; Akkoc, N.; Onen, F.; Akar, S.	2015	10.1007/s10753-014-0004-1	Wrong age group
**Effect of interleukin-1 inhibition in a cohort of patients with colchicine-resistant familial Mediterranean fever treated consecutively with anakinra and canakinumab**	Druyan, A.; Giat, E.; Livneh, A.; Grossman, C.; Ben-Zvi, I.; Lidar, M.	2021	10.55563/clinexprheumatol/rrr9zd	Wrong age group
**Efficacy and safety profile of anti-interleukin-1 treatment in Behcet's disease: a multicenter retrospective study**	Emmi, G.; Talarico, R.; Lopalco, G.; Cimaz, R.; Cantini, F.; Viapiana, O.; Olivieri, I.; Goldoni, M.; Vitale, A.; Silvestri, E.; Prisco, D.; Lapadula, G.; Galeazzi, M.; Iannone, F.; Cantarini, L.	2016	10.1007/s10067-015-3004-0	Wrong age group
**Efficacy and safety of anti-interleukin-1 in children with colchicine-resistant familial Mediterranean fever**	Erkilet, H. K.; Gezgin Yildirim, D.; Esmeray, P.; Soylemezoglu, O.	2023	10.1111/ped.15588	Not enough follow up

**Canakinumab treatment for patients with active recurrent or chronic TNF receptor-associated periodic syndrome (TRAPS): an open-label, phase II study**	Gattorno, M.; Obici, L.; Cattalini, M.; Tormey, V.; Abrams, K.; Davis, N.; Speziale, A.; Bhansali, S. G.; Martini, A.; Lachmann, H. J.	2017	10.1136/annrheumdis-2015-209031	Wrong age group
**Long-Term Efficacy and Safety of Canakinumab in Patients With Tumor Necrosis Factor Receptor-Associated Periodic Syndrome: Results From a Phase III Trial**	Gattorno, M.; Obici, L.; Penades, I. C.; Kallinich, T.; Benseler, S.; Dekker, E.; Levy, J.; De Benedetti, F.; Lachmann, H.	2024	10.1002/art.42695	Wrong age group
**Neonatal-onset multisystem inflammatory disease responsive to interleukin-1beta inhibition**	Goldbach-Mansky, R.; Dailey, N. J.; Canna, S. W.; Gelabert, A.; Jones, J.; Rubin, B. I.; Kim, H. J.; Brewer, C.; Zalewski, C.; Wiggs, E.; Hill, S.; Turner, M. L.; Karp, B. I.; Aksentijevich, I.; Pucino, F.; Penzak, S. R.; Haverkamp, M. H.; Stein, L.; Adams, B. S.; Moore, T. L.; Fuhlbrigge, R. C.; Shaham, B.; Jarvis, J. N.; O'Neil, K.; Vehe, R. K.; Beitz, L. O.; Gardner, G.; Hannan, W. P.; Warren, R. W.; Horn, W.; Cole, J. L.; Paul, S. M.; Hawkins, P. N.; Pham, T. H.; Snyder, C.; Wesley, R. A.; Hoffmann, S. C.; Holland, S. M.; Butman, J. A.; Kastner, D. L.	2006	10.1056/NEJMoa055137	Not enough follow up
**Rate and Clinical Presentation of Macrophage Activation Syndrome in Patients With Systemic Juvenile Idiopathic Arthritis Treated With Canakinumab**	Grom, A. A.; Ilowite, N. T.; Pascual, V.; Brunner, H. I.; Martini, A.; Lovell, D.; Ruperto, N.; Paediatric Rheumatology International Trials, Organisation; the Pediatric Rheumatology Collaborative Study, Group; Leon, K.; Lheritier, K.; Abrams, K.	2016	10.1002/art.39407	Wrong study design
**The familial Mediterranean fever (FMF) 50 score: does it work in a controlled clinical trial? Re-analysis of the trial of rilonacept for patients with colchicine-resistant or intolerant FMF**	Hashkes, P. J.; Huang, B.	2015	-	No safety outcomes
**Anakinra: a safe and effective first-line treatment in systemic onset juvenile idiopathic arthritis (SoJIA)**	Hedrich, C. M.; Bruck, N.; Fiebig, B.; Gahr, M.	2012	10.1007/s00296-011-2249-4	Not enough participants
**Long-term efficacy and safety profile of rilonacept in the treatment of cryopryin-associated periodic syndromes: results of a 72-week open-label extension study**	Hoffman, H. M.; Throne, M. L.; Amar, N. J.; Cartwright, R. C.; Kivitz, A. J.; Soo, Y.; Weinstein, S. P.	2012	10.1016/j.clinthera.2012.09.009	Wrong age group
**Biologic-associated infections in pediatric rheumatology**	Horneff, G.	2015	10.1007/s11926-015-0542-z	Wrong study design
**Real-world safety and effectiveness of canakinumab in patients with cryopyrin-associated periodic fever syndrome: a long-term observational study in Japan**	Hosono, K.; Kato, C.; Sasajima, T.	2022	10.55563/clinexprheumatol/pjs6eh	Wrong age group
**Real-world safety and effectiveness of canakinumab in patients with tumour necrosis factor receptor-associated periodic syndrome or hyperimmunoglobulinaemia D syndrome: Interim results from post-marketing surveillance in Japan**	Hosono, K.; Matsumoto, K.; Shimbo, M.; Tsumiyama, I.; Kato, C.	2023	10.1093/mr/roac041	Wrong age group
**Anakinra treatment for refractory cerebral autoinflammatory responses**	Jang, Y.; Lee, W. J.; Lee, H. S.; Chu, K.; Lee, S. K.; Lee, S. T.	2022	10.1002/acn3.51500	Wrong age group

**Efficacy and safety of anti-interleukin-1 treatment in familial Mediterranean fever patients: a systematic review and meta-analysis**	Kilic, B.; Guler, Y.; Azman, F. N.; Bostanci, E.; Ugurlu, S.	2024	10.1093/rheumatology/kead514	Wrong study design
**Efficacy and safety of biologic drugs in Still's disease: a systematic review and network meta-analysis of randomized controlled trials**	Kilic, B.; Ozturk, A.; Karup, S.; Hacioglu, E.; Ugurlu, S.	2025	10.1093/rheumatology/keae295	Wrong study design
**Drug reactions in children with rheumatic diseases receiving parenteral therapies: 9 years' experience of a tertiary pediatric rheumatology center**	Koc, R.; Sonmez, H. E.; Cakan, M.; Karadag, S. G.; Tanatar, A.; Cakmak, F.; Aktay Ayaz, N.	2020	10.1007/s00296-019-04498-z	Not enough follow up
**Treatment of hyperimmunoglobulinemia D syndrome with biologics in children: review of the literature and Finnish experience**	Kostjukovits, S.; Kalliokoski, L.; Antila, K.; Korppi, M.	2015	10.1007/s00431-015-2505-9	Wrong study design
**Anti-interleukin-1 treatment in 26 patients with refractory familial mediterranean fever**	Kucuksahin, O.; Yildizgoren, M. T.; Ilgen, U.; Ates, A.; Kinikli, G.; Turgay, M.; Erten, S.	2017	10.1080/14397595.2016.1194510	Wrong age group
**A systematic literature review of efficacy, effectiveness and safety of biologic therapies for treatment of familial Mediterranean fever**	Kuemmerle-Deschner, J. B.; Gautam, R.; George, A. T.; Raza, S.; Lomax, K. G.; Hur, P.	2020	10.1093/rheumatology/keaa205	Wrong study design
**Two-year results from an open-label, multicentre, phase III study evaluating the safety and efficacy of canakinumab in patients with cryopyrin-associated periodic syndrome across different severity phenotypes**	Kuemmerle-Deschner, J. B.; Hachulla, E.; Cartwright, R.; Hawkins, P. N.; Tran, T. A.; Bader-Meunier, B.; Hoyer, J.; Gattorno, M.; Gul, A.; Smith, J.; Leslie, K. S.; Jimenez, S.; Morell-Dubois, S.; Davis, N.; Patel, N.; Widmer, A.; Preiss, R.; Lachmann, H. J.	2011	10.1136/ard.2011.152728	Wrong age group
**Phenotype, genotype, and sustained response to anakinra in 22 patients with autoinflammatory disease associated with CIAS-1/NALP3 mutations**	Leslie, K. S.; Lachmann, H. J.; Bruning, E.; McGrath, J. A.; Bybee, A.; Gallimore, J. R.; Roberts, P. F.; Woo, P.; Grattan, C. E.; Hawkins, P. N.	2006	10.1001/archderm.142.12.1591	Wrong age group
**Safety and Efficacy Evaluation of PerkinRA in Comparison with Kineret in Systemic Juvenile Idiopathic Arthritis Patients**	Mirzaee, Azadeh Zeinab; Sadeghi, Setayesh; Shiari, Reza; Ziaee, Vahid; Karagah, Amirhossein; Sinaei, Reza; Ghotbabadi, Shabnam Hajiani; Tahghighi, Fatemeh; Fathi, Mohammad Reza; Shirvani, Armin; Miremarati, Aye; Ghasemi, Leila; Rahmani, Khosro; Araghi, Shahram; Parvaneh, Vadood Javadi; Jaffaraghaei, Morteza; Mohammadkarim, Alireza	2024	10.4103/bbrj.bbrj_155_24	Not enough follow up
**Clinical Characteristics of Cryopyrin-Associated Periodic Syndrome and Long-Term Real-World Efficacy and Tolerability of Canakinumab in Japan: Results of a Nationwide Survey**	Miyamoto, T.; Izawa, K.; Masui, S.; Yamazaki, A.; Yamasaki, Y.; Matsubayashi, T.; Shiraki, M.; Ohnishi, H.; Yasumura, J.; Kawabe, T.; Miyamae, T.; Matsubara, T.; Arakawa, N.; Ishige, T.; Takizawa, T.; Shimbo, A.; Shimizu, M.; Kimura, N.; Maeda, Y.; Maruyama, Y.; Shigemura, T.; Furuta, J.; Sato, S.; Tanaka, H.; Izumikawa, M.; Yamamura, M.; Hasegawa, T.; Kaneko, H.; Nakagishi, Y.; Nakano, N.; Iida, Y.; Nakamura, T.; Wakiguchi, H.; Hoshina, T.; Kawai, T.; Murakami, K.; Akizuki, S.; Morinobu, A.; Ohmura, K.; Eguchi, K.; Sonoda, M.; Ishimura, M.; Furuno, K.; Kashiwado, M.; Mori, M.; Kawahata, K.; Hayama, K.; Shimoyama, K.; Sasaki, N.; Ito, T.; Umebayashi, H.; Omori, T.; Nakamichi, S.; Dohmoto, T.; Hasegawa, Y.; Kawashima, H.; Watanabe, S.; Taguchi, Y.; Nakaseko, H.; Iwata,	2024	10.1002/art.42808	Wrong age group

	N.; Kohno, H.; Ando, T.; Ito, Y.; Kataoka, Y.; Saeki, T.; Kaneko, U.; Murase, A.; Hattori, S.; Nozawa, T.; Nishimura, K.; Nakano, R.; Watanabe, M.; Yashiro, M.; Komai, T.; Kato, K.; Honda, Y.; Hiejima, E.; Yonezawa, A.; Bessho, K.; Okada, S.; Ohara, O.; Takita, J.; Yasumi, T.; Nishikomori, R.; Japan, Caps Working Group			
**Long-Term Safety and Effectiveness of Canakinumab in Patients with MKD/HIDS: Interim Analysis of the RELIANCE Registry**	Oommen, P. T.; Kallinich, T.; Rech, J.; Blank, N.; Weber-Arden, J.; Kuemmerle-Deschner, J. B.	2025	10.1007/s40744-024-00733-7	Not enough participants
**Effectiveness and safety of a second and third biological agent after failing etanercept in juvenile idiopathic arthritis: results from the Dutch National ABC Register**	Otten, M. H.; Prince, F. H.; Anink, J.; Ten Cate, R.; Hoppenreijs, E. P.; Armbrust, W.; Koopman-Keemink, Y.; van Pelt, P. A.; Kamphuis, S.; Gorter, S. L.; Dolman, K. M.; Swart, J. F.; van den Berg, J. M.; Wulffraat, N. M.; van Rossum, M. A.; van Suijlekom-Smit, L. W.	2013	10.1136/annrheumdis-2011-201060	No safety outcomes
**A multicentre, randomised, double-blind, placebo-controlled trial with the interleukin-1 receptor antagonist anakinra in patients with systemic-onset juvenile idiopathic arthritis (ANAJIS trial)**	Quartier, P.; Allantaz, F.; Cimaz, R.; Pillet, P.; Messiaen, C.; Bardin, C.; Bossuyt, X.; Boutten, A.; Bienvenu, J.; Duquesne, A.; Richer, O.; Chaussabel, D.; Mogenet, A.; Banchereau, J.; Treluyer, J. M.; Landais, P.; Pascual, V.	2011	10.1136/ard.2010.134254	Not enough follow up
**Combination therapy of abatacept and anakinra in children with refractory systemic juvenile idiopathic arthritis: a retrospective case series**	Record, J. L.; Beukelman, T.; Cron, R. Q.	2011	10.3899/jrheum.100726	Not enough participants
**Tolerance and efficacy of off-label anti-interleukin-1 treatments in France: a nationwide survey**	Rossi-Semerano, L.; Fautrel, B.; Wendling, D.; Hachulla, E.; Galeotti, C.; Semerano, L.; Touitou, I.; Kone-Paut, I.; CRI, Mail study Group on behalf of	2015	10.1186/s13023-015-0228-7	Wrong age group
**Two randomized trials of canakinumab in systemic juvenile idiopathic arthritis**	Ruperto, N.; Brunner, H. I.; Quartier, P.; Constantin, T.; Wulffraat, N.; Horneff, G.; Brik, R.; McCann, L.; Kasapcopur, O.; Rutkowska-Sak, L.; Schneider, R.; Berkun, Y.; Calvo, I.; Erguven, M.; Goffin, L.; Hofer, M.; Kallinich, T.; Oliveira, S. K.; Uziel, Y.; Viola, S.; Nistala, K.; Wouters, C.; Cimaz, R.; Ferrandiz, M. A.; Flato, B.; Gamir, M. L.; Kone-Paut, I.; Grom, A.; Magnusson, B.; Ozen, S.; Sztajnbok, F.; Lheritier, K.; Abrams, K.; Kim, D.; Martini, A.; Lovell, D. J.; Printo; Prcsg	2012	10.1056/NEJMoa1205099	Not enough follow up
**Long-term renal outcome of Cryopyrin-associated periodic syndrome (CAPS) under anti-Interleukin-1 therapy**	Russwurm, M.; Johannsen, S.; Kortus-Gotze, B.; Haas, C. S.	2024	10.1038/s41598-024-67380-4	Wrong age group
**Systemic onset juvenile idiopathic arthritis: a single center experience**	Sag, E.; Uzunoglu, B.; Bal, F.; Sonmez, H. E.; Demir, S.; Bilginer, Y.; Ozen, S.	2019	10.24953/turkjped.2019.06.005	No safety outcomes
**Assessment of effectiveness of anakinra and canakinumab in patients with colchicine-resistant/unresponsive familial Mediterranean fever**	Sahin, A.; Derin, M. E.; Albayrak, F.; Karakas, B.; Karagoz, Y.	2020	10.1186/s42358-020-0117-1	Wrong age group
**Effect of Biologic Therapy on Clinical and Laboratory Features of Macrophage Activation Syndrome Associated With Systemic Juvenile Idiopathic Arthritis**	Schulert, G. S.; Minoia, F.; Bohnsack, J.; Cron, R. Q.; Hashad, S.; Kon, E. Paut I.; Kostik, M.; Lovell, D.; Maritsi, D.; Nigrovic, P. A.; Pal, P.; Ravelli, A.; Shimizu, M.; Stanevicha, V.; Vastert, S.; Woerner, A.; de Benedetti, F.; Grom, A. A.	2018	10.1002/acr.23277	Wrong study design

**The genetic and clinical characteristics and effects of Canakinumab on cryopyrin-associated periodic syndrome: a large pediatric cohort study from China**	Shu, Z.; Zhang, Y.; Han, T.; Li, Y.; Piao, Y.; Sun, F.; Ma, J.; Mo, W.; Sun, J.; Chan, K. W.; Yang, W.; Lau, Y. L.; Mao, H.	2023	10.3389/fimmu.2023.1267933	No safety outcomes
**A 24-month open-label study of canakinumab in neonatal-onset multisystem inflammatory disease**	Sibley, C. H.; Chioato, A.; Felix, S.; Colin, L.; Chakraborty, A.; Plass, N.; Rodriguez-Smith, J.; Brewer, C.; King, K.; Zalewski, C.; Kim, H. J.; Bishop, R.; Abrams, K.; Stone, D.; Chapelle, D.; Kost, B.; Snyder, C.; Butman, J. A.; Wesley, R.; Goldbach-Mansky, R.	2015	10.1136/annrheumdis-2013-204877	Not enough participants
**Comparison of the efficacy and safety of biological agents in patients with systemic juvenile idiopathic arthritis: A Bayesian network meta-analysis of randomized controlled trials**	Song, G. G.; Lee, Y. H.	2021	10.5414/CP203791	Wrong study design
**Drug survival of anakinra and canakinumab in monogenic autoinflammatory diseases: observational study from the International AIDA Registry**	Sota, J.; Rigante, D.; Cimaz, R.; Cattalini, M.; Frassi, M.; Manna, R.; Sicignano, L. L.; Verrecchia, E.; Aragona, E.; Maggio, M. C.; Lopalco, G.; Emmi, G.; Parronchi, P.; Cauli, A.; Wiesik-Szewczyk, E.; Hernandez-Rodriguez, J.; Gaggiano, C.; Tarsia, M.; Mourabi, M.; Ragab, G.; Vitale, A.; Fabiani, C.; Frediani, B.; Lamacchia, V.; Renieri, A.; Cantarini, L.; Autoinflammatory Diseases, Alliance; the Autoinflammatory Diseases Working Group of the Italian Society of, Rheumatology	2021	10.1093/rheumatology/keab419	Wrong age group
**Safety profile of the interleukin-1 inhibitors anakinra and canakinumab in real-life clinical practice: a nationwide multicenter retrospective observational study**	Sota, J.; Vitale, A.; Insalaco, A.; Sfriso, P.; Lopalco, G.; Emmi, G.; Cattalini, M.; Manna, R.; Cimaz, R.; Priori, R.; Talarico, R.; de Marchi, G.; Frassi, M.; Gallizzi, R.; Soriano, A.; Alessio, M.; Cammelli, D.; Maggio, M. C.; Gentileschi, S.; Marcolongo, R.; La Torre, F.; Fabiani, C.; Colafrancesco, S.; Ricci, F.; Galozzi, P.; Viapiana, O.; Verrecchia, E.; Pardeo, M.; Cerrito, L.; Cavallaro, E.; Olivieri, A. N.; Paolazzi, G.; Vitiello, G.; Maier, A.; Silvestri, E.; Stagnaro, C.; Valesini, G.; Mosca, M.; de Vita, S.; Tincani, A.; Lapadula, G.; Frediani, B.; De Benedetti, F.; Iannone, F.; Punzi, L.; Salvarani, C.; Galeazzi, M.; Angotti, R.; Messina, M.; Tosi, G. M.; Rigante, D.; Cantarini, L.; Working Group" of Systemic Autoinflammatory Diseases of, S. I. R.	2018	10.1007/s10067-018-4119-x	Wrong age group
**Evaluation of clinical outcomes in systemic juvenile idiopathic arthritis patients treated with biological agents in Turkey: the TURSIS study**	Sozeri, B.; Demir, F.; Barut, K.; Atalay, E.; Pac Kisaarslan, A.; Ozdel, S.; Altug Gucenmez, O.; Makay, B.; Aktay Ayaz, N.; Haslak, F.; Sag, E.; Yildiz, M.; Kaya Akca, U.; Adrovic, A.; Bilginer, Y.; Poyrazoglu, H.; Unsal, E.; Kasapcopur, O.; Ozen, S.	2024	10.55563/clinexprheumatol/j611kr	No safety outcomes
**What are the immunological consequences of long-term use of biological therapies for juvenile idiopathic arthritis?**	Swart, J. F.; de Roock, S.; Wulffraat, N. M.	2013	10.1186/ar4213	Wrong study design
**Occurrence of adverse events in patients with JIA receiving biologic agents: long-term follow-up in a real-life setting**	Tarkiainen, M.; Tynjala, P.; Vahasalo, P.; Lahdenne, P.	2015	10.1093/rheumatology/keu457	Not enough participants
**Efficacy and safety of biological agents for systemic juvenile idiopathic arthritis: a systematic review and meta-analysis of randomized trials**	Tarp, S.; Amarilyo, G.; Foeldvari, I.; Christensen, R.; Woo, J. M.; Cohen, N.; Pope, T. D.; Furst, D. E.	2016	10.1093/rheumatology/kev382	Wrong study design

**Anakinra treatment in patients with familial Mediterranean fever: a single-centre experience**	Ugurlu, S.; Ergezen, B.; Egeli, B. H.; Selvi, O.; Ozdogan, H.	2021	10.1093/rheumatology/keaa596	Wrong age group
**The use of biologic response modifiers in polyarticular-course juvenile idiopathic arthritis: a systematic review**	Ungar, W. J.; Costa, V.; Burnett, H. F.; Feldman, B. M.; Laxer, R. M.	2013	10.1016/j.semarthrit.2012.10.006	Wrong study design
**Effect of interleukin-1 antagonists on the quality of life in familial Mediterranean fever patients**	Varan, O.; Kucuk, H.; Babaoglu, H.; Atas, N.; Salman, R. B.; Satis, H.; Ozturk, M. A.; Haznedaroglu, S.; Goker, B.; Tufan, A.	2019	10.1007/s10067-018-4384-8	Wrong age group
**Real-Life Indications of Interleukin-1 Blocking Agents in Hereditary Recurrent Fevers: Data From the JIRcohort and a Literature Review**	Vinit, C.; Georgin-Lavialle, S.; Theodoropoulou, A.; Barbier, C.; Belot, A.; Mejbri, M.; Pillet, P.; Pachlopnik, J.; Poignant, S.; Rebelle, C.; Woerner, A.; Kone-Paut, I.; Hentgen, V.	2021	10.3389/fimmu.2021.744780	No safety outcomes
**A Snapshot on the On-Label and Off-Label Use of the Interleukin-1 Inhibitors in Italy among Rheumatologists and Pediatric Rheumatologists: A Nationwide Multi-Center Retrospective Observational Study**	Vitale, A.; Insalaco, A.; Sfriso, P.; Lopalco, G.; Emmi, G.; Cattalini, M.; Manna, R.; Cimaz, R.; Priori, R.; Talarico, R.; Gentileschi, S.; de Marchi, G.; Frassi, M.; Gallizzi, R.; Soriano, A.; Alessio, M.; Cammelli, D.; Maggio, M. C.; Marcolongo, R.; La Torre, F.; Fabiani, C.; Colafrancesco, S.; Ricci, F.; Galozzi, P.; Viapiana, O.; Verrecchia, E.; Pardeo, M.; Cerrito, L.; Cavallaro, E.; Olivieri, A. N.; Paolazzi, G.; Vitiello, G.; Maier, A.; Silvestri, E.; Stagnaro, C.; Valesini, G.; Mosca, M.; de Vita, S.; Tincani, A.; Lapadula, G.; Frediani, B.; De Benedetti, F.; Iannone, F.; Punzi, L.; Salvarani, C.; Galeazzi, M.; Rigante, D.; Cantarini, L.	2016	10.3389/fphar.2016.00380	Wrong age group
**Long-term safety and effectiveness of canakinumab therapy in patients with cryopyrin-associated periodic syndrome: results from the beta-Confident Registry**	Walker, U. A.; Tilson, H. H.; Hawkins, P. N.; Poll, T. V.; Noviello, S.; Levy, J.; Vritzali, E.; Hoffman, H. M.; Kuemmerle-Deschner, J. B.; Investigators, Cacz D. Study	2021	10.1136/rmdopen-2021-001663	Wrong age group
**Comparative efficacy and safety of different drugs in patients with systemic juvenile idiopathic arthritis: A systematic review and network meta-analysis**	Wang, B.; Zhang, Y.; Zhao, Z.; Ping, J.; Zhou, L.; Wang, Y.	2024	10.1097/MD.0000000000038002	Wrong study design
**Interventions for reducing inflammation in familial Mediterranean fever**	Yin, X.; Tian, F.; Wu, B.; Xu, T.	2022	10.1002/14651858.CD010893.pub4	Wrong study design
**Anakinra for systemic juvenile arthritis: the Rocky Mountain experience**	Zeft, A.; Hollister, R.; LaFleur, B.; Sampath, P.; Soep, J.; McNally, B.; Kunkel, G.; Schlesinger, M.; Bohnsack, J.	2009	10.1097/RHU.0b013e3181a4f459	Not enough follow up
**Canakinumab in the treatment of systemic juvenile idiopathic arthritis: a retrospective single center study in China**	Zhu, X.; Weng, R.; Huang, Y.; Xu, Y.; Yang, J.; He, T.	2024	10.3389/fped.2024.1349907	Not enough follow up

Included Study Characteristics tables

**Study**	Adıgüzel Dundar 2024				
**Primary reference**	Adıgüzel Dundar H, Makay B, Altuğ Gücenmez Ö, Türkuçar S, Bayram SN, Belet N, et al. Latent Tuberculosis Infection in Children with Pediatric Rheumatologic Diseases Treated with Canakinumab. J Pediatr Infect. 2024;18(3):168-74.				
**Associated references**	Nil		**Study identifier**	Nil	
**Study characteristics**					
**Date of study**	Feb 2012 to Mar 2020	**Design**	Retrospective cohort study	**Condition**	FMF 40, SJIA 16, MKD 6, CAPS 5
**Medication**	CAN (67)	**Total number of patients (IL-1 treated)**	67 (67)	**Age**	Median 14.0 years
**Follow-up**	Median 6 years	**Country**	Türkiye	**Sex**	28 F / 39 M
**Author's conclusions**	This study suggests that although frequency of latent TB infection in children treated with CAN is not low in a TB endemic country, progression to TB disease under CAN treatment is not a common finding in our study.				
**Outcomes**					
**Infection**	LTBI screening prior to CAN: positive TST 9/67 (13.4%), positive QFT-G test 1/11 (9%), isoniazid prophylaxis 10/67 (14.9%). During CAN: positive TST 11/56 (19.6%), positive QFT-G test 2/22 (9%), isoniazid prophylaxis 13/67 (19.4%). Total: LTBI positive 23/67 (34.3). No episodes of active MTB infection.				
**Malignancy**	Not reported.				
**ILD**	Not reported.				
**Drug reaction**	Not reported.				
**SAE**	Not reported.				
**Discontinuation**	Not reported.				
**Death**	Not reported.				
**Additional notes**	Nil				
**Risk of bias (<6 high, 6-11 moderate, >11 low risk of bias)**					
**Aim**	2	**Consecutive**	1	**Prospective**	0

**Endpoints**	2	**Assessment**	0	**Follow-up**	2
**Loss to follow-up**	1	**Calculation**	0	**Total**	8 (moderate)
CAN canakinumab, FMF familial mediterranean fever, ILD interstitial lung disease, LTBI latent tuberculosis infection, MKD mevalonate kinase deficiency, MTB mycobacterium tuberculosis, QFT-G quantiferon gold, SAE serious adverse event, TST tuberculin skin test					

**Study**	Al-Mayouf 2016				
**Primary reference**	Al-Mayouf SM, Alenazi A, AlJasser H. Biologic agents therapy for Saudi children with rheumatic diseases: indications and safety. Int J Rheum Dis. 2016;19(6):600-5.				
**Associated references**	Nil		**Study identifier**	Nil	
**Study characteristics**					
**Date of study**	Jan 2001 to Dec 2011	**Design**	Retrospective cohort study	**Condition**	JIA (15), CAPS (5), vasculitis (1), NMO (2)
**Medication**	ANA (23)	**Total number of patients (IL-1 treated)**	134 (23)	**Age**	Not reported
**Follow-up**	Not reported	**Country**	Saudi Arabia	**Sex**	Not reported
**Author's conclusions**	Biologic agents were used in children with a range of rheumatic diseases. Of these, the most frequent was JIA. Off-label use of biologic agents in our cohort is common. These agents seem safe. However, they may associated with various adverse events. Sequential therapy seems well tolerated. However, this should be carefully balanced and considered on an individual basis.				
**Outcomes**					
**Infection**	5 infections including 2 septic shock with gram-negative sepsis.				
**Malignancy**	Not reported.				
**ILD**	Not reported.				
**Drug reaction**	3 local reaction.				
**SAE**	Not reported.				
**Discontinuation**	Not reported.				
**Death**	2 deaths from septic shock with gram-negative sepsis.				
**Additional notes**	Nil.				

Risk of bias (<6 high, 6-11 moderate, >11 low risk of bias)					
**Aim**	1	**Consecutive**	2	**Prospective**	0
**Endpoints**	2	**Assessment**	0	**Follow-up**	0
**Loss to follow-up**	0	**Calculation**	0	**Total**	5 (high)
ANA anakinra, CAPS cryopyrin associated periodic syndromes, JIA juvenile idiopathic arthritis, ILD interstitial lung disease, NMO neuromyelitis optica, SAE serious adverse event					

**Study**	Alexeeva 2023				
**Primary reference**	Alexeeva E, Shingarova M, Dvoryakovskaya T, Lomakina O, Fetisova A, Isaeva K, et al. Safety and efficacy of canakinumab treatment for undifferentiated autoinflammatory diseases: the data of a retrospective cohort two-centered study. Front Med (Lausanne). 2023;10:1257045.				
**Associated references**	Nil		**Study identifier**	Nil	
**Study characteristics**					
**Date of study**	2013 to 2022	**Design**	Retrospective cohort study	**Condition**	Undifferentiated autoinflammatory diseases
**Medication**	CAN (32)	**Number of patients (IL-1 treated)**	32 (32)	**Age**	Median age 2.5 years (0.08-16.7)
**Follow-up**	Median 3.5 years (IQR 1.3-5.2)	**Country**	Russia	**Sex**	13 F / 19 M
**Author's conclusions**	The treatment of patients with uAIDs using canakinumab was safe and effective. Further randomized clinical trials are required to confirm the efficacy and safety.				
**Outcomes**					
**Infection**	5 patients had mild respiratory infections.				
**Malignancy**	Not reported.				
**ILD**	Not reported.				
**Drug reaction**	Not reported.				
**SAE**	No SAEs.				
**Discontinuation**	Not reported.				

**Death**	Not reported.				
**Additional notes**	1 case of leucopenia but discontinuation was not required.				
**Risk of bias (<6 high, 6-11 moderate, >11 low risk of bias)**					
**Aim**	2	**Consecutive**	1	**Prospective**	0
**Endpoints**	1	**Assessment**	0	**Follow-up**	1
**Loss to follow-up**	0	**Calculation**	0	**Total**	5 (high)
CAN canakinumab, ILD interstitial lung disease, IQR interquartile range, SAE serious adverse event					

**Study**	Atemnkeng Ntam 2021				
**Primary reference**	Atemnkeng Ntam V, Klein A, Horneff G. Safety and efficacy of anakinra as first-line or second-line therapy for systemic onset juvenile idiopathic arthritis - data from the German BIKER registry. Expert Opin Drug Saf. 2021;20(1):93-100.				
**Associated references**	Nil		**Study identifier**	Nil	
**Study characteristics**					
**Date of study**	Until Dec 2018	**Design**	Registry	**Condition**	SJIA
**Medication**	ANA (51)	**Number of patients (IL-1 treated)**	51 (51)	**Age**	First line 7.3 years (SD 4.5)<br>Second line 5 years (SD 3.7)
**Follow-up**	12 months	**Country**	Germany	**Sex**	19 F / 32 M
**Author's conclusions**	This analysis adds to the established safety profile of anakinra and demonstrates that anakinra is effective as first-line or second-line treatment.				
**Outcomes**					
**Infection**	22 events (17 infectious AE and 5 infectious SAE).				
**Malignancy**	Nil. 1 patient leukemia 3 years later thought to be unrelated.				
**ILD**	Not reported.				
**Drug reaction**	Nil.				

**SAE**	8 (1 MAS, 5 infections/ infestations, 1 general/administration, 1 musculoskeletal/ connective tissue)				
**Discontinuation**	1 discontinuation due to serious/adverse events.				
**Death**	No deaths.				
**Additional notes**	NIl.				
**Risk of bias (<6 high, 6-11 moderate, >11 low risk of bias)**					
**Aim**	2	**Consecutive**	2	**Prospective**	1
**Endpoints**	2	**Assessment**	0	**Follow-up**	0
**Loss to follow-up**	0	**Calculation**	0	**Total**	7 (moderate)
AE adverse event, ANA anakinra, ILD interstitial lung disease, MAS macrophage activation syndrome, SAE serious adverse event, SD standard deviation, SJIA systemic onset juvenile idiopathic arthritis					

**Study**	Balci 2020				
**Primary reference**	Balci S, Demir I, Serbes M, Dogruel D, Altintas DU, Ekinci RMK. Retrospective analyzes of adverse events during biologic agents in children with juvenile idiopathic arthritis from a single center in Turkey. Reumatologia. 2020;58(6):367-74.				
**Associated references**	Nil		**Study identifier**	Nil	
**Study characteristics**					
**Date of study**	August 2008 to March 2019	**Design**	Retrospective cohort study	**Condition**	JIA
**Medication**	ANA (2)<br>CAN (13)	**Number of patients (IL-1 treated)**	162 (15)	**Age**	10.5 years (SD 4.3)
**Follow-up**	Mean follow up 19.7 months (SD 2.1)	**Country**	Türkiye	**Sex**	90 F / 72 M
**Author's conclusions**	While the most frequent adverse events during biologic agents was upper respiratory tract infections, the frequency of serious adverse events was 6.7%; therefore, juvenile idiopathic arthritis patients receiving biologic agents should be carefully evaluated for these adverse events in clinical practice.				
**Outcomes**					
**Infection**	6 URTI, 6 UTI, 2 pneumonia, 2 TB, 1 herpes labialis, abscess 2, impetigo 1, chickenpox 1.				
**Malignancy**	Nil.				

**ILD**	Not reported.				
**Drug reaction**	3 injection site reactions, 0 anaphylaxis.				
**SAE**	Not reported.				
**Discontinuation**	Not reported.				
**Death**	Nil.				
**Additional notes**	Nil.				
**Risk of bias (<6 high, 6-11 moderate, >11 low risk of bias)**					
**Aim**	2	**Consecutive**	0	**Prospective**	0
**Endpoints**	2	**Assessment**	0	**Follow-up**	0
**Loss to follow-up**	0	**Calculation**	0	**Total**	4 (high)
ANA anakinra, CAN canakinumab, ILD interstitial lung disease, JIA juvenile idiopathic arthritis, MAS macrophage activation syndrome, SAE serious adverse event, SD standard deviation, TB tuberculosis, URTI upper respiratory tract infection, UTI urinary tract infection					

**Study**	Berdeli 2019				
**Primary reference**	Berdeli A, Senol O, Talay G. Treatment of familial mediterranean fever with canakinumab in patients who are unresponsive to colchicine. Eur J Rheumatol. 2019;6(2):85-8.				
**Associated references**	Nil		**Study identifier**	Nil	
**Study characteristics**					
**Date of study**	Not reported	**Design**	Retrospective cohort study	**Condition**	FMF
**Medication**	CAN (22)	**Number of patients (IL-1 treated)**	22 (22)	**Age**	13.8 year (SD 4.0)
**Follow-up**	15.9 months (SD 8.8)	**Country**	Türkiye	**Sex**	13 M / 9 F
**Author's conclusions**	Canakinumab is an effective and safe anti-IL1 agent to reduce attacks in patients with FMF with no response to colchicine and to reduce the level of high-level laboratory findings associated with FMF.				
**Outcomes**					
**Infection**	There were no opportunistic infections, cases of tuberculosis … observed in our patients.				

**Malignancy**	Not reported.				
**ILD**	Not reported.				
**Drug reaction**	Not reported.				
**SAE**	Not reported.				
**Discontinuation**	Not reported.				
**Death**	There were no deaths observed in our patients.				
**Additional notes**	There were no adverse effects of drug.				
**Risk of bias (<6 high, 6-11 moderate, >11 low risk of bias)**					
**Aim**	2	**Consecutive**	0	**Prospective**	0
**Endpoints**	1	**Assessment**	1	**Follow-up**	0
**Loss to follow-up**	0	**Calculation**	0	**Total**	4 (high)
CAN canakinumab, FMF familial Mediterranean fever, ILD interstitial lung disease, SAE serious adverse event, SD standard deviation					

**Study**	Brogan 2019				
**Primary reference**	Brogan PA, Hofer M, Kuemmerle-Deschner JB, Kone-Paut I, Roesler J, Kallinich T, et al. Rapid and Sustained Long-Term Efficacy and Safety of Canakinumab in Patients With Cryopyrin-Associated Periodic Syndrome Ages Five Years and Younger. Arthritis rheumatol. 2019;71(11):1955-63.				
**Associated references**	Nil		**Study identifier**	NCT01302860<br>NCT01576367	
**Study characteristics**					
**Date of study**	Nov 2010 to Oct 2015	**Design**	Prospective open label study	**Condition**	CAPS
**Medication**	CAN (17)	**Number of patients (IL-1 treated)**	17 (17)	**Age**	31 months (range 1-59)
**Follow-up**	44 PY (mean 951 days)	**Country**	Germany, Belgium, Spain, France, Switzerland, United Kingdom, Canada	**Sex**	12 M / 5 F

**Author's conclusions**	Our findings indicate that canakinumab effectively maintains efficacy through 152 weeks and appears to have no effect on the ability to produce antibodies against standard childhood non- live vaccines. The safety profile of canakinumab was consistent with previous studies, supporting long- term use of canakinumab for CAPS in children ≤5 years of age.				
**Outcomes**					
**Infection**	Open label trial: asopharyngitis 7 (41%), upper respiratory tract infection 7 (41%), diarrhoea 1 (5.9%%), pyrexia 6 (35%), rhinitis 6 (35%), influenza 1 (5.9%), lung infection 1 (5.9%), wound infection (staph) 1 (5.9%).<br>Extension phase: nasopharyngitis 7 (41%), diarrhoea 7 (41%), pyrexia 6 (35%), vomiting 7 (41%), pneumonia (2 (11.8%), bronchitis 1 (5.9%).				
**Malignancy**	Not reported.				
**ILD**	Not reported.				
**Drug reaction**	Not reported.				
**SAE**	Open label trial: CAPS 1 (5.9%), cryptorchidism 1 (5.9%), diarrhoea 1 (5.9%), vomiting 1 (5.9%), influenza 1 (5.9%), lung infection 1 (5.9%), wound infection (staph) 1 (5.9%), femur fracture 1 (5.9%)<br>Extension phase:: vomiting 1 (5.9%), conductive deafness 1 (5.9%), abdominal pain 1 (5.9%), papillitis 1 (5.9%), pneumonia 2 (11.8%), bronchitis 1 (5.9%), meningitis aseptic 1 (5.9%), limb injury 1 (5.9%), hematoma 1 (5.9%).				
**Discontinuation**	There were no discontinuations due to AEs or SAEs during the entire study.				
**Death**	There were no deaths due to AEs or SAEs during the entire study.				
**Additional notes**	Nil.				
**Risk of bias (<6 high, 6-11 moderate, >11 low risk of bias)**					
**Aim**	2	**Consecutive**	2	**Prospective**	2
**Endpoints**	2	**Assessment**	0	**Follow-up**	1
**Loss to follow-up**	1	**Calculation**	0	**Total**	10 (moderate)
AE adverse effect, CAN canakinumab, CAPS Cryopyrin Associated Periodic Syndrome, ILD interstitial lung disease, PY patient year, SAE serious adverse event					

**Study**	Brunner 2020		
**Primary reference**	Brunner HI, Quartier P, Alexeeva E, Constantin T, Kone-Paut I, Marzan K, et al. Efficacy and Safety of Canakinumab in Patients With Systemic Juvenile Idiopathic Arthritis With and Without Fever at Baseline: Results From an Open-Label, Active-Treatment Extension Study. Arthritis rheumatol. 2020;72(12):2147-58.		
**Associated references**	Ruperto 2018	**Study identifier**	NCT00891046<br>NCT00889863<br>NCT00886769

Study characteristics					
**Date of study**	Feb 2012 to Dec 2014	**Design**	LTE	**Condition**	SJIA
**Medication**	CAN (123)	**Number of patients (IL-1 treated)**	123 (123)	**Age**	Fever: 10.5 years (IQR 6.6-13.4) W/O fever: 8.2 years (IQR 4.6-12.2)
**Follow-up**	Median 1.8 y, total 183.69 PY	**Country**	15 countries at 37 centers that were members of PRINTO and PRCSG	**Sex**	Fever: 26 M / 44 F W/O fever: 21 M / 31 F
**Author's conclusions**	Canakinumab provided rapid and sustained improvement of active systemic JIA irrespective of the presence of fever at treatment initiation.				
**Outcomes**					
**Infection**	Fever: infections and infestations 10 (14.3%, 14.8/100PY), pneumonia 3 (4.3%, 3/100PY), gastroenteritis 2 (2.9%, 2/100PY), bronchopneumonia 1(1.4%, 1/100PY), cytomegalovirus 1 (1.4%, 1/100PY), escherichia urinary tract infection 1 (1.4%, 1/100PY), herpes zoster 1 (1.4%, 1/100PY), influenza 1 (1.4%, 1/100PY), otitis media acute 1 (1.4%, 1/100PY), salmonella sepsis 1 (1.4%, 1/100PY), scarlet fever 1 (1.4%, 1/100PY), staphylococcal sepsis 1 (1.4%, 1/100PY), varicella 1 (1.4%, 1/100PY).<br>W/O fever: infections and infestations 3 (5.8%, 8.6/100PY), abscess neck 1 (1.9%, 1.2/100PY), cellulitis 1 (1.9%, 1.2/100PY), furuncle 1 (1.9%, 1.2/100PY), infected bites 1 (1.9%, 1.2/100PY), lymph node abscess 1 (1.9%, 1.2/100PY), lymphangitis 1 (1.9%, 1.2/100PY), sepsis 1 (1.9%, 1.2/100PY).				
**Malignancy**	1 case of malignancy (anaplastic large cell lymphoma) in a patient with fever, diagnosed after 113 days of canakinumab treatment and considered unlikely to be related to canakinumab. Determined that the initial articular symptoms and fever were kind of paraneoplastic manifestations resulting in an incorrect diagnosis of SJIA.				
**ILD**	Not reported.				
**Drug reaction**	Not reported.				
**SAE**	The exposure-adjusted incidence rate of SAEs over the duration of the study was 56 per 100 patient-years. Higher incidence rates of SAEs (95.74 per 100 patient-years) were observed during the initial 6 months of the study, and then these rates decreased to 59.05 per 100 person-years by month 24.				
**Discontinuation**	Discontinuation due to adverse effect fever group 10/70 (14.3%) and W/O fever 4/52 (7.7%).				
**Death**	Not reported.				
**Additional notes**	Nil.				
**Risk of bias (<6 high, 6-11 moderate, >11 low risk of bias)**					
**Aim**	2	**Consecutive**	1	**Prospective**	2
**Endpoints**	2	**Assessment**	0	**Follow-up**	0
**Loss to follow-up**	0	**Calculation**	0	**Total**	7 (moderate)

CAN canakinumab, ILD interstitial lung disease, IQR interquartile range, LTE long term extension, PRCSG Pediatric Rheumatology Collaborative Study Group, PRINTO Paediatric Rheumatology International Trials Organisation, PY patient-years, SAE serious adverse event, SJIA systemic onset juvenile idiopathic arthritis, W/O without

**Study**	Cabrera 2019				
**Primary reference**	Cabrera N, Lega JC, Kassai B, Wouters C, Kondi A, Cannizzaro E, et al. Safety of biological agents in paediatric rheumatic diseases: A real-life multicenter retrospective study using the JIRcohorte database. Joint Bone Spine. 2019;86(3):343-50.				
**Associated references**	Dumaine 2020<br>Kone-Paut 2024		**Study identifier**	NTC02377245	
**Study characteristics**					
**Date of study**	Up to Aug 2014	**Design**	Registry	**Condition**	Many JIA and non-JIA
**Medication**	ANA (85), CAN (75)	**Number of patients (IL-1 treated)**	813 (160)	**Age**	9.4 years (SD 3.6) for entire cohort
**Follow-up**	ANA 207 PY (mean 2.4)<br>CAN 243 PY (mean 3.2)	**Country**	Switzerland, France, Morocco, Belgium.	**Sex**	295 M / 518 F for entire cohort
**Author's conclusions**	This study suggests an overall an acceptable safety of biologic agents in children with inflammatory rheumatic diseases treated with biological agents. However, the concomitant prescription of immunosuppressive drugs with biological agents represents a substantial risk of adverse events.				
**Outcomes**					
**Infection**	ANA: all infections 11 (5.3/100PY), bacteria 4 (1.9/100PY), virus 4 (1.9/100PY), EBV infection 1 (0.5/100PY), other virus 3 (1.4/100PY), other infection 3 (1.4/100PY).<br>CAN: all infections 28 (11.5/100PY), bacteria 5 (2.1/100PY), virus 12 (4.9/100PY), VZV infection 1 (0.4/100PY), another virus 10 (4.1/100PY), other infections 11 (4.5/100PY), sepsis 1 (0.4/100PY).				
**Malignancy**	Not reported.				
**ILD**	Not reported.				
**Drug reaction**	Not reported.				
**SAE**	ANA: 10 (4.8/100PY).<br>CAN: 12 (4.9/100PY).				
**Discontinuation**	Not reported.				
**Death**	No very severe adverse events for ANA or CAN.				
**Additional notes**	One demyelinating lesion appeared concomitantly with canakinumab (incidence rate 0.4/100PY).				
**Risk of bias (<6 high, 6-11 moderate, >11 low risk of bias)**					

**Aim**	2	**Consecutive**	2	**Prospective**	0
**Endpoints**	2	**Assessment**	0	**Follow-up**	1
**Loss to follow-up**	0	**Calculation**	0	**Total**	7 (moderate)
ANA anakinra, CAN canakinumab, EBV Epstein-Barr virus, ILD interstitial lung disease, JIA juvenile idiopathic arthritis, PY patient-year, SAE serious adverse event, VZV Varicella zoster virus					

**Study**	Cakan 2020				
**Primary reference**	Cakan M, Karadag SG, Ayaz NA. Canakinumab in colchicine resistant familial Mediterranean fever and other pediatric rheumatic diseases. Turk J Pediatr. 2020;62(2):167-74.				
**Associated references**	Nil		**Study identifier**	Nil	
**Study characteristics**					
**Date of study**	Dec 2012 to Jan 2017	**Design**	Retrospective cohort study	**Condition**	FMF (19), MKD (3), CAPS (3), SJIA (2), pericarditis (1), pyoderma gangrenosum (1)
**Medication**	CAN (29)	**Number of patients (IL-1 treated)**	29 (29)	**Age**	Mean FMF 5.3 years, MKD 5.5 years, CAPS 6.2 years
**Follow-up**	Mean 21.8 months (range 6-54)	**Country**	Türkiye	**Sex**	FMF 3 male / 16 female
**Author's conclusions**	Overall efficacy of canakinumab was 93.1% in this study. No major adverse event was observed under canakinumab treatment. Canakinumab seems to be effective and safe in children with rheumatic diseases.				
**Outcomes**					
**Infection**	One patient had impetigo and five patients had upper respiratory tract infections.				
**Malignancy**	Not reported.				
**ILD**	Not reported.				
**Drug reaction**	All canakinumab injections were well tolerated with no injection site reactions.				
**SAE**	No major adverse event was observed under canakinumab treatment.				
**Discontinuation**	No discontinuation due to adverse effects (1 discontinued due to control of symptoms, 2 discontinued due to persistence of clinical features).				
**Death**	Not reported.				

**Additional notes**	Nil.				
**Risk of bias (<6 high, 6-11 moderate, >11 low risk of bias)**					
**Aim**	2	**Consecutive**	2	**Prospective**	0
**Endpoints**	1	**Assessment**	0	**Follow-up**	0
**Loss to follow-up**	0	**Calculation**	0	**Total**	5 (high)
ANA anakinra, CAN canakinumab, CAPS cryopyrin associated periodic syndrome, FMF familial Mediterranean fever, ILD interstitial lung disease, MKD mevalonate kinase deficiency, SAE serious adverse event, SJIA systemic juvenile idiopathic arthritis					

**Study**	Coskuner 2023				
**Primary reference**	Coskuner T, Caglayan S, Akgun O, Torun R, Yayla ENS, Bagrul IL, et al. The safety of canakinumab in systemic juvenile idiopathic arthritis and autoinflammatory diseases in pediatric patients: a multicenter study. Expert Opin Biol Ther. 2023;23(12):1299-306.				
**Associated references**	Nil		**Study identifier**	Nil	
**Study characteristics**					
**Date of study**	Jun 2016 to Jun 2022	**Design**	Retrospective cohort study	**Condition**	SJIA (55), FMF (209), CAPS (29), MKD (26), TRAPS (9), Majeed (2), NLRP12AID (2), NAIAD (1), Blau (1), MONA (1)
**Medication**	CAN (335)	**Number of patients (IL-1 treated)**	335 (335)	**Age**	Median 8 years (IQR 4-13)
**Follow-up**	Median 23 months (IQR 10-39)	**Country**	Türkiye	**Sex**	158 M / 177 F
**Author's conclusions**	Real-life data from a large cohort of patients suggests that canakinumab is as safe as claimed in clinical trials.				
**Outcomes**					
**Infection**	Renal abscess 1 (0.3%), upper respiratory tract infection 274 (81.8%), COVID-19 infection 42 (12.6%), latent tuberculosis 6 (1.8%), pneumonia 14 (4.1), herpes zoster 1 (0.6%), urinary tract infection 11 (3.3%), acute gastroenteritis 10 (3%), skin infection 8 (2.4%), lymphadenitis 2 (0.6%), fungal infection 2 (0.6%), herpes simplex 3 (0.9%), Epstein-Barr virus 1 (0.3%).<br>In nine patients (2.6%), canakinumab treatment was affected due to infections. While canakinumab treatment was interrupted and restarted in six patients, it was discontinued in three patients. Due to an infection, 19 patients (5.6%) were hospitalized. The median length of hospital stay was 5 (IQR: 3–10) days.				
**Malignancy**	1 (0.3%). A patient with FMF was diagnosed with AML in the 26th month of CAN treatment.				

**ILD**	Interstitial lung disease was not observed in any patient.				
**Drug reaction**	Anaphylaxis or anaphylactoid reactions were not observed in any patient.				
**SAE**	10 events in 8 patients. 4 MAS, 2 Crohn-like disease, 1 malignancy (AML), 1 liver failure, 1 renal abscess, 1 acute pancreatitis.				
**Discontinuation**	Discontinuation in 12/335 (3.6%) (2 Crohn-like disease, 3 infection, 2 MAS, 1 malignancy (AML), 1 hepatic failure, 1 recurrent pancreatitis).				
**Death**	No death was observed in any patient.				
**Additional notes**	Nil.				
**Risk of bias (<6 high, 6-11 moderate, >11 low risk of bias)**					
**Aim**	2	**Consecutive**	1	**Prospective**	0
**Endpoints**	2	**Assessment**	0	**Follow-up**	0
**Loss to follow-up**	0	**Calculation**	0	**Total**	5 (high)
AML acute myeloid leukaemia, CAN canakinumab, CAPS cryopyrin associated periodic syndrome, FMF familial Mediterranean fever, ILD interstitial lung disease, IQR interquartile range, MAS macrophage activation syndrome, MKD mevalonate kinase deficiency, MONA multicentric osteolysis nodulosis and arthropathy, NIAID NLRP1- associated autoinflammation with arthritis and dyskeratosis, NLRP12AID nucleotide-binding leucine-rich repeat-containing receptor 12 autoinflammatory disease, SAE serious adverse event, SJIA systemic juvenile idiopathic arthritis, TRAPS TNF receptor-associated periodic fever syndrome					

**Study**	Demir 2022				
**Primary reference**	Demir F, Gurler E, Sozeri B. Efficacy of anakinra treatment in pediatric rheumatic diseases: Our single-center experience. Arch Rheumatol. 2022;37(3):435-43.				
**Associated references**	Nil		**Study identifier**	Nil	
**Study characteristics**					
**Date of study**	Jul 2016 to Jul 2020	**Design**	Retrospective cohort study	**Condition**	SJIA 11, MVK 6, CAPS 5, FMF 5, pericarditis 4, NLRP12AID, undefined AID 1
**Medication**	ANA (33)	**Number of patients (IL-1 treated)**	33 (33)	**Age**	Median 6 years (IQR 5-13.5)
**Follow-up**	Median 14 months (IQR 3.7-28)	**Country**	Türkiye	**Sex**	15 M / 18 F
**Author's conclusions**	Anakinra appears to be a promising treatment alternative owing to its rapid effect as a result of its short half-life in autoinflammatory conditions. While short-term therapy seems to be sufficient for the sJIA				

	complicated by MAS, the patients with systemic autoinflammatory diseases maintenance a more anakinra-dependent course.				
**Outcomes**					
**Infection**	Not reported.				
**Malignancy**	Not reported.				
**ILD**	Not reported.				
**Drug reaction**	Local reactions 2 (6.1%).				
**SAE**	Not reported.				
**Discontinuation**	Discontinuation due to local reactions 2 (6.1%).				
**Death**	Not reported.				
**Additional notes**	Nil.				
**Risk of bias (<6 high, 6-11 moderate, >11 low risk of bias)**					
**Aim**	1	**Consecutive**	1	**Prospective**	0
**Endpoints**	2	**Assessment**	0	**Follow-up**	0
**Loss to follow-up**	0	**Calculation**	0	**Total**	4 (high)
AID autoinflammatory disease, ANA anakinra, ILD interstitial lung disease, IQR interquartile range, MAS macrophage activation syndrome, NLRP12AID nucleotide-binding leucine-rich repeat-containing receptor 12 autoinflammatory disease, SAE serious adverse event					

**Study**	Dumaine 2020				
**Primary reference**	Dumaine C, Bekkar S, Belot A, Cabrera N, Malik S, von Scheven A, et al. Infectious adverse events in children with Juvenile Idiopathic Arthritis treated with Biological Agents in a real-life setting: Data from the JIRcohorte. Joint Bone Spine. 2020;87(1):49-55.				
**Associated references**	Cabrera 2019<br>Kone-Paut 2024		**Study identifier**	NTC02377245	
**Study characteristics**					
**Date of study**	Jan 2001 to Aug 2015	**Design**	Registry	**Condition**	JIA

**Medication**	ANA (72)<br>CAN (32)	**Number of patients (IL-1 treated)**	677 (104)	**Age**	7.8 years (3.8–11.9 SD)
**Follow-up**	ANA 180 PY (mean 2.5)<br>CAN 74.6 PY (mean 2.3)	**Country**	Multiple	**Sex**	233 M / 444 F
**Author's conclusions**	Infectious complications with biologics occurring in children treated for JIA are rare, and in most of the cases have a mild or moderate severity, affecting mainly the upper respiratory tract or the ENT.				
**Outcomes**					
**Infection**	19 infectious adverse events (18.3/100PY).				
**Malignancy**	Not reported.				
**ILD**	Not reported.				
**Drug reaction**	Not reported.				
**SAE**	3 SAE (15.8/100PY).				
**Discontinuation**	Not reported.				
**Death**	Not reported.				
**Additional notes**	Nil.				
**Risk of bias (<6 high, 6-11 moderate, >11 low risk of bias)**					
**Aim**	2	**Consecutive**	2	**Prospective**	1
**Endpoints**	2	**Assessment**	0	**Follow-up**	1
**Loss to follow-up**	1	**Calculation**	0	**Total**	9 (moderate)
ILD interstitial lung disease, JIA juvenile idiopathic arthritis, PY patient-year, SAE serious adverse event					

**Study**	Fingerhutova 2022

**Primary reference**	Fingerhutova S, Jancova E, Dolezalova P. Anakinra in Paediatric Rheumatology and Periodic Fever Clinics: Is the Higher Dose Safe? Front Pediatr. 2022;10:823847.				
**Associated references**	Nil		**Study identifier**	Nil	
**Study characteristics**					
**Date of study**	Jan 2015 to May 2021	**Design**	Retrospective cohort study	**Condition**	SJIA (19), CAPS (10), MKD (6), SURF (6), PIMS-TS (2), other AID (2), MAS (2)
**Medication**	ANA (47)	**Number of patients (IL-1 treated)**	47 (47)	**Age**	Children 8.9 years (n=32, SD 5.7)<br>Adults 33.8 years (n=15, SD 12)
**Follow-up**	1.9 years (SD 1.9) treatment 2.7 years follow up after initiation	**Country**	Czech Republic	**Sex**	20 M / F 27
**Author's conclusions**	Our results support early use of anakinra in the individually tailored dosing. In patients with hyperinflammation, anakinra may be lifesaving and may even allow for corticosteroid avoidance. Further studies are needed in order to set up generally accepted response parameters and define condition-specific optimal dosing regimen.				
**Outcomes**					
**Infection**	1 moderately severe COVID.				
**Malignancy**	Not reported.				
**ILD**	Eosinophilia was documented in 10/47 (21.3%) patients. It developed usually within the first 4–6 weeks, and in cases with mild elevation (0.52.0 × 109/L), it disappeared spontaneously within 2 months. Higher eosinophilia with prolonged duration in 2 SJIA patients (3.6 and 2.3 × 109/L) was associated with severe disease with lung involvement in one of them.				
**Drug reaction**	Injection site reaction in 27.7% of patients (seven adults (46.7%) and six children (18.8%)). It disappeared within 1 month in all patients.				
**SAE**	5/47 (10.6%) patients experienced at least one SAE. A 2-year old infant with SJIA developed epilepsy while on anakinra for 5 months. One patient with SJIA was hospitalized due to a moderately severe COVID with transient need of increased anakinra dose. An adult patient (57 years old) with CAPS was hospitalized for total hip replacement surgery due to osteoarthritis. Another polymorbid adult patient with MWS died at 56 years of age after 2 months of anakinra for a relapse of pre-existing pancreatitis and acute pulmonary embolism. The girl with Blau syndrome had an episode of neurosarcoidosis requiring hospitalization and high-dose intravenous steroids. All SAE appeared in patients who had standard doses of anakinra.				
**Discontinuation**	Not reported.				
**Death**	One polymorbid adult patient with MWS died at 56 years of age after 2 months of anakinra for a relapse of pre-existing pancreatitis and acute pulmonary embolism.				
**Additional notes**	Nil.				
**Risk of bias (<6 high, 6-11 moderate, >11 low risk of bias)**					

Aim	2	Consecutive	2	Prospective	0
Endpoints	1	Assessment	0	Follow-up	1
Loss to follow-up	1	Calculation	0	Total	7 (moderate)
AID autoinflammatory disease, CAPS cryopyrin associated periodic syndromes, ILD interstitial lung disease, MAS macrophage activation syndrome, MKD mevalonate kinase deficiency, MWS Muckle-Wells syndrome, SAE serious adverse event, SD standard deviation, SJIA systemic juvenile idiopathic arthritis, SURF syndrome of undifferentiated recurrent fever					

**Study**	Foley 2023				
**Primary reference**	Foley CM, McKenna D, Gallagher K, McLellan K, Alkhdher H, Lacassagne S, et al. Systemic juvenile idiopathic arthritis: The Great Ormond Street Hospital experience (2005-2021). Front Pediatr. 2023;11:1218312.				
**Associated references**	Nil		**Study identifier**	Nil	
**Study characteristics**					
**Date of study**	Oct 2005- Oct 2021	**Design**	Retrospective cohort study	**Condition**	SJIA
**Medication**	ANA (21)	**Number of patients (IL-1 treated)**	76 (21)	**Age**	Median age 4.5 years (range 0.6–14.1)
**Follow-up**	Median 4.7 years (range 0.2–16.0)	**Country**	United Kingdom	**Sex**	M 36 / F 40
**Author's conclusions**	Based on an ever-increasing evidence base for the earlier use of bDMARD in sJIA and our experience of the largest UK single-centre case series described to date, we now propose a new therapeutic pathway for children diagnosed with sJIA in the UK based on early use of bDMARDs. Reappraisal of the current National Health Service commissioning pathway for sJIA is now urgently required.				
**Outcomes**					
**Infection**	Not reported.				
**Malignancy**	Not reported.				
**ILD**	One patient (1%) had an abnormal high resolution CT (HRCT) thorax at diagnosis. This 14.1-year-old female presented with MAS with patchy ground glass changes of the lungs bi-basally. She was treated with bDMARD (anti-IL1, anakinra) within 3 months of diagnosis, and at last review, 2.7 years since diagnosis, was in remission (CID off all treatment). Four months after data collection was completed, we observed a case of pulmonary alveolar proteinosis (PAP) in a 17-month-old girl with sJIA and smouldering MAS (Supplementary Figure S2). She presented with treatment refractory sJIA complicated by MAS at the age of 8 months. She remained glucocorticoid dependent throughout her disease course and received anti-IL6 and anti-IL1 blockade, in addition to cyclosporine, before progressing to etoposide as a bridge to allogeneic-HSCT. Neither patient had any documented idiosyncratic reaction following bDMARD; and HLA testing was not performed in any of the patients.				
**Drug reaction**	Not reported.				

**SAE**	Not reported.				
**Discontinuation**	Not reported.				
**Death**	0.				
**Additional notes**	MAS 35/76 (46%).				
**Risk of bias (<6 high, 6-11 moderate, >11 low risk of bias)**					
**Aim**	1	**Consecutive**	2	**Prospective**	0
**Endpoints**	2	**Assessment**	0	**Follow-up**	2
**Loss to follow-up**	0	**Calculation**	0	**Total**	7 (moderate)
ANA anakinra, bDMARD biologic disease modifying anti-rheumatic disease, CID clinically inactive disease, HLA human leukocyte antigen, HRCT high resolution computerised tomography, HSCT hematopoietic stem cell transplantation, ILD interstitial lung disease, MAS macrophage activation syndrome, PAP pulmonary alveolar proteinosis, SAE serious adverse event, SJIA systemic onset juvenile idiopathic arthritis, UK United Kingdom					

**Study**	Garg 2019				
**Primary reference**	Garg S, Wynne K, Omoyinmi E, Eleftheriou D, Brogan P. Efficacy and safety of anakinra for undifferentiated autoinflammatory diseases in children: a retrospective case review. Rheumatol Adv Pract. 2019;3(1):rkz004.				
**Associated references**	Nil		**Study identifier**	Nil	
**Study characteristics**					
**Date of study**	Jan 2009 to Jan 2018	**Design**	Retrospective cohort study	**Condition**	uAID
**Medication**	ANA (22)	**Number of patients (IL-1 treated)**	22 (22)	**Age**	Mean 7.1 years (range 0.1 - 14.1)
**Follow-up**	Median 19.6 months	**Country**	United Kingdom	**Sex**	8 M / 14 F
**Author's conclusions**	Retrospectively, 72% of children with uAID responded well to anakinra, with 36% achieving full clinical and serological remission within 3 months. This suggests that empirical trials of IL-1 blockade might be warranted in children with uAID. Clear stopping criteria based on predefined parameters should be considered, because non-responders required alternative therapies, facilitated by a definitive molecular diagnosis where possible.				
**Outcomes**					
**Infection**	8 (36.4%) patients had infection.				
**Malignancy**	Not reported.				

**ILD**	Not reported.
**Drug reaction**	5 (22.7%) had injection site reactions.
**SAE**	12 serious adverse events in 10 (45.5%) patients. 3 painful injection site reactions requiring presentation to their local hospital (none required specific intervention or cessation), 3 neutropenia requiring presentation to hospital, 1 presumed viral infection with disease flare, 1 urinary tract infection and concomitant varicella zoster virus infection, 1 presumed viral upper respiratory tract infection, 1 suspected sepsis (no organism identified) and disease flare, 1 orbital cellulitis, 1 death.
**Discontinuation**	Discontinuation due to intolerance in 1 (4.5%).
**Death**	3 (13.6%) died (1 (4.5%) while on anakinra). 1 deceased due to MAS, 1 deceased due to multiorgan failure fro CANDLE syndrome (not on anakinra at the time of death), 1 deceased from multiorgan failure from PFIT syndrome (not on anakinra at the time of death).
**Additional notes**	Nil.

**Risk of bias (<6 high, 6-11 moderate, >11 low risk of bias)**					
**Aim**	2	**Consecutive**	2	**Prospective**	0
**Endpoints**	2	**Assessment**	0	**Follow-up**	0
**Loss to follow-up**	2	**Calculation**	0	**Total**	8 (moderate)

ANA anakinra, CANDLE chronic atypical neutrophilic dermatosis with lipodystrophy and elevated temperature, ILD interstitial lung disease, PFIT periodic fever immunodeficiency and thrombocytopenia, SAE serious adverse event, uAID undifferentiated autoinflammatory disease

**Study**	Giancane 2022				
**Primary reference**	Giancane G, Papa R, Vastert S, Bagnasco F, Swart JF, Quartier P, et al. Anakinra in Patients With Systemic Juvenile Idiopathic Arthritis: Long-term Safety From the Pharmachild Registry. J Rheumatol. 2022;49(4):398-407.				
**Associated references**	Nil		**Study identifier**	NCT01399281<br>NCT03932344	
**Study characteristics**					
**Date of study**	Dec 2011 to Sep 2018	**Design**	Registry	**Condition**	SJIA
**Medication**	ANA (306)	**Number of patients (IL-1 treated)**	306 (306)	**Age**	Median 8.0 years
**Follow-up**	Mean 17.0 months (SD 21.1)	**Country**	15 countries from Europe (97.7%) and Asia (2.3%)	**Sex**	152 M / 154 F
**Author's conclusions**	The results of the present study confirm the long-term safety profile of anakinra in patients with sJIA and demonstrate an overall decreasing incidence of AEs over time.				
**Outcomes**					

**Infection**	52 infectious adverse effects (10.2/100PY). Respiratory tract infections accounted for 53.8% (28/52). 3 cases of varicella, and 1 case of herpes zoster were also identified.
**Malignancy**	No malignancies occurred during anakinra exposure.
**ILD**	1 patient reported an event of interstitial lung disease (ILD) after receiving treatment with anakinra for > 24 months.
**Drug reaction**	23 administration site conditions (4.5/100 PY) including 16 injection site reactions.
**SAE**	56 SAEs (11.0/100PY). 13 infections and infestations were most common (2.6/100PY), 11 immune system disorders (all describing MAS) (2.2/100PY), 9 Injury / poisoning / procedural complications (1.8/100PY). The remaining SAEs had a rate of < 1.0/100PY.
**Discontinuation**	In total, there were 268 discontinuations with 281 reasons recorded. The most frequent reason for anakinra discontinuation was inefficacy (43.1%), followed by remission (30.6%). AEs caused 10.0% of discontinuations: in 8.2% of the cases because of events of moderate intensity, and mild in the remaining cases. Intolerance was given as the reason for discontinuation in 5.0% of the cases. Discontinuations as a result of AEs and intolerance were more frequently reported during the first 6 months of therapy.
**Death**	No SAEs leading to death occurred during anakinra exposure. Outside of anakinra exposure, 3 patients died 0.5, 3, and 5 years after anakinra discontinuation.
**Additional notes**	The overall incidence of AEs decreased over time, with the highest IRs during the first 6 months of anakinra treatment.

**Risk of bias (<6 high, 6-11 moderate, >11 low risk of bias)**					
**Aim**	2	**Consecutive**	1	**Prospective**	1
**Endpoints**	2	**Assessment**	0	**Follow-up**	0
**Loss to follow-up**	0	**Calculation**	0	**Total**	6 (moderate)

ANA anakinra, ILD interstitial lung disease, MAS macrophage activation syndrome, PY patient-year, SAE serious adverse event, SD standard deviation, SJIA systemic onset juvenile idiopathic arthritis

**Study**	Gulez 2020				
**Primary reference**	Gulez N, Makay B, Sozeri B. Long-term effectiveness and safety of canakinumab in pediatric familial Mediterranean fever patients. Mod Rheumatol. 2020;30(1):166-71.				
**Associated references**	Nil	**Study identifier**	Nil		
**Study characteristics**					
**Date of study**	2012 to 2017	**Design**	Retrospective cohort study	**Condition**	FMF
**Medication**	CAN (15)	**Number of patients (IL-1 treated)**	15 (15)	**Age**	16.5 years (range 8 to 19)
**Follow-up**	23.9 months (12 to 58)	**Country**	Türkiye	**Sex**	8 M / 7 F

**Author's conclusions**	To the best of our knowledge, this is the longest outcome study about canakinumab use in paediatric FMF patients. This study suggested that canakinumab is safe and effective in children with FMF in the long term.				
**Outcomes**					
**Infection**	2 mild lower urinary tract infections, 2 tooth abscesses, 1 bronchopneumonia requiring short-term hospitalization. There were no opportunistic infections.<br>Four patients had a TST>5mm, so they were given a 6-month course of isoniazid prophylaxis. Their QuantiFERONTB Gold (Mycobacterium tuberculosis-specific interferon tests were negative. Their mean duration of canakinumab use was 33±20 months. None of these four patients developed tuberculosis in the followup.				
**Malignancy**	No malignancies.				
**ILD**	Not reported.				
**Drug reaction**	There were no injection site reactions.				
**SAE**	There were no serious adverse events.				
**Discontinuation**	Not reported.				
**Death**	No deaths.				
**Additional notes**	Nil.				
**Risk of bias (<6 high, 6-11 moderate, >11 low risk of bias)**					
**Aim**	2	**Consecutive**	2	**Prospective**	0
**Endpoints**	1	**Assessment**	0	**Follow-up**	0
**Loss to follow-up**	1	**Calculation**	0	**Total**	6 (moderate)
CAN canakinumab, FMF familial mediterranean fever, ILD interstitial lung disease, SAE serious adverse event, TST tuberculin skin test					

**Study**	Horneff 2017		
**Primary reference**	Horneff G, Schulz AC, Klotsche J, Hospach A, Minden K, Foeldvari I, et al. Experience with etanercept, tocilizumab and interleukin-1 inhibitors in systemic onset juvenile idiopathic arthritis patients from the BIKER registry. Arthritis Res Ther. 2017;19(1):256.		
**Associated references**	Atemnkeng Ntam 2021<br>Klein 2020<br>Thiele 2021	**Study identifier**	Nil
**Study characteristics**			

**Date of study**	2000 to 2015	**Design**	Registry	**Condition**	SJIA
**Medication**	ANA (38)<br>CAN (22)	**Number of patients (IL-1 treated)**	243 (60)	**Age**	Median age 9.2 years (SD 4.8) in IL-1 group
**Follow-up**	24 months	**Country**	Germany	**Sex**	M 32 / F 28
**Author's conclusions**	A large proportion of patients gained significant response to treatment especially with IL-1is. After 6 months on treatment, JADAS remission was reached by up to half of patients while up to two thirds reached JADAS minimal disease activity.				
**Outcomes**					
**Infection**	6 serious infections IL-1i cohort (1 patient with pneumonia, 1 with tonsillitis, 1 with enteritis and 1 with the common cold and 2 with bronchitis).				
**Malignancy**	None in the IL1 cohort.				
**ILD**	Not reported.				
**Drug reaction**	Hypersensitivity 2/60 (0.02/PY).				
**SAE**	17/60 (0.15/PY).				
**Discontinuation**	1 due to intolerance.				
**Death**	None in IL1 cohort.				
**Additional notes**	3 cases of MAS in IL1 cohort.				
**Risk of bias (<6 high, 6-11 moderate, >11 low risk of bias)**					
**Aim**	1	**Consecutive**	2	**Prospective**	1
**Endpoints**	2	**Assessment**	0	**Follow-up**	1
**Loss to follow-up**	0	**Calculation**	0	**Total**	7 (moderate)
ANA anakinra, CAN canakinumab, ILD interstitial lung disease, MAS macrophage activation syndrome, PY patient year, SAE serious adverse event, SD standard deviation					

**Study**	Ilowite 2009
**Primary reference**	Ilowite N, Porras O, Reiff A, Rudge S, Punaro M, Martin A, et al. Anakinra in the treatment of polyarticular-course juvenile rheumatoid arthritis: safety and preliminary efficacy results of a randomized multicenter study. Clin Rheumatol. 2009;28(2):129-37.

**Associated references**	Nil		**Study identifier**	NCT00037648	
**Study characteristics**					
**Date of study**	July 2000 to Feb 2004	**Design**	RCT	**Condition**	Polyarticular JIA
**Medication**	ANA (86)	**Number of patients (IL-1 treated)**	86 (86)	**Age**	Mean 12 years (range 3-17)
**Follow-up**	28 weeks plus 12 months extension (n=44)	**Country**	Multiple	**Sex**	M 23 / F 63
**Author's conclusions**	These results indicate that anakinra 1 mg/kg once daily (≤100 mg/day) is safe and well tolerated in patients with JRA.				
**Outcomes**					
**Infection**	41% in open label, 36% on drug in blinded phase, 32% on placebo in blinded phase, 36% in the extension phase (most common, URTI, fever and influenza like symptoms).				
**Malignancy**	Not reported.				
**ILD**	Not reported.				
**Drug reaction**	64/86 (74%) had application site reactions in the open label stage; ecchymosis 12 (14%), edema 9 (11%), inflammation 8 (9%), pain 29 (33%), pruritus 26 (30%), rash 14 (16%) and reaction 10 (12%).				
**SAE**	6 SAEs. 3 in open label not thought to be related to study drug. 3 in extension (nephrosis, hepatitis, viral infection not believed to be related to the study drug).				
**Discontinuation**	At least one patient discontinued due to injection site reactions. Most patients discontinued for reasons other than adverse events.				
**Death**	Nil.				
**Additional notes**	Nil.				
**Risk of bias (<6 high, 6-11 moderate, >11 low risk of bias)**					
**Aim**	2	**Consecutive**	1	**Prospective**	2
**Endpoints**	2	**Assessment**	1	**Follow-up**	0
**Loss to follow-up**	1	**Calculation**	1	**Total**	10 (moderate)
ANA anakinra, ILD interstitial lung disease, JIA juvenile idiopathic arthritis, RCT randomised controlled trial, SAE serious adverse event					

**Study**	Ilowite 2014

**Primary reference**	Ilowite NT, Prather K, Lokhnygina Y, Schanberg LE, Elder M, Milojevic D, et al. Randomized, double-blind, placebo-controlled trial of the efficacy and safety of rilonacept in the treatment of systemic juvenile idiopathic arthritis. Arthritis rheumatol. 2014;66(9):2570-9.				
**Associated references**	Nil		**Study identifier**	RAPPORT trial	
**Study characteristics**					
**Date of study**	Nov 2008 to May 2012	**Design**	RCT and LTE	**Condition**	SJIA
**Medication**	RIL (71)	**Number of patients (IL-1 treated)**	71 (71)	**Age**	Mean 9.5 years (SD 4.6) rilonacept 10.5 years (SD 4.4) placebo
**Follow-up**	40 patients treated to 21 months	**Country**	United States of America	**Sex**	M 46 / F 25
**Author's conclusions**	Rilonacept was generally well tolerated and demonstrated efficacy in active systemic JIA.				
**Outcomes**					
**Infection**	Infection events RIL open label 0.7 PPY, placebo open label 0.8 PPY, RIL treatment phase 1.9 PPY, placebo treatment phase 2.7 PPY, RIL extension phase 1.0 PPY.				
**Malignancy**	Not reported.				
**ILD**	Not reported.				
**Drug reaction**	Not reported.				
**SAE**	14 total (12 in rilonacept arm, 2 placebo). 4 juvenile arthritis (2 RIL, 2 placebo), 1 abnormal liver function test (1 RIL), 1 pyrexia (1 RIL), 1 varicella (1 RIL), 1 viral upper respiratory tract infection (1 RIL), 1 pharyngitis streptococcal infection (1 RIL), 1 mental status changes (1 RIL), 1 salmonella (1 RIL), 1 haemophagocytic histiocytosis (1 RIL).				
**Discontinuation**	1 discontinuation due to elevated liver transaminase levels.				
**Death**	Nil.				
**Additional notes**	Nil				
**Risk of bias (<6 high, 6-11 moderate, >11 low risk of bias)**					
**Aim**	2	**Consecutive**	2	**Prospective**	2
**Endpoints**	2	**Assessment**	1	**Follow-up**	0
**Loss to follow-up**	2	**Calculation**	1	**Total**	12 (low)

ILD interstitial lung disease, PPY per patient-year, RIL rilonacept, SAE serious adverse event, SD standard deviation, SJIA systemic onset juvenile idiopathic arthritis

**Study**	Iwata 2023				
**Primary reference**	Iwata N, Nishimura K, Hara R, Imagawa T, Shimizu M, Tomiita M, et al. Long-term efficacy and safety of canakinumab in the treatment of systemic juvenile idiopathic arthritis in Japanese patients: Results from an open-label Phase III study. Mod Rheumatol. 2023;33(6):1162-70.				
**Associated references**	Nil		**Study identifier**	NCT02396212	
**Study characteristics**					
**Date of study**	Not reported	**Design**	Single arm active treatment study	**Condition**	SJIA
**Medication**	CAN (19)	**Number of patients (IL-1 treated)**	19 (19)	**Age**	Not reported
**Follow-up**	40.3 PY (mean 2.1)	**Country**	Japan	**Sex**	Not reported
**Author's conclusions**	Canakinumab treatment resulted in a sustained treatment response in sJIA patients over 48 weeks and was associated with CS tapering in majority of patients. No new safety findings were reported.				
**Outcomes**					
**Infection**	All infections and infestations 44 events among 17 (89.5%) patients. SAE infections and infestations 7 events among 5 (26.3%) patients; ; 2 influenza, 1 EBV, 1 gastro, 1 pharyngitis, 1 varicella, 1 viral.				
**Malignancy**	Not reported.				
**ILD**	Not reported.				
**Drug reaction**	Not reported.				
**SAE**	10 patients with at least one SAE (52.6%). 6 musculoskeletal disorders, 5 infections, 3 blood and lymphatic disorders, 2 endocrine disorders, 1 general and administration site conditions, 1 central nervous system disorder, 1 psychiatric disorder.				
**Discontinuation**	None due to adverse events (2 lack of efficacy and 1 worsening of JIA).				
**Death**	Nil.				
**Additional notes**	Nil.				
**Risk of bias (<6 high, 6-11 moderate, >11 low risk of bias)**					
**Aim**	2	**Consecutive**	2	**Prospective**	2

**Endpoints**	2	**Assessment**	0	**Follow-up**	1
**Loss to follow-up**	2	**Calculation**	0	**Total**	11 (moderate)
CAN canakinumab, ILD interstitial lung disease, JIA juvenile idiopathic arthritis, PY patient-year, SAE serious adverse event, SJIA systemic onset juvenile idiopathic arthritis					

**Study**	Jeyaratnam 2022				
**Primary reference**	Jeyaratnam J, Simon A, Calvo I, Constantin T, Shcherbina A, Hofer M, et al. Long-term efficacy and safety of canakinumab in patients with mevalonate kinase deficiency: results from the randomised Phase 3 CLUSTER trial. Rheumatology (Oxford). 2022;61(5):2088-94.				
**Associated references**	Ozen 2020		**Study identifier**	NCT02059291	
**Study characteristics**					
**Date of study**	Not reported	**Design**	RCT LTE	**Condition**	MKD
**Medication**	CAN (74)	**Number of patients (IL-1 treated)**	74 (74)	**Age**	Median 11.5 years (IQR 6-19)
**Follow-up**	Median 507 days	**Country**	33 centres in 13 countries	**Sex**	M 28 / F 46
**Author's conclusions**	Canakinumab proved effective to control disease activity and prevent flares in mevalonate kinase deficiency during the 72-week study period. No new safety concerns were reported.				
**Outcomes**					
**Infection**	Eleven serious infections were reported in nine patients; [pneumonia (n =3), one each: anal abscess, appendicitis, bronchitis, herpesvirus infection, influenza, orchitis, pyelonephritis and tonsillitis.				
**Malignancy**	Not reported.				
**ILD**	Not reported.				
**Drug reaction**	Not reported.				
**SAE**	Twenty-eight serious adverse events were reported in 14 patients. Three of these 28 serious adverse events were MKD flares, although some other serious adverse events might also have been caused by MKD flares.				
**Discontinuation**	Nil.				
**Death**	Nil				
**Additional notes**	Nil.				
**Risk of bias (<6 high, 6-11 moderate, >11 low risk of bias)**					

**Aim**	2	**Consecutive**	1	**Prospective**	2
**Endpoints**	2	**Assessment**	1	**Follow-up**	0
**Loss to follow-up**	2	**Calculation**	0	**Total**	10 (moderate)
ILD interstitial lung disease, IQR interquartile range, MKD mevalonate kinase deficiency, SAE serious adverse event					

**Study**	Jones 2024				
**Primary reference**	Jones OY. Single Center-Based Real-World Experience on Anti-IL 1 Biological Response Modifiers: A Case Series and Literature Review. Children (Basel). 2024;11(9):22.				
**Associated references**	Nil		**Study identifier**	Nil	
**Study characteristics**					
**Date of study**	2012 to 2024	**Design**	Retrospective cohort study	**Condition**	AI (12), SJIA (6), MIS-C (4), KD (4), ARF (3), other
**Medication**	ANA (63)<br>CAN (9)<br>(7 had both)	**Number of patients (IL-1 treated)**	65 (65)	**Age**	AI mean age 7.4<br>SJIA mean age 12<br>MIS-c mean 9.9 years<br>KD mean 4.2 years
**Follow-up**	Not reported	**Country**	United States of America	**Sex**	Various ratios per indication
**Author's conclusions**	Based on our observations and successful outcomes, we advocate for future collaborative efforts to improve access to anti-IL-1 medications to better manage excessive and harmful inflammation in children.				
**Outcomes**					
**Infection**	"No increased infection" from discussion.				
**Malignancy**	Not reported.				
**ILD**	Not reported.				
**Drug reaction**	"No allergic reaction" from discussion.				
**SAE**	"No SAE" from discussion.				
**Discontinuation**	Not reported.				
**Death**	Not reported.				

**Additional notes**	This paper is a descriptive narrative of cases.				
**Risk of bias (<6 high, 6-11 moderate, >11 low risk of bias)**					
**Aim**	1	**Consecutive**	1	**Prospective**	0
**Endpoints**	0	**Assessment**	0	**Follow-up**	0
**Loss to follow-up**	0	**Calculation**	0	**Total**	2 (high)
AI autoinflammatory, ANA anakinra, ARF acute rheumatic fever, ILD interstitial lung disease, KD Kawasaki disease, MIS-C multisystem inflammatory syndrome in children, SAE serious adverse event, SJIA systemic onset juvenile idiopathic arthritis					

**Study**	Kilic Konte 2024				
**Primary reference**	Kilic Konte E, Akay N, Gul U, Ucak K, Derelioglu EI, Gurleyik D, et al. Long-term safety profile and secondary effectiveness of canakinumab in pediatric rheumatic diseases: a single-center experience. Expert Opin Drug Saf. 2024:1-9.				
**Associated references**	Nil		**Study identifier**	NIl	
**Study characteristics**					
**Date of study**	2015 to 2023	**Design**	Retrospective cohort study	**Condition**	FMF, CAPS, TRAPS, CAPS, SJIA, and recurrent pericarditis
**Medication**	CAN (189)	**Number of patients (IL-1 treated)**	189 (189)	**Age**	Median age at diagnosis 5 years (3-8)
**Follow-up**	Median exposure to canakinumab 2.9 years (range 1.5-4.1)	**Country**	Türkiye	**Sex**	M 74 / F 115
**Author's conclusions**	An increase in side effect was not observed with the increasing cumulative doses of canakinumab. Canakinumab demonstrated long-term safety with appropriate indication and monitoring.				
**Outcomes**					
**Infection**	Mild-moderate: 1602 viral URTI (0.76/100PD), 44 urinary tract infections (0.02/100PD), 19 pneumonia (0.009.100PD), 19 latent tuberculosis (0.009/100PD), 9 lymphadenitis (0.004/100PD), 8 bacterial upper respiratory tract infections (0.003/100PD).<br>Severe: 11 pneumonia (0.005/100PD), 2 pyelonephritis (0.001/100PD).				
**Malignancy**	3 patients (0.0015/100PD); 2 lymphoma, 1 ovarian mass.				
**ILD**	1 patient DRESS-ILD.				
**Drug reaction**	1 patient DRESS-ILD (0.001/100PD), 15 injection site reactions (0.007/100PD).				

**SAE**	55 total events. 6 systemic reaction (0.003/100PD), 4 MAS (0.002/100PD), 21 flares of disease (0.01/100PD), 11 pneumonia (0.005/100PD), 2 pyelonephritis (0.001/100PD), 3 malignancy (0.0015/100PD), 2 epilepsy (0.001/100PD), 2 autoimmune disease (0.001/100PD), 3 inflammatory bowel disease (0.0015/100PD), 1 DRESS-ILD (0.001/100PD), 12 cessation events (0.025/100PD).
**Discontinuation**	12 patients (6.3%). Most common reasons were MAS and systemic exacerbations.
**Death**	Nil.
**Additional notes**	Nil.

**Risk of bias (<6 high, 6-11 moderate, >11 low risk of bias)**					
**Aim**	2	**Consecutive**	1	**Prospective**	0
**Endpoints**	2	**Assessment**	0	**Follow-up**	1
**Loss to follow-up**	0	**Calculation**	0	**Total**	6 (moderate)

CAN canakinumab, CAPS cryopyrin-associated periodic syndromes, DRESS drug reaction with eosinophilia and systemic symptoms, FMF familial mediterranean fever, ILD interstitial lung disease, MKD mevalonate kinase deficiency, PD patient-day, SAE serious adverse event, SJIA systemic onset juvenile idiopathic arthritis, TRAPS TNF receptor-associated periodic fever syndrome

**Study**	Kip 2023				
**Primary reference**	Kip MMA, de Roock S, Currie G, Marshall DA, Grazziotin LR, Twilt M, et al. Pharmacological treatment patterns in patients with juvenile idiopathic arthritis in the Netherlands: a real-world data analysis. Rheumatology (Oxford). 2023;62(SI2):SI170-SI80.				
**Associated references**	Nil		**Study identifier**	Nil	
**Study characteristics**					
**Date of study**	Apr 2011 to Mar 2019	**Design**	Retrospective cohort study	**Condition**	SJIA
**Medication**	ANA (22)<br>CAN (3)<br>(3 had both)	**Number of patients (IL-1 treated)**	236 (25)	**Age**	Not reported
**Follow-up**	Not reported	**Country**	Netherlands	**Sex**	Not reported
**Author's conclusions**	This paper reveals the complexity of pharmacological treatment in JIA, as indicated by: the variety of mono- and combination therapies prescribed, substantial variation in medication prescriptions between subtypes, most patients receiving two or more treatment lines, and the large number of unique treatment sequences.				
**Outcomes**					
**Infection**	Not reported.				

**Malignancy**	Not reported.				
**ILD**	Not reported.				
**Drug reaction**	Not reported.				
**SAE**	Not reported.				
**Discontinuation**	3/33 (9.1%) prescriptions discontinued due to adverse event.				
**Death**	Not reported.				
**Additional notes**	Not reported.				
**Risk of bias (<6 high, 6-11 moderate, >11 low risk of bias)**					
**Aim**	2	**Consecutive**	1	**Prospective**	0
**Endpoints**	1	**Assessment**	0	**Follow-up**	0
**Loss to follow-up**	0	**Calculation**	0	**Total**	4 (high)
ANA anakinra, CAN canakinumab, ILD interstitial lung disease, SAE serious adverse event, SJIA systemic onset juvenile idiopathic arthritis					

**Study**	Kisla Ekinci 2019				
**Primary reference**	Kisla Ekinci RM, Balci S, Dogruel D, Altintas DU, Yilmaz M. Canakinumab in Children with Familial Mediterranean Fever: A Single-Center, Retrospective Analysis. Paediatr Drugs. 2019;21(5):389-95.				
**Associated references**	Nil		**Study identifier**	Nil	
**Study characteristics**					
**Date of study**	Apr 2016 to Apr 2019	**Design**	Retrospective cohort study	**Condition**	FMF
**Medication**	CAN (14)	**Number of patients (IL-1 treated)**	14 (14)	**Age**	Mean 11.2 years (range 4-19)
**Follow-up**	Mean 15.6 months (range 2-60)	**Country**	Türkiye	**Sex**	5 M / 9 F
**Author’s conclusions**	Canakinumab may be an effective treatment option for pediatric FMF patients with colchicine resistance, renal amyloidosis, and chronic oligoarthritis. Further studies are needed to clarify the efficacy of canakinumab in patients with a second disease, RF-positive polyarticular juvenile idiopathic arthritis.				

**Outcomes**					
**Infection**	Nil.				
**Malignancy**	Not reported.				
**ILD**	Not reported.				
**Drug reaction**	Nil.				
**SAE**	Nil.				
**Discontinuation**	Not reported.				
**Death**	Not reported.				
**Additional notes**	Throughout the follow-up, we did not observe any adverse events, including infections, injection-site reactions, cytopenia, or anaphylaxis in any of the 14 patients.				
**Risk of bias (<6 high, 6-11 moderate, >11 low risk of bias)**					
**Aim**	1	**Consecutive**	1	**Prospective**	0
**Endpoints**	2	**Assessment**	0	**Follow-up**	0
**Loss to follow-up**	0	**Calculation**	0	**Total**	4 (high)
CAN canakinumab, FMF familial Mediterranean fever, ILD interstitial lung disease, SAE serious adverse event					

**Study**	Klein 2020				
**Primary reference**	Klein A, Klotsche J, Hugle B, Minden K, Hospach A, Weller-Heinemann F, et al. Long-term surveillance of biologic therapies in systemic-onset juvenile idiopathic arthritis: data from the German BIKER registry. Rheumatology (Oxford). 2020;59(9):2287-98.				
**Associated references**	Atemnkeng Ntam 2021<br>Horneff 2017<br>Thiele 2021		**Study identifier**	Nil	
**Study characteristics**					
**Date of study**	From 2001	**Design**	Registry	**Condition**	SJIA
**Medication**	ANA (71)<br>CAN (51)	**Number of patients (IL-1 treated)**	293 (122)	**Age**	Mean ANA 8.4 years (SD 5.0)<br>Mean CAN 8.8 years (SD 4.8)

**Follow-up**	ANA (121 EY, mean 1.7)<br>CAN (94 EY, mean 1.8)	**Country**	Multiple European countries	**Sex**	ANA 45 M / 23 F<br>CAN 32 M / 19 F
**Author's conclusions**	Surveillance of pharmacotherapy as provided by BIKER is an import approach especially for patients on long-term treatment. Overall, tolerance was acceptable. Differences between several biologics were noted and should be considered in daily patient care.				
**Outcomes**					
**Infection**	Any infectious AE: ANA 8 (11.3%, 19/100EY), CAN 21 (41.2%, 42/100EY).				
**Malignancy**	ANA 1 acute myeloid leukaemia after discontinuing ANA. CAN 0.				
**ILD**	Not reported.				
**Drug reaction**	Anaphylaxis: ANA 0. CAN 1 (2.0%, 1/100EY).				
**SAE**	ANA 5 (7.0%, 8/100EY). CAN 13 (25.5%, 19/100EY).				
**Discontinuation**	Not reported.				
**Death**	ANA 0. CAN 0.				
**Additional notes**	Nil.				
**Risk of bias (<6 high, 6-11 moderate, >11 low risk of bias)**					
**Aim**	2	**Consecutive**	2	**Prospective**	1
**Endpoints**	2	**Assessment**	0	**Follow-up**	0
**Loss to follow-up**	0	**Calculation**	0	**Total**	7 (moderate)
ANA anakinra, CAN canakinumab, EY exposure years, ILD interstitial lung disease, SAE serious adverse event, SJIA systemic onset juvenile idiopathic arthritis					

**Study**	Kone-Paut 2024		
**Primary reference**	Kone-Paut I, Georgin-Lavialle S, Belot A, Jover M, Pouriel M, Lacoin L, et al. Canakinumab treatment real world evidence in 3 monogenic periodic fever syndromes in 2009-2022: an interim analysis using the French JIR cohort database. Arthritis Res Ther. 2024;26(1):80.		
**Associated references**	Cabrera 2019<br>Dumaine 2020	**Study identifier**	NTC02377245
**Study characteristics**			

**Date of study**	Jan 2009 to Jun 2022	**Design**	Registry	**Condition**	FMF (31)<br>MKD (26)<br>TRAPS (7)
**Medication**	CAN (64)	**Number of patients (IL-1 treated)**	64 (64)	**Age**	Mean FMF 14.4 years, MKD 9.7 years, TRAPS 18.9 years
**Follow-up**	Median 3.1 years (range 0-12.0)	**Country**	France	**Sex**	FMF 6 M / 25 F<br>MKD 9 M / 17 F<br>TRAPS 4 M / 3 F
**Author's conclusions**	This interim analysis showed a good maintenance of canakinumab treatment 2 years after initiation and confirmed its safety profile in real-life practice in France in patients diagnosed with FMF, MKD and TRAPS. The high variety of dose and interval combinations observed in canakinumab treated patients let suppose that physicians adapt the posology to individual situations rather than a fixed treatment plan.				
**Outcomes**					
**Infection**	FMF 5, MKD 3, TRAPS 1.				
**Malignancy**	Not reported.				
**ILD**	Not reported.				
**Drug reaction**	Not reported.				
**SAE**	At least one SAE (life threatening, generated permanent disability, required an hospitalisation or a prolongation of the length of stay or could necessitate a medical or surgical intervention) during canakinumab exposure: FMF 4 (12.9%), MKD 1 (3.8%), TRAPS 1 (14.3%)<br>At least one SAE suspected to be related to canakinumab exposure: FMF 2 (6.4%%), MKD 0, TRAPS 1 (14.3%)				
**Discontinuation**	At least one AE leading to canakinumab discontinuation 4/64 (6.2%). FMF 2 (6.4%), MKD 1 (3.8%), TRAPS 1 (14.3%).				
**Death**	None of the patients died during the study period.				
**Additional notes**	Nil.				
**Risk of bias (<6 high, 6-11 moderate, >11 low risk of bias)**					
**Aim**	2	**Consecutive**	2	**Prospective**	1
**Endpoints**	1	**Assessment**	0	**Follow-up**	1
**Loss to follow-up**	0	**Calculation**	0	**Total**	7 (moderate)
CAN canakinumab, FMF familial Mediterranean fever, ILD interstitial lung disease, MKD mevalonate kinase deficiency, SAE serious adverse event, TRAPS TNF receptor-associated periodic fever syndrome					

**Study**	Kullenberg 2016

**Primary reference**	Kullenberg T, Lofqvist M, Leinonen M, Goldbach-Mansky R, Olivecrona H. Long-term safety profile of anakinra in patients with severe cryopyrin-associated periodic syndromes. Rheumatology (Oxford). 2016;55(8):1499-506.				
**Associated references**	Sibley 2012		**Study identifier**	NCT00069329	
**Study characteristics**					
**Date of study**	2003 to 2010	**Design**	Prospective open label study	**Condition**	CAPS
**Medication**	ANA (43)	**Number of patients (IL-1 treated)**	43 (43)	**Age**	36 <18 years, 31 <12 years (range 8-46)
**Follow-up**	Median 4.9 years (159 PYs)	**Country**	United States of America	**Sex**	M 18 / F 25
**Author's conclusions**	In this study anakinra treatment of patients with severe CAPS for up to 5 years was safe and well tolerated both in paediatric and adult patients, with most AEs emerging during the first months after treatment initiation.				
**Outcomes**					
**Infection**	Total infectious events 37 (86.0%) patients and 273 events. Upper respiratory tract infection 17 (39.5%) patients, 48 events. Nasopharyngitis 15 (34.9%) patients, 40 events. Sinusitis 12 (27.9%) patients, 28 events. Ear infection 11 (25.6%) patients, 23 events. Otitis media 11 (25.6%) patients, 20 events. Gastroenteritis 7 (16.3%) patients, 8 events. Gastrointestinal viral infection 6 (14.0%) patients, 8 events. Urinary tract infection 6 (14.0) patients, 10 events. Viral infection 6 (14.0%) patients, 8 events. Pneumonia 5 (11.6%) patients, 6 events. Bronchitis 4 (9.3%), 6 events. Gastrointestinal infection 3 (7.0%) patients, 3 events. Hordeolum 3 (7.0%) patients, 3 events. Otitis externa 3 (7.0%) patients, 3 events. Pharyngitis 3 (7.0%) patients, 4 events. Cellulitis 2 (4.7%), 2 events. Cystitis 2 (4.7%), 6 events. Device related infection 2 (4.7%), 3 events. Otitis media acute 2 (4.7%) patients, 2 events. Pharyngitis streptococcal 2 (4.7%) patients, 6 events.				
**Malignancy**	Not reported.				
**ILD**	Not reported.				
**Drug reaction**	10 patients experienced 17 events, including one vaccination site reaction.				
**SAE**	14 (1.1%) patients experienced 24 SAEs. 4 post-lumbar puncture syndrome, 2 gastroenteritis, 3 pneumonia, 1 cardiac catheterisation, 1 cellulitis, 1 uveitis, 1 wound infection, 1 chest pain, 1 MAS, 1 postoperative wound infection, condition aggravated, 1 meningitis enteroviral, 1 arthritis bacterial, 1 lymphadenitis bacterial, 1 otitis media, 1 traumatic lumbar puncture, 1 convulsion, 1 sinusitis.				
**Discontinuation**	None of the AEs led to discontinuation of the study drug; however, in one patient who developed a wound infection, cellulitis and chest pain, the drug was temporarily stopped. All other patients continued on anakinra,also during infections.				
**Death**	No deaths.				
**Additional notes**	Nil				
**Risk of bias (<6 high, 6-11 moderate, >11 low risk of bias)**					
**Aim**	2	**Consecutive**	2	**Prospective**	2

**Endpoints**	2	**Assessment**	0	**Follow-up**	2
**Loss to follow-up**	1	**Calculation**	0	**Total**	11 (moderate)
ANA anakinra, CAPS cryopyrin-associated periodic syndromes, ILD interstitial lung disease, MAS macrophage activation syndrome, PY patient-year, SAE serious adverse event, USA United States of America					

**Study**	Kurt 2020				
**Primary reference**	Kurt T, Aydin F, Nilufer Tekgoz P, Sezer M, Uncu N, Celikel Acar B. Effect of anti-interleukin-1 treatment on quality of life in children with colchicine-resistant familial Mediterranean fever: A single-center experience. Int J Rheum Dis. 2020;23(7):977-81.				
**Associated references**	Nil		**Study identifier**	Nil	
**Study characteristics**					
**Date of study**	2013 to 2019	**Design**	Retrospective cohort study	**Condition**	FMF (25)
**Medication**	ANA (11)<br>CAN (22)	**Number of patients (IL-1 treated)**	25 (25)	**Age**	14 y (range 8.5-16 y)
**Follow-up**	ANA median 29.7 months (range 8-60)<br>CAN not reported	**Country**	Türkiye	**Sex**	11 M / 14 F
**Author's conclusions**	Anti-IL-1 treatment is quite effective in children with colchicine-resistant FMF patients, proven with improved AIDAI scores and school attendance rates. In the long term by lowering disease activation even development of amyloidosis may be prevented.				
**Outcomes**					
**Infection**	Not reported.				
**Malignancy**	Not reported.				
**ILD**	Not reported.				
**Drug reaction**	1 patient (3.7%) had allergic reactions (severe disseminated rash) with anakinra treatment.				
**SAE**	Not reported.				
**Discontinuation**	2 (7.4%) patients had headache which necessitated termination of treatment with anakinra.				
**Death**	Not reported.				
**Additional notes**	Nil.				

Risk of bias (<6 high, 6-11 moderate, >11 low risk of bias)					
**Aim**	2	**Consecutive**	2	**Prospective**	0
**Endpoints**	1	**Assessment**	0	**Follow-up**	1
**Loss to follow-up**	0	**Calculation**	0	**Total**	6 (moderate)
AIDAI Autoinflammatory Disease Activity Index, ANA anakinra, FMF familial Mediterranean fever, ILD interstitial lung disease, SAE serious adverse event					

**Study**	Lainka 2021				
**Primary reference**	Lainka E, Baehr M, Raszka B, Haas JP, Hugle B, Fischer N, et al. Experiences with IL-1 blockade in systemic juvenile idiopathic arthritis - data from the German AID-registry. Pediatr Rheumatol Online J. 2021;19(1):38.				
**Associated references**	Nil		**Study identifier**	Nil	
**Study characteristics**					
**Date of study**	2009 to 2017	**Design**	Registry	**Condition**	SJIA
**Medication**	ANA (84)<br>CAN (27)<br>(18 had both)	**Number of patients (IL-1 treated)**	111 (111)	**Age**	ANA median 6.8 years (range 0.6-19.1)<br>CAN median 8.7 years (range 2.2-19.1)
**Follow-up**	ANA median 34 m (range 6-116) (228 EY)<br>CAN median 16 m (range 4-58) (56.4 EY)	**Country**	Germany	**Sex**	57 M / 54 F
**Author's conclusions**					
**Outcomes**					
**Infection**	ANA 15 events (19.7/100PY). CAN 6 events (10.6/100PY).				
**Malignancy**	Not reported.				
**ILD**	Not reported.				
**Drug reaction**	ANA 5 local site reaction / aversion (2.2/100PY). CAN 1 local site reaction / aversion (1.8/100PY).				

**SAE**	Not reported.				
**Discontinuation**	ANA 4/84 discontinued due to AE. 2 WHO toxicity II or III, 2 trypanophobia. CAN no discontinuation due to AE.				
**Death**	No deaths.				
**Additional notes**	Nil.				
**Risk of bias (<6 high, 6-11 moderate, >11 low risk of bias)**					
**Aim**	1	**Consecutive**	1	**Prospective**	1
**Endpoints**	1	**Assessment**	0	**Follow-up**	1
**Loss to follow-up**	0	**Calculation**	0	**Total**	5 (high)
EY exposure years, ILD interstitial lung disease, PY patient year, SAE serious adverse event, SJIA systemic onset juvenile idiopathic arthritis, WHO world health organisation					

**Study**	Lequerre 2008				
**Primary reference**	Lequerre T, Quartier P, Rosellini D, Alaoui F, De Bandt M, Mejjad O, et al. Interleukin-1 receptor antagonist (anakinra) treatment in patients with systemic-onset juvenile idiopathic arthritis or adult onset Still disease: preliminary experience in France. Ann Rheum Dis. 2008;67(3):302-8.				
**Associated references**	Nil		**Study identifier**	Nil	
**Study characteristics**					
**Date of study**	Before Dec 2005	**Design**	Prospective cohort study	**Condition**	SJIA (20), AOSD (15)
**Medication**	ANA (35)	**Number of patients (IL-1 treated)**	35 (35)	**Age**	SJIA mean 12.4 years (SD 5.2) AOSD mean 38.1 years (SD 12.8)
**Follow-up**	Mean 14.4 months (range 1 to 27)	**Country**	France	**Sex**	12 M / 23 F
**Author's conclusions**	Anakinra was effective in most AoSD patients, but less than half SoJIA patients achieved a marked and sustained improvement.				
**Outcomes**					
**Infection**	2/35 (5.7%) varicella, 2/35 (5.7%) rhinopharyngitis, 1/35 (2.9%) non-extensive labial herpes, 1/35 (2.9%) bronchitis, 1/35 (2.9%) uncomplicated hepatitis A infection, 1/35 (2.9%) cutaneous infection after a piercing.				

**Malignancy**	Not reported.
**ILD**	Not reported.
**Drug reaction**	18/20 (90%) SJIA patients and 1/15 (6.7%) AOSD patient reported pain during injections. SoJIA patients also experienced local inflammation at the injection site or pruritus during the first few weeks,with a favourable outcome afterwards.Two AOSD patients developed a skin rash after 1 month and 3 months, respectively, leading to the withdrawal of anakinra.
**SAE**	1/35 (2.9%) developed visceral leishmania infection during month 6 of anakinra treatment. The child was living in an endemic zone in the south of France, but her pre-therapeutic blood sample was negative. Anakinra treatment was stopped, and treatment specific for Leishmania was started and resulted in a favourable outcome. SoJIA became active again following anakinra treatment withdrawal; therefore, anakinra was recently restarted in this patient.
**Discontinuation**	3/35 (8.6%) discontinued due to intolerance or side-effects.
**Death**	Not reported.
**Additional notes**	Nil.

**Risk of bias (<6 high, 6-11 moderate, >11 low risk of bias)**					
**Aim**	2	**Consecutive**	0	**Prospective**	1
**Endpoints**	2	**Assessment**	0	**Follow-up**	0
**Loss to follow-up**	1	**Calculation**	0	**Total**	6 (moderate)

ANA anakinra, AOSD adult onset Still's disease, ILD interstitial lung disease, SAE serious adverse event, SJIA systemic onset juvenile idiopathic arthritis

**Study**	Lovell 2013				
**Primary reference**	Lovell DJ, Giannini EH, Reiff AO, Kimura Y, Li S, Hashkes PJ, et al. Long-term safety and efficacy of rilonacept in patients with systemic juvenile idiopathic arthritis. Arthritis Rheum. 2013;65(9):2486-96.				
**Associated references**	Nil		**Study identifier**	NCT01803321	
**Study characteristics**					
**Date of study**	Dec 2005 to Jun 2008	**Design**	RCT LTE	**Condition**	SJIA
**Medication**	RIL (23)	**Number of patients (IL-1 treated)**	23 (23)	**Age**	Mean 12.6 years (SD 4.3)<br>Median 14.0 years (range 5-20)
**Follow-up**	30.27 PYs (mean 1.3)	**Country**	United States of America	**Sex**	8 M / 16 F

**Author's conclusions**	Sustained improvements in clinical and laboratory measures of the articular and systemic manifestations of systemic JIA were achieved in >50% of rilonacept-treated patients over 2 years. Treatment with rilonacept had a substantial steroid-sparing effect and was generally well-tolerated.				
**Outcomes**					
**Infection**	Infections in 16/23 (69.5%). Upper respiratory tract infection 5/23 (21/7%), viral gastroenteritis 4/23 (17.3%), nasopharyngitis 3/23 (13.0%), skin infection 3/23 (13%), ear infection 2/23 (8.6%), hordeolum 2/23 (8.6%), influenza 2/23 (8.6%). None of the infections were considered serious, and no patients withdrew due to infection.				
**Malignancy**	0.				
**ILD**	1/23 (4.3%) developed pulmonary fibrosis and MAS.				
**Drug reaction**	Injection site reactions in one third of patients in the double blind phase. Injection-site erythema was noted in rilonacept-treated patients only, and injection-site bruising was reported both in patients receiving placebo and in patients receiving rilonacept.				
**SAE**	1/24 (4.2%, 2 events) in the double-blind phase and 3/23 (13.0%, 6 events) in the LTE had at least 1 SAE. 2 (8.6%) arthritis flare, 2 (8.6%) MAS, 1 pancytopaenia (4.3%), 1 pyrexia (4.3%), 1 (4.3%) anaemia, 1 (4.3%) pulmonary fibrosis. None of the SAEs were deemed to be directly related to rilonacept by the treating physician. Injection-site reactions were the most common treatment-related AE during the open-label period (Table 2), occurring in 60.9% of patients. Nearly all injection-site reactions were considered mild; none were severe. Of the patients who experienced injection-site reactions, 35.7% had 2 events, 42.9% had 3–8 events, and 21.4% had 8 events. All patients who experienced 3 injection-site reactions were positive for antirilonacept antibodies.				
**Discontinuation**	3/23 (13.0%). 1 (4.3%) depression, 1 (4.3%) injection site reactions, 1 (4.3%) pulmonary fibrosis / MAS.				
**Death**	0.				
**Additional notes**	Thirteen of 24 patients in the long-term open-label extension phase developed antirilonacept antibodies. There appeared to be little or no correlation between responses to the antirilonacept antibody assay and decreases in plasma drug levels or clinical responses in these patients.				
**Risk of bias (<6 high, 6-11 moderate, >11 low risk of bias)**					
**Aim**	2	**Consecutive**	2	**Prospective**	2
**Endpoints**	2	**Assessment**	1	**Follow-up**	0
**Loss to follow-up**	2	**Calculation**	0	**Total**	11 (moderate)
ILD interstitial lung disease, LTE long term extension, MAS macrophage activation syndrome, PT patient-year, RCT randomised controlled trial, RIL rilonacept, SAE serious adverse event, SD standard deviation, SJIA systemic onset juvenile idiopathic arthritis, USA united states of America					

**Study**	Neven 2010
**Primary reference**	Neven B, Marvillet I, Terrada C, Ferster A, Boddaert N, Couloignier V, et al. Long-term efficacy of the interleukin-1 receptor antagonist anakinra in ten patients with neonatal-onset multisystem inflammatory disease/chronic infantile neurologic, cutaneous, articular syndrome. Arthritis Rheum. 2010;62(1):258-67.

**Associated references**	NIl		**Study identifier**	Nil	
**Study characteristics**					
**Date of study**	Before June 2007	**Design**	Retrospective cohort study	**Condition**	CAPS
**Medication**	ANA (10)	**Number of patients (IL-1 treated)**	10 (10)	**Age**	14.75 years (6-19.8years) for 8 pts, others were 3mo and 4mo
**Follow-up**	26-42 months	**Country**	France	**Sex**	3 M / 7F
**Author's conclusions**	Anakinra was effective in the long term for CINCA/NOMID. However, it needs to be started before irreversible lesions develop.				
**Outcomes**					
**Infection**	Nil.				
**Malignancy**	Not reported.				
**ILD**	Not reported.				
**Drug reaction**	Mild local stinging and erythema (number not reported).				
**SAE**	Not reported.				
**Discontinuation**	Not reported.				
**Death**	Not reported.				
**Additional notes**	Nil.				
**Risk of bias (<6 high, 6-11 moderate, >11 low risk of bias)**					
**Aim**	1	**Consecutive**	1	**Prospective**	0
**Endpoints**	1	**Assessment**	0	**Follow-up**	1
**Loss to follow-up**	0	**Calculation**	0	**Total**	4 (high)
ANA anakinra, CAPS cryopyrin-associated periodic syndromes, ILD interstitial lung disease, SAE serious adverse event					

**Study**	Nigrovic 2011

**Primary reference**	Nigrovic PA, Mannion M, Prince FH, Zeft A, Rabinovich CE, van Rossum MA, et al. Anakinra as first-line disease-modifying therapy in systemic juvenile idiopathic arthritis: report of forty-six patients from an international multicenter series. Arthritis Rheum. 2011;63(2):545-55.				
**Associated references**	Nil		**Study identifier**	Nil	
**Study characteristics**					
**Date of study**	Before June 1 2010	**Design**	Retrospective cohort study	**Condition**	SJIA
**Medication**	ANA (46)	**Number of patients (IL-1 treated)**	46 (46)	**Age**	7.6 years (0.75-15.7)
**Follow-up**	Median 14.5 months	**Country**	International	**Sex**	M 19 / F 27
**Author's conclusions**	Anakinra as a first line for JIA had rapid resolution of systemic symptoms and prevention of refractory arthritis in almost 90% of patients.				
**Outcomes**					
**Infection**	3 cases of serious infection, 2 episodes of bronchitis in 1 pt, recurrent viral esp illness in1 pt				
**Malignancy**	Not reported				
**ILD**	Not reported				
**Drug reaction**	Injection site reaction was seen in 44% of patients				
**SAE**	Not reported				
**Discontinuation**	1 due to injection site reaction, 1 due to eosinophilic hepatitis.				
**Death**	Not reported				
**Additional notes**	11 episodes of macrophage activation syndrome in 9 patients (20%)				
**Risk of bias (<6 high, 6-11 moderate, >11 low risk of bias)**					
**Aim**	2	**Consecutive**	1	**Prospective**	0
**Endpoints**	2	**Assessment**	0	**Follow-up**	0
**Loss to follow-up**	0	**Calculation**	0	**Total**	5 (high)
ANA anakinra, ILD interstitial lung disease, SAE serious adverse event, SJIA systemic onset juvenile idiopathic arthritis					

**Study**	Nur Sunar Yayla 2024				
**Primary reference**	Nur Sunar Yayla E, Yildiz C, Esmeray Senol P, Karacayir N, Gezgin Yildirim D, Bakkaloglu SA. How Safe Are Biological Agents in Pediatric Rheumatology? Turk Arch Pediatr. 2024;59(2):185-92.				
**Associated references**	Nil		**Study identifier**	Nil	
**Study characteristics**					
**Date of study**	Jan 2010- Jan 2022	**Design**	Retrospective cohort study	**Condition**	JIA (65), FMF (41), uveitis (10), Behçet's (7), HIDS (2), JDM (2), other
**Medication**	ANA (43)<br>CAN (33)	**Number of patients (IL-1 treated)**	139 (76)	**Age**	Median 11 years (0.7-20.3)
**Follow-up**	Median treatment duration 45 months	**Country**	Türkiye	**Sex**	M 106 / F 73
**Author's conclusions**	The most commonly used biological treatments were TNFi and IL-antagonists, and the majority of side effects were infections and laboratory abnormalities.				
**Outcomes**					
**Infection**	ANA 26 events (60.5/100PY). CAN 89 events (77.4/100PY).				
**Malignancy**	0.				
**ILD**	Not reported.				
**Drug reaction**	5 injection site reactions. No anaphylaxis.				
**SAE**	Not reported.				
**Discontinuation**	11 discontinued due to side-effects across the whole cohort but not reported for anti-IL-1 treatments specifically..				
**Death**	Not reported.				
**Additional notes**	1 MAS.				
**Risk of bias (<6 high, 6-11 moderate, >11 low risk of bias)**					
**Aim**	1	**Consecutive**	1	**Prospective**	0
**Endpoints**	1	**Assessment**	0	**Follow-up**	1

Loss to follow-up	0	Calculation	0	Total	4 (high)
ANA anakinra, CAN canakinumab, FMF familial mediterranean fever, HIDS hyper-IgD syndrome, ILD interstitial lung disease, JDM juvenile dermatomyositis, JIA juvenile idiopathic arthritis, MAS macrophage activation syndrome, PY patient-year SAE serious adverse event					

Study	Özçakar 2016				
Primary reference	Ozcakar ZB, Ozdel S, Yilmaz S, Kurt-Sukur ED, Ekim M, Yalcinkaya F. Anti-IL-1 treatment in familial Mediterranean fever and related amyloidosis. Clin Rheumatol. 2016;35(2):441-6.				
Associated references	Nil.		Study identifier	Nil.	
Study characteristics					
Date of study	Not reported	Design	Retrospective cohort study	Condition	FMF
Medication	ANA (10)<br>CAN (3)	Number of patients (IL-1 treated)	330 (13)	Age	Mean 14.5 years (range 6 to 22)
Follow-up	Mean 18.1 months (range 9 to 40)	Country	Türkiye	Sex	Not reported.
Author's conclusions	Anti-IL-1 therapies can be successfully used in colchicine resistant FMF patients and patients with amyloidosis during childhood and adolescent period without major side effects.				
Outcomes					
Infection	Not reported.				
Malignancy	Not reported.				
ILD	Not reported.				
Drug reaction	Not reported.				
SAE	Not reported.				
Discontinuation	Not reported.				
Death	Not reported.				
Additional notes	None of the patients had adverse events and/or drug related side effects during treatment.				
Risk of bias (<6 high, 6-11 moderate, >11 low risk of bias)					

Aim	1	Consecutive	1	Prospective	0
**Endpoints**	0	**Assessment**	0	**Follow-up**	0
**Loss to follow-up**	0	**Calculation**	0	**Total**	2 (high)
ANA anakinra, CAN canakinumab, FMF familial mediterranean fever, ILD interstitial lung disease, SAE serious adverse event					

**Study**	Ozen 2020				
**Primary reference**	Ozen S, Ben-Cherit E, Foeldvari I, Amarilyo G, Ozdogan H, Vanderschueren S, et al. Long-term efficacy and safety of canakinumab in patients with colchicine-resistant familial Mediterranean fever: results from the randomised phase III CLUSTER trial. Ann Rheum Dis. 2020;79(10):1362-9.				
**Associated references**	Jeyaratnam 2022		**Study identifier**	Nil	
**Study characteristics**					
**Date of study**	Unknown	**Design**	RCT	**Condition**	Colchicine resistant FMF
**Medication**	CAN (60)	**Number of patients (IL-1 treated)**	60 (60)	**Age**	Median 18 years (14.0 to 29.5)
**Follow-up**	113 weeks	**Country**	Multiple	**Sex**	M 32 / F 28
**Author's conclusions**	crFMF patients treated with canakinumab during 72 weeks experienced a minimal incidence of flares and good control of clinical disease activity, with no new safety concerns reported.				
**Outcomes**					
**Infection**	0.36 in patient days, 107 in event rate.				
**Malignancy**	Not reported.				
**ILD**	Not reported.				
**Drug reaction**	Not reported.				
**SAE**	23 SAEs. 0.08 in patient days, 23 in event rate.				
**Discontinuation**	1 due to pyoderma gangrenosum, 1 due to patient preference, 1 due to pregnancy.				
**Death**	0.				
**Additional notes**	Nil.				

Risk of bias (<6 high, 6-11 moderate, >11 low risk of bias)					
**Aim**	2	**Consecutive**	2	**Prospective**	2
**Endpoints**	2	**Assessment**	1	**Follow-up**	1
**Loss to follow-up**	2	**Calculation**	0	**Total**	12 (low)
CAN canakinumab, FMF familial mediterranean fever, ILD interstitial lung disease, SAE serious adverse event					

**Study**	Pinchevski-Kadir 2023				
**Primary reference**	Pinchevski-Kadir S, Gerstein M, Pleniceanu O, Yacobi Y, Vivante A, Granat OE, et al. Effect of interleukin-1 antagonist on growth of children with colchicine resistant or intolerant FMF. Pediatr Rheumatol Online J. 2023;21(1):4.				
**Associated references**	Nil		**Study identifier**	Nil	
**Study characteristics**					
**Date of study**	2006 to 2022	**Design**	Retrospective cohort study	**Condition**	FMF
**Medication**	ANA (8)<br>CAN (20)<br>(6 had both)	**Number of patients (IL-1 treated)**	22 (22)	**Age**	Mean 12.85 years (SD 4.2)
**Follow-up**	Mean 3.05 years (SD 1.75)	**Country**	Israel	**Sex**	M 8 / F 14
**Author's conclusions**	Treatment with anti-IL-1 agents in children with FMF is effective and safe and may potentiate long-term growth.				
**Outcomes**					
**Infection**	Not reported.				
**Malignancy**	Not reported.				
**ILD**	Not reported.				
**Drug reaction**	1 (4.5%) local skin reaction.				
**SAE**	Not reported.				
**Discontinuation**	Not reported.				

**Death**	Not reported.				
**Additional notes**	Overall, during 771 patient months of followup, one patient reported a local skin reaction at the injection site, and two patients complained of abdominal pain. Notably, no additional side effects were noted. We found a significant increase in height and body weight percentiles following treatment.				
**Risk of bias (<6 high, 6-11 moderate, >11 low risk of bias)**					
**Aim**	1	**Consecutive**	1	**Prospective**	0
**Endpoints**	1	**Assessment**	0	**Follow-up**	1
**Loss to follow-up**	1	**Calculation**	0	**Total**	5 (high)
ANA anakinra, CAN canakinumab, FMF familial Mediterranean fever, ILD interstitial lung disease, SAE serious adverse event, SD standard deviation					

**Study**	Quartier 2021				
**Primary reference**	Quartier P, Alexeeva E, Constantin T, Chasnyk V, Wulffraat N, Palmblad K, et al. Tapering Canakinumab Monotherapy in Patients With Systemic Juvenile Idiopathic Arthritis in Clinical Remission: Results From a Phase IIIb/IV Open-Label, Randomized Study. Arthritis rheumatol. 2021;73(2):336-46.				
**Associated references**	Nil		**Study identifier**	NCT00891046<br>NCT02296424	
**Study characteristics**					
**Date of study**	Nov 2014 to Sep 2017	**Design**	RCT	**Condition**	SJIA
**Medication**	CAN (182)	**Number of patients (IL-1 treated)**	182 (182)<br><br>(75 included in part 2)	**Age**	C1 median 12 years (IQR 8-15)<br>C2 8 years (5-12)<br>DR 10.5 years (7-15)<br>DP 11 years (9-14)
**Follow-up**	C1 mean 466 days<br>C2 mean 434 days<br>DR mean 489 days<br>DP mean 495 days	**Country**	16 countries	**Sex**	112 M / 129 F
**Author's conclusions**	Reduction of canakinumab exposure may be feasible in patients who have achieved clinical remission of systemic JIA, but consistent interleukin-1 inhibition appears necessary to maintain this response.				
**Outcomes**					
**Infection**	Part 1 (C1+C2) pyrexia 76 events (0.1/100PYs), nasopharyngitis 70 events (0.09/100PYs), diarrhoea 42 (0.06/100PYs), cough 39 events (0.05/100PYs), upper respiratory tract infection 40 events (0.05/100PYs), viral infection 12 events (0.02/100PYs)<br>Part 2 (DR) pyrexia 17 events (0.07/100PYs), nasopharyngitis 16 events (0.07/100PYs), diarrhoea 7 (0.03/100PYs), cough 11 events (0.05/100PYs), upper respiratory tract infection 16 events (0.07/100PYs), viral infection 12 events (0.05/100PYs)				

	Part 2 (DP) pyrexia 13 events (0.06/100PYs), nasopharyngitis 18 events (0.08/100PYs), diarrhoea 2 (0.01/100PYs), cough 3 events (0.01/100PYs), upper respiratory tract infection 6 events (0.03/100PYs), viral infection 3 events (0.01/100PYs).
**Malignancy**	Not reported.
**ILD**	Two SAEs related to lung disease were reported, including a mild case of interstitial lung disease and a severe case of alveolar proteinosis that led to study discontinuation for that individual.
**Drug reaction**	Not reported.
**SAE**	Part 1 (C1+C2) 49 (0.07/100PYs). 13 infections/infestations, 14 musculoskeletal.connective tissue disorder, 5 blood/lymphatic disorder, 4 gastrointestinal disorder.<br>Part 2 (DR) 4 (0.02/100PYs). 2 infections/infestations, 1 blood/lymphatic disorder.<br>Part 2 (DP) 2 (0.01/100PYs). 2 blood/lymphatic disorder.
**Discontinuation**	Part 1 (C1+C2) 15 (0.02/100PYs). Study treatment discontinuation due to AEs occurred in a higher proportion of patients in cohort 2 (12.2%) compared to cohort 1 (4.4%). The most common reasons for discontinuation were disease related, namely MAS and AEs related to systemic JIA worsening or disease flares.<br>Part 2 (DR) 1 (<0.01/100PYs). Only 1 patient in part 1 (in the dose reduction arm) experienced an AE (blepharitis) that led to study discontinuation.<br>Part 2 (DP) 0.
**Death**	No deaths were reported during the study.
**Additional notes**	Nil.

**Risk of bias (<6 high, 6-11 moderate, >11 low risk of bias)**					
**Aim**	2	**Consecutive**	1	**Prospective**	2
**Endpoints**	2	**Assessment**	0	**Follow-up**	0
**Loss to follow-up**	2	**Calculation**	1	**Total**	10 (moderate)

AE adverse event, C1 cohort 1 (part 1 of the study), C2 cohort 2 (part 1 of the study), CAN canakinumab, DR dose reduction arm (part 2 of the study), DP dose prolongation arm (part 2 of the study), ILD interstitial lung disease, IQR interquartile range, LTE long term extension, MAS macrophage activation syndrome, PY patient-year, RCT randomised controlled trial, SAE serious adverse event, SJIA systemic onset juvenile idiopathic arthritis

**Study**	Ruperto 2018				
**Primary reference**	Ruperto N, Brunner HI, Quartier P, Constantin T, Wulffraat NM, Horneff G, et al. Canakinumab in patients with systemic juvenile idiopathic arthritis and active systemic features: results from the 5-year long-term extension of the phase III pivotal trials. Ann Rheum Dis. 2018;77(12):1710-9.				
**Associated references**	Brunner 2020		**Study identifier**	NCT00426218<br>NCT00886769<br>NCT00889863<br>NCT00891046	
**Study characteristics**					
**Date of study**	Jul 2009 to Dec 2014	**Design**	RCT LTE	**Condition**	SJIA

**Medication**	CAN (177)	**Number of patients (IL-1 treated)**	177 (177)	**Age**	9.0 years (IQR 6-13)
**Follow-up**	Median 3.5 years (IQR 0.6 to 4.4) for CAN and placebo	**Country**	21 countries	**Sex**	M 65 / F 79
**Author's conclusions**	Response to canakinumab treatment was sustained and associated with substantial glucocorticoid dose reduction or discontinuation and a relatively low retention-on-treatment rate. no new safety findings were observed on long-term use of canakinumab.				
**Outcomes**					
**Infection**	1036 infection and infestation events (217.3/100PYs). The incidence of serious infections was 10.3/100PYs and all resolved without sequelae. Most common serious infections were gastroenteritis (1.1/100PYs), pneumonia (0.8/100PYs), and varicella, subcutaneous abscess, gastrointestinal viral infection, septic shock and streptococcal tonsillitis (0.4/100PYs each).				
**Malignancy**	No malignancies were reported.				
**ILD**	One of the reported MAS events was complicated by pulmonary hypertension and interstitial pneumonia, resulting in patient death in the pivotal study as previously reported. One event was complicated by transfusion-related acute lung injury; acute interstitial pneumonitis, following blood transfusion products f				
**Drug reaction**	2 drug reaction with eosinophilia and systemic symptoms (0.4/100PYs). No anaphylaxis or anaphylactoid reactions were reported.				
**SAE**	194 events (40.7/100PYs). 25 juvenile idiopathic arthritis (5/2/100PYs), 17 MAS (3.6/100PYs), 8 fever (1.7/100PYs), 5 gastroenteritis (1.1/100PYs), 4abdominal pain (0.8/100PYs), 4 pneumonia (0.8/100PYs), 3 hepatitis (0.6/100PYs), 3 hepatic enzyme increased (0.6/100PYs), 2 septic shock (0.4/100PYs), 2 arthralgia (0.4/100PYs), 2 lymphadenopathy (0.4/100PYs), 2 gastrointestinal viral infecion (0.4/100PYs), 2 subcutaneous abscess (0.4/100PYs), 2 tonsillitis streptococcal (0.4/100PYs), 2 musculoskeletal chest pain (0.4/100PYs), 2 varicella (0.4/100PYs), 2 vomiting (0.4/100PYs), 2 drug reaction with eosinophilia and systemic symptoms (0.4/100PYs), 2 C-reactive protein increased (0.4/100PYs), 2 serum ferritin increased (0.4/100PYs), 2 parasthesia (0.4/100PYs), 2 traumatic fracture (0.4/100PYs), 2 rash (0.4/100PYs).				
**Discontinuation**	19/177 (10.7%) discontinued due to intolerance.				
**Death**	3/177. One death occurred during part I; patient died due to MAS (on canakinumab). A patient in the placebo group died due to MAS 2 days after discontinuing the part II phase due to MAS (not on canakinumab). One patient died from disease progression 3 months after discontinuation from the long-term extension phase due to unsatisfactory therapeutic effect (not on canakinumab).				
**Additional notes**	Nil.				
**Risk of bias (<6 high, 6-11 moderate, >11 low risk of bias)**					
**Aim**	2	**Consecutive**	2	**Prospective**	2
**Endpoints**	2	**Assessment**	0	**Follow-up**	1
**Loss to follow-up**	2	**Calculation**	0	**Total**	11 (moderate)
CAN canakinumab, ILD interstitial lung disease, IQR interquartile range, LTE long term extension, PY patient-year, RCT randomised controlled trial, SAE serious adverse event, SJIA systemic onset juvenile idiopathic arthritis					

Study	Ruperto 2012				
**Primary reference**	Ruperto N, Quartier P, Wulffraat N, Woo P, Ravelli A, Mouy R, et al. A phase II, multicenter, open-label study evaluating dosing and preliminary safety and efficacy of canakinumab in systemic juvenile idiopathic arthritis with active systemic features. Arthritis Rheum. 2012;64(2):557-67.				
**Associated references**	Nil		**Study identifier**	2006-001834-42	
**Study characteristics**					
**Date of study**	Not reported	**Design**	Open label study	**Condition**	SJIA
**Medication**	CAN (23)	**Number of patients (IL-1 treated)**	23 (23)	**Age**	Median 10 years (range 4-19)
**Follow-up**	>1 year for 12/23 (52.2%) >2 years for 7/23 (30.4%)	**Country**	Multiple	**Sex**	M 12 / F 11
**Author's conclusions**	Canakinumab has a promising preliminary safety and efficacy profile in this limited cohort. Based on the findings of this trial, further studies in a larger population of children with systemic JIA are warranted.				
**Outcomes**					
**Infection**	8 (35%) pyrexia, 6 (26%) gastroenteritis, 6 (26%) rhinitis, 4 (17%) pharyngitis, 4 (17%) pharyngeal erythema, 4 (17%) nasopharyngitis, 3 (13%) acute tonsillitis, 3 (13%) upper respiratory tract infection.				
**Malignancy**	Not reported.				
**ILD**	Not reported.				
**Drug reaction**	Canakinumab injections were well tolerated, and there were no reports of severe injection-site reactions.				
**SAE**	11/23 (47.8%). In 2 patients (1 with Epstein-Barr virus infection during and 1 with hematoma, prolonged activated partial thromboplastin time, gastroenteritis, and syncope), these were suspected to be related to the study drug. All AEs resolved spontaneously or with appropriate medication.				
**Discontinuation**	No patients discontinued the study drug because of adverse events.				
**Death**	No deaths were reported during the study. A 22-year-old female patient died of pneumococcal sepsis 2.25 years after the last canakinumab injection; she had received 2 injections of 1.5 mg/kg during the study.				
**Additional notes**	Nil.				
**Risk of bias (<6 high, 6-11 moderate, >11 low risk of bias)**					
**Aim**	2	**Consecutive**	1	**Prospective**	2
**Endpoints**	2	**Assessment**	0	**Follow-up**	0
**Loss to follow-up**	0	**Calculation**	0	**Total**	7 (moderate)

CAN canakinumab, ILD interstitial lung disease, SAE serious adverse event, SJIA systemic onset juvenile idiopathic arthritis

**Study**	Russo 2014				
**Primary reference**	Russo RA, Melo-Gomes S, Lachmann HJ, Wynne K, Rajput K, Eleftheriou D, et al. Efficacy and safety of canakinumab therapy in paediatric patients with cryopyrin-associated periodic syndrome: a single-centre, real-world experience. Rheumatology (Oxford). 2014;53(4):665-70.				
**Associated references**	Nil		**Study identifier**	Nil	
**Study characteristics**					
**Date of study**	May 2010 to Dec 2012	**Design**	Retrospective case review	**Condition**	CAPS
**Medication**	CAN (10)	**Number of patients (IL-1 treated)**	10 (10)	**Age**	6.3 years (range 4.0-13.6)
**Follow-up**	Median 21 months (range 12-31)	**Country**	United Kingdom	**Sex**	6 M/ 4 F
**Author's conclusions**	Canakinumab, although costly, is a safe and effective treatment for CAPS in children, leading to sustained improvement in disease activity, serological markers, functional ability and HRQoL.				
**Outcomes**					
**Infection**	Three children developed four infections (2 chickenpox, 1 pneumonia, 1 presumed infective gastroenteritis).				
**Malignancy**	Not reported.				
**ILD**	Not reported.				
**Drug reaction**	No injection site reactions.				
**SAE**	0.				
**Discontinuation**	Not reported.				
**Death**	Not reported.				
**Additional notes**	Nil.				
**Risk of bias (<6 high, 6-11 moderate, >11 low risk of bias)**					
**Aim**	1	**Consecutive**	1	**Prospective**	0

**Endpoints**	2	**Assessment**	0	**Follow-up**	0
**Loss to follow-up**	0	**Calculation**	0	**Total**	4 (high)
CAN canakinumab, CAPS cryopyrin-associated periodic syndromes, ILD interstitial lung disease, SAE serious adverse event					

**Study**	Sag 2020				
**Primary reference**	Sag E, Akal F, Atalay E, Akca UK, Demir S, Demirel D, et al. Anti-IL1 treatment in colchicine-resistant paediatric FMF patients: real life data from the HELIOS registry. Rheumatology (Oxford). 2020;59(11):3324-9.				
**Associated references**	Nil		**Study identifier**	HELIOS	
**Study characteristics**					
**Date of study**	Not reported	**Design**	Registry	**Condition**	FMF
**Medication**	ANA (40)<br>CAN (28)<br>(28 had both)	**Number of patients (IL-1 treated)**	40 (40)	**Age**	Mean 11.5 years (SD 5.4)
**Follow-up**	Mean 3.87 years (SD 1.96)	**Country**	Türkiye	**Sex**	17 M / 27 F
**Author's conclusions**	Anakinra and canakinumab are efficient and safe alternatives in colchicine-resistant or -intolerant paediatric FMF patients. We also, for the first time, report on-demand use of anti-IL1 in paediatric FMF patients.				
**Outcomes**					
**Infection**	Three episodes of mild infections leading to hospitalization.				
**Malignancy**	Not reported.				
**ILD**	Not reported.				
**Drug reaction**	Eleven patients had local skin reactions secondary to ANA.				
**SAE**	Not reported.				
**Discontinuation**	11/40 patients who had local skin reactions secondary to ANA changed to CAN.				
**Death**	Not reported.				
**Additional notes**	Two patients had transient leukopenia with anakinra and another patient had transient thrombocytopenia with canakinumab.				

Risk of bias (<6 high, 6-11 moderate, >11 low risk of bias)					
**Aim**	1	**Consecutive**	1	**Prospective**	1
**Endpoints**	1	**Assessment**	0	**Follow-up**	1
**Loss to follow-up**	0	**Calculation**	0	**Total**	5 (high)
ANA anakinra, CAN canakinumab, FMF familial mediterranean fever, ILD interstitial lung disease, SAE serious adverse event, SD standard deviation					

**Study**	Shehadeh 2024				
**Primary reference**	Shehadeh K, Levinsky Y, Kagan S, Zuabi T, Tal R, Aviran NH, et al. An "On Demand" canakinumab regimen for treating children with Colchicine-Resistant familial Mediterranean fever - A multicentre study. Int Immunopharmacol. 2024;132:111967.				
**Associated references**	Nil		**Study identifier**	Nil	
**Study characteristics**					
**Date of study**	Dec 2017 and Jun 2022	**Design**	Retrospective cohort study	**Condition**	FMF
**Medication**	CAN (51)	**Number of patients(IL-1 treated)**	51 (51)	**Age**	COD 2.22 years (0.73, 2.99)<br>CFF 2.48 years (1.21, 3.62)
**Follow-up**	Up to 18 months	**Country**	Israel	**Sex**	24 M / 27 F
**Author's conclusions**	COD policy in crFMF can achieve non-inferior efficacy and safety results using less than half the accumulated canakinumab dose. This approach may be less expensive and less immunosuppressive.				
**Outcomes**					
**Infection**	Not reported.				
**Malignancy**	Not reported.				
**ILD**	Not reported.				
**Drug reaction**	Not reported.				
**SAE**	Not reported.				
**Discontinuation**	Not reported.				

**Death**	Not reported.				
**Additional notes**	The only documented adverse event that was deemed as directly related to canakinumab was an event of headaches, graded as 1 by the RCTC score, in each group.				
**Risk of bias (<6 high, 6-11 moderate, >11 low risk of bias)**					
**Aim**	2	**Consecutive**	2	**Prospective**	0
**Endpoints**	2	**Assessment**	0	**Follow-up**	0
**Loss to follow-up**	0	**Calculation**	0	**Total**	6 (moderate)
CAN canakinumab, CFF canakinumab fixed frequency, COD canakinumab on demand, FMF familial mediterranean fever, ILD interstitial lung disease, RCTC rheumatology common toxicity criteria, SAE serious adverse event					

**Study**	Sibley 2012				
**Primary reference**	Sibley CH, Plass N, Snow J, Wiggs EA, Brewer CC, King KA, et al. Sustained response and prevention of damage progression in patients with neonatal-onset multisystem inflammatory disease treated with anakinra: a cohort study to determine three- and five-year outcomes. Arthritis Rheum. 2012;64(7):2375-86.				
**Associated references**	Kullenberg 2016		**Study identifier**	NCT00069329	
**Study characteristics**					
**Date of study**	Sep 2003 to Apr 2010	**Design**	Prospective cohort	**Condition**	CAPS
**Medication**	ANA (26)	**Number of patients(IL-1 treated)**	26 (26)	**Age**	Mean 11.5 years (SD 9.12)
**Follow-up**	Mean 5.7 years (total 148.1 PYs)	**Country**	United States of America	**Sex**	13 M / 13 F
**Author's conclusions**	These findings indicate that anakinra provides sustained efficacy in the treatment of NOMID for up to 5 years, with the requirement of dose escalation. Damage progression in the CNS, ear, and eye, but not bone, is preventable. Anakinra is well tolerated overall.				
**Outcomes**					
**Infection**	Low dose anakinra (≤2.5mg/ kg/day, 69.2 PYs)<br>6 cellulitis, 3 fungal skin infections, 17 ear infections, 12 gastroenteritis, 0 pneumonia, 14 sinusitis, 58 upper respiratory tract infections, 12 urinary tract infections, 3 streptococcal pharyngitis.<br><br>High-dose anakinra (2.5mg/ kg/day, 78.9 PYs)<br>1 cellulitis, 3 fungal skin infections, 16 ear infections, 11 gastroenteritis, 5 pneumonia, 10 sinusitis, 62 upper respiratory tract infections, 4 urinary tract infections, 3 streptococcal pharyngitis.				
**Malignancy**	No malignancies were observed.				

**ILD**	Not reported.				
**Drug reaction**	Injection site reactions occurred frequently. 1 angioedmema (low-dose group), 1 pruritis (high dose group).				
**SAE**	Six serious adverse events were thought to be possibly related to the study drug. These included 2 wound infections, an episode of macrophage activation syndrome (MAS), post traumatic hypopyon, vertigo,and gastroenteritis. Anakinra was not discontinued in any patient or during infections.The patient who developed MAS had 2 episodes of MAS before starting anakinra and 2 episodes while receiving anakinra.				
**Discontinuation**	No patient discontinued the study drug.				
**Death**	Not reported.				
**Additional notes**	Nil				
**Risk of bias (<6 high, 6-11 moderate, >11 low risk of bias)**					
**Aim**	2	**Consecutive**	1	**Prospective**	2
**Endpoints**	1	**Assessment**	0	**Follow-up**	2
**Loss to follow-up**	0	**Calculation**	0	**Total**	8 (moderate)
ANA anakinra, CAPS cryopyrin-associated periodic syndromes, ILD interstitial lung disease, PY patient-year, SAE serious adverse event, SD standard deviation					

**Study**	Sota 2018				
**Primary reference**	Sota J, Insalaco A, Cimaz R, Alessio M, Cattalini M, Gallizzi R, et al. Drug Retention Rate and Predictive Factors of Drug Survival for Interleukin-1 Inhibitors in Systemic Juvenile Idiopathic Arthritis. Front Pharmacol. 2018;9:1526.				
**Associated references**	Sota 2019		**Study identifier**	364-16OCT2013	
**Study characteristics**					
**Date of study**	Jan 2008 to Jul 2016	**Design**	Retrospective cohort study	**Condition**	SJIA
**Medication**	ANA (61)<br>CAN (25)<br>(9 had both)	**Number of patients (IL-1 treated)**	77 (77)	**Age**	Mean 12.7 years (SD 6.7)
**Follow-up**	Mean 22.7 months (SD 19.5)	**Country**	Italy	**Sex**	34 M / 43 F
**Author’s conclusions**	Our findings suggest an excellent overall DRR for both ANA and CAN that might be further augmented by paying attention to AEs and employing these agents as first-line biologics in an early disease phase.				
**Outcomes**					

**Infection**	Not reported.
**Malignancy**	Not reported.
**ILD**	Not reported.
**Drug reaction**	7 injection site reactions.
**SAE**	No SAEs were recorded.
**Discontinuation**	AEs were responsible for 10 cases of treatment discontinuation.
**Death**	Not reported.
**Additional notes**	Nil.

**Risk of bias (<6 high, 6-11 moderate, >11 low risk of bias)**					
**Aim**	1	**Consecutive**	1	**Prospective**	0
**Endpoints**	1	**Assessment**	0	**Follow-up**	0
**Loss to follow-up**	0	**Calculation**	0	**Total**	3 (high)

ANA anakinra, AE adverse event, CAN canakinumab, ILD interstitial lung disease, SAE serious adverse event, SD standard deviation, SJIA systemic onset juvenile idiopathic arthritis

**Study**	Sota 2019		
**Primary reference**	Sota J, Rigante D, Ruscitti P, Insalaco A, Sfriso P, de Vita S, et al. Anakinra Drug Retention Rate and Predictive Factors of Long-Term Response in Systemic Juvenile Idiopathic Arthritis and Adult Onset Still Disease. Front Pharmacol. 2019;10:918.		
**Associated references**	Sota 2018	**Study identifier**	364-16OCT2013

**Study characteristics**					
**Date of study**	Jan 2008 to Jul 2016	**Design**	Retrospective cohort study	**Condition**	SJIA (61)<br>AOSD (76)
**Medication**	ANA 137	**Number of patients (IL-1 treated)**	137 (137)	**Age**	SJIA mean 13.0 years (SD 7.0)
**Follow-up**	Median 18 months (IQR 27)*	**Country**	Italy	**Sex**	M 30 / F 31

**Author's conclusions**	Our findings display an overall excellent DRR of ANA on the long run for both sJIA and AOSD, that may be further optimized by closely monitoring patient's safety issues and employing this IL-1 inhibitor as a first-line biologic as early as possible. Moreover, ANA allowed a significant drug-sparing effect and showed an overall good safety profile.				
**Outcomes**					
**Infection**	No infection in SJIA group.				
**Malignancy**	Not reported.				
**ILD**	Not reported.				
**Drug reaction**	7/61 injection site reaction, 2/61 generalised skin rash.				
**SAE**	No SAE in the SJIA group.				
**Discontinuation**	Not reported (AEs caused treatment discontinuation in 29 cases but not disaggregated for SJIA group).				
**Death**	No deaths in the SJIA group.				
**Additional notes**	* median time on treatment for whole cohort (not disaggregated for SJIA group but retention rate without any significant differences between SJIA and AOSD patients)				
**Risk of bias (<6 high, 6-11 moderate, >11 low risk of bias)**					
**Aim**	1	**Consecutive**	1	**Prospective**	0
**Endpoints**	1	**Assessment**	0	**Follow-up**	0
**Loss to follow-up**	0	**Calculation**	0	**Total**	3 (high)
ANA anakinra, AOSD adult onset Still's disease, ILD interstitial lung disease, IQR interquartile range, SAE serious adverse event, SD standard deviation, SJIA systemic onset juvenile idiopathic arthritis					

**Study**	Tanatar 2023				
**Primary reference**	Tanatar A, Aktay Ayaz N. Biologic Therapies in Juvenile Idiopathic Arthritis. Bakirkoy Tip Dergisi / Medical Journal of Bakirkoy. 2023;19(3):269-75.				
**Associated references**	Nil		**Study identifier**	Nil	
**Study characteristics**					
**Date of study**	January 2010-December 2021.	**Design**	Retrospective cohort study	**Condition**	JIA

**Medication**	ANA (7)<br>CAN (5)	**Number of patients (IL-1 treated)**	237 (12)	**Age**	14.4 years (IQR 10.7-18) (for all biologics)
**Follow-up**	Median 3.9 years (IQR 2-6.3)	**Country**	Türkiye	**Sex**	122 M, 115 F
**Author's conclusions**	Biological agents are effective and safe but adverse events should not be underestimated and benefit-risk balance should be carefully interpreted.				
**Outcomes**					
**Infection**	0 Upper respiratory tract infections, 0 chickenpox, 1 cytomegalovirus, 0 COVID-19, 0 scabies, 0 verruca vulgaris, 0 parotitis, 0 pneumonia, 0 preseptal cellulitis, and 0 lung tuberculosis.				
**Malignancy**	0.				
**ILD**	Not reported.				
**Drug reaction**	3 had injection site reactions.				
**SAE**	7 patients. 1 hepatic failure, 2 erythematous skin rashes, others unspecified.				
**Discontinuation**	Anakinra was discontinued in one patient because of the development of diffuse hypersensitivity reaction and angioedema after the first dose. Another patient presented with moderate to severe hepatic failure after the 12th dose of anakinra and recovered spontaneously after discontinuation of treatment.				
**Death**	0.				
**Additional notes**	AEs were significantly higher in patients on anti-IL-1 therapy compared to other groups.				
**Risk of bias (<6 high, 6-11 moderate, >11 low risk of bias)**					
**Aim**	2	**Consecutive**	2	**Prospective**	0
**Endpoints**	1	**Assessment**	0	**Follow-up**	1
**Loss to follow-up**	0	**Calculation**	0	**Total**	6 (moderate)
ANA anakinra, AE adverse events, CAN canakinumab, ILD interstitial lung disease, JIA juvenile idiopathic arthritis, SAE serious adverse event					

**Study**	Thiele 2021
**Primary reference**	Thiele F, Klein A, Windschall D, Hospach A, Foeldvari I, Minden K, et al. Comparative risk of infections among real-world users of biologics for juvenile idiopathic arthritis: data from the German BIKER registry. Rheumatol Int. 2021;41(4):751-62.

**Associated references**	Atemnkeng Ntam 2021<br>Horneff 2017<br>Klein 2020		**Study identifier**	Nil	
**Study characteristics**					
**Date of study**	Jan 2001 to Mar 2020	**Design**	Registry	**Condition**	JIA
**Medication**	ANA (63)<br>CAN (61)<br>(19 had both)	**Number of patients (IL-1 treated)**	3258 (105)	**Age**	9.1 years (IQR 4.8 to 13.3)
**Follow-up**	Mean 1.98 years (total 207.5 PYs)	**Country**	Germany	**Sex**	M 64 / F 41
**Author's conclusions**	The safety profiles of actually approved biologics are highly acceptable. However, this analysis shows that both common infections and infections requiring hospitalization are more frequent in JIA patients treated with IL-1i or IL-6i.				
**Outcomes**					
**Infection**	Incident infections 36/105 (34.3%). 9 (8.6%) SAE infections, 0 herpes zoster, 2 (1.9%) pneumonia, 1 (1.0%) varicella.				
**Malignancy**	Not reported.				
**ILD**	Not reported.				
**Drug reaction**	Not reported.				
**SAE**	9 (8.6%) SAE infections.				
**Discontinuation**	Not reported.				
**Death**	Not reported.				
**Additional notes**	Nil.				
**Risk of bias (<6 high, 6-11 moderate, >11 low risk of bias)**					
**Aim**	2	**Consecutive**	1	**Prospective**	1
**Endpoints**	2	**Assessment**	0	**Follow-up**	0
**Loss to follow-up**	0	**Calculation**	0	**Total**	6 (moderate)
ANA anakinra, CAN canakinumab, ILD interstitial lung disease, JIA juvenile idiopathic arthritis, SAE serious adverse event					

Study	Woerner 2015				
**Primary reference**	Woerner A, Uettwiller F, Melki I, Mouy R, Wouters C, Bader-Meunier B, et al. Biological treatment in systemic juvenile idiopathic arthritis: achievement of inactive disease or clinical remission on a first, second or third biological agent. RMD Open. 2015;1(1):e000036.				
**Associated references**	Nil		**Study identifier**	Nil	
**Study characteristics**					
**Date of study**	Jan 2005 to Jun 2012	**Design**	Registry	**Condition**	SJIA
**Medication**	ANA (58)<br>CAN (30)<br>(18 had both)	**Number of patients (IL-1 treated)**	77 (70)	**Age**	ANA median 3.6 years (IQR 2.3-6.8)*<br>CAN median 6.0 years (IQR 5.0-8.4)*
**Follow-up**	ANA mean 2.2 years (127.7 PYs)<br>CAN mean 1.6 years (48.7 PYs)	**Country**	France	**Sex**	ANA M 24 / F 27*<br>CAN M 6 / 4*
**Author's conclusions**	In this series of patients with SJIA, interleukin-1 inhibitors were associated with a higher proportion of ID than tumour necrosis factor inhibitors when used as first BA. Switching allowed some patients to achieve ID when treated with canakinumab or tocilizumab. CR was eventually achieved in more than half of the patients.				
**Outcomes**					
**Infection**	Not reported.				
**Malignancy**	No case of cancer was recorded.				
**ILD**	Not reported.				
**Drug reaction**	No allergic reaction, infusion reaction or other adverse event in immediate relation to a drug administration recorded for ANA or CAN.				
**SAE**	ANA 2 varicella infection, 1 pneumonia, 1 macrophage activation syndrome, 1 salmonella infection, 1 mycoplasma pneumonia<br><br>CAN 2 cytomegalovirus infection, 1 lymphadenitis, 1 eczema, 1 vulvitis, 1 lyme disease, 1 gastroenteritis, 1 macrophage activation syndrome, 1 varicella infection				
**Discontinuation**	Not reported.				
**Death**	No case of death was recorded.				
**Additional notes**	* data reported for patient starting IL-1 treatment as first biologic (51/58 ANA and 10/30 CAN treated patients had these drugs as their first biologic)				
**Risk of bias (<6 high, 6-11 moderate, >11 low risk of bias)**					
**Aim**	2	**Consecutive**	2	**Prospective**	1

**Endpoints**	1	**Assessment**	0	**Follow-up**	1
**Loss to follow-up**	0	**Calculation**	0	**Total**	7 (moderate)
ANA anakinra, CAN canakinumab, ILD interstitial lung disease, IQR interquartile range, PY patient-year, SAE serious adverse event, SJIA systemic onset juvenile idiopathic arthritis					

**Study**	Yazilitas 2018				
**Primary reference**	Yazilitas F, Aydog O, Ozlu SG, Cakici EK, Gungor T, Eroglu FK, et al. Canakinumab treatment in children with familial Mediterranean fever: report from a single center. Rheumatol Int. 2018;38(5):879-85.				
**Associated references**	Nil		**Study identifier**	Nil	
**Study characteristics**					
**Date of study**	Jan 2012- Jan 2017	**Design**	Retrospective cohort study	**Condition**	FMF
**Medication**	CAN (11)	**Number of patients (IL-1 treated)**	11 (11)	**Age**	6-17 years (median 14 years)
**Follow-up**	Median duration of canakinumab use was 21 months (range: 5-49 months)	**Country**	Türkiye	**Sex**	6 M/ 5 F
**Author's conclusions**	The authors suggest that canakinumab may be a safe and effective therapy in patients who are resistant to colchicine and even in patients with amyloidosis. They also suggest canakinumab might be a safe option for patients with uveitis.				
**Outcomes**					
**Infection**	1 patient developed pneumonia following an upper respiratory tract infection, 1 died due to sepsis 1 year post CAN cessation.				
**Malignancy**	Not reported.				
**ILD**	Not reported.				
**Drug reaction**	Not reported.				
**SAE**	Not reported.				
**Discontinuation**	1 patient discontinued canakinumab due to uveitis reactivation.				
**Death**	1 due to sepsis related with mixed fungal and staphylococcal peritonitis (1 year after CAN cessation).				
**Additional notes**	No MAS or anaphylaxis.				
**Risk of bias (<6 high, 6-11 moderate, >11 low risk of bias)**					

**Aim**	2	**Consecutive**	1	**Prospective**	0
**Endpoints**	1	**Assessment**	0	**Follow-up**	0
**Loss to follow-up**	0	**Calculation**	0	**Total**	4 (high)
CAN canakinumab, FMF familial mediterranean fever, ILD interstitial lung disease, MAS macrophage activation syndrome, SAE serious adverse event					

**Study**	Yokota 2017				
**Primary reference**	Yokota S, Imagawa T, Nishikomori R, Takada H, Abrams K, Lheritier K, et al. Long-term safety and efficacy of canakinumab in cryopyrin-associated periodic syndrome: results from an open-label, phase III pivotal study in Japanese patients. Clin Exp Rheumatol. 2017;35 Suppl 108(6):19-26.				
**Associated references**	Nil		**Study identifier**	Nil	
**Study characteristics**					
**Date of study**	Oct 2009-Feb 2012	**Design**	Open label prospective study	**Condition**	CAPS
**Medication**	CAN (19)	**Number of patients (IL-1 treated)**	19 (19)	**Age**	2-48 years (median 14 years)
**Follow-up**	Median 109 weeks	**Country**	Japan	**Sex**	12 M / 7 F
**Author's conclusions**	Canakinumab treatment every 8 weeks at dose levels from 2-8 mg/kg represents a successful strategy to induce rapid and complete response and maintain long-term disease control in Japanese patients with CAPS. The safety profile of canakinumab was consistent with that observed from previous studies.				
**Outcomes**					
**Infection**	All patients experienced at least one adverse event (AE), the most common being infections (100%).				
**Malignancy**	Not reported.				
**ILD**	Not reported.				
**Drug reaction**	Severe diffuse vasculitis was reported in one NOMID patient from Day 4 onwards, however, the investigator did not suspect it to be canakinumab-related. Mild injection-site reaction was reported in one NOMID patient (at Day 143), which resolved without intervention.				
**SAE**	5 patients (26.3%) reported serious AEs. One 4 year-old female with MWS had multiple infections over the study (Epstein–Barr virus and suspected parvovirus later with mumps meningitis and then herpes zoster; on concomitant methotrexate and prednisolone); pneumonia (n=1); sinoatrial block and headache (n=1); asthma (n=1); and appendicitis (n=1).				
**Discontinuation**	One patient discontinued the study early due to withdrawal of consent. No discontinuation due to AEs. In one patient, the dose was delayed 5 days because of parotitis.				

**Death**	None.				
**Additional notes**	The frequency and pattern of AEs reported in patients whose dose was increased or whose dosing interval decreased was similar to those without any dose adjustment.				
**Risk of bias (<6 high, 6-11 moderate, >11 low risk of bias)**					
**Aim**	2	**Consecutive**	1	**Prospective**	1
**Endpoints**	2	**Assessment**	0	**Follow-up**	0
**Loss to follow-up**	2	**Calculation**	0	**Total**	8 (moderate)
CAN canakinumab, CAPS cryopyrin-associated periodic syndrome, ILD interstitial lung disease, NOMID neonatal-onset multisystem inflammatory disease, SAE serious adverse event					

**Study**	Yucel 2021				
**Primary reference**	Yucel BB, Aydog O, Nalcacioglu H, Yilmaz A. Effectiveness of Canakinumab Treatment in Colchicine Resistant Familial Mediterranean Fever Cases. Front Pediatr. 2021;9:710501.				
**Associated references**	Nil		**Study identifier**	Nil	
**Study characteristics**					
**Date of study**	Aug 2016- Aug 2020	**Design**	Retrospective cohort study	**Condition**	Colchicine-resistant FMF
**Medication**	CAN (65)	**Number of patients (IL-1 treated)**	65 (65)	**Age**	Mean age at diagnosis 5.6 years (SD 3.9)
**Follow-up**	Mean duration of canakinumab use was 31.4 months (SD 10.6)	**Country**	Türkiye	**Sex**	29 M/ 36 F
**Author's conclusions**	Our study showed that canakinumab treatment was highly effective, well-tolerated in pediatric FMF patients, and controlled extension of the canakinumab dose interval was safe.				
**Outcomes**					
**Infection**	Cervical lymphadenitis developed in one patient.				
**Malignancy**	Not reported.				
**ILD**	Not reported.				
**Drug reaction**	Local reactions developed in two patients.				

**SAE**	No severe adverse effects requiring discontinuation of canakinumab treatment were observed.				
**Discontinuation**	Canakinumab treatment was discontinued in three patients with complete remission and one patient with drug resistance.				
**Death**	Not reported.				
**Additional notes**	Nil.				
**Risk of bias (<6 high, 6-11 moderate, >11 low risk of bias)**					
**Aim**	1	**Consecutive**	1	**Prospective**	0
**Endpoints**	2	**Assessment**	0	**Follow-up**	1
**Loss to follow-up**	0	**Calculation**	0	**Total**	5 (high)
CAN canakinumab, FMF familial mediterranean fever, ILD interstitial lung disease, SAE serious adverse event, SD standard deviation					

**Study**	Zhang 2025				
**Primary reference**	Zhang W, Chen Y, Yao Z, Ouyang M, Sun M, Zou S. Post-Marketing Pharmacovigilance of Canakinumab from the FDA Adverse Event Reporting System (FAERS). Pharmaceuticals (Basel). 2025;18(1).				
**Associated references**	Nil	**Study identifier**	Nil		
**Study characteristics**					
**Date of study**	2009 to 2024	**Design**	Post-marketing pharmacovigilance reporting system	**Condition**	Still's disease 1997 (7.0%), CAPS 1289 (4.5%), JIA 1134 (4.0%), cardiovascular prophylaxis 741 (2.6%), pyrexia 680 (2.4%)
**Medication**	CAN (not reported)	**Number of patients**	Not reported	**Age**	Median 18 years (IQR 9 to 48)
**Follow-up**	Not reported	**Country**	United States, Canada, Japan, Germany, United Kingdom	**Sex**	M 10,693 / F 16,207 / not reported 1,596
**Author's conclusions**	We found that most of the suspicious signals were associated with infections. More attention should be paid to serious infections, particularly in males, individuals aged ≥60 years, or those weighing >100 kg, who demonstrated the highest risk of serious infections.				
**Outcomes**					
**Infection**	Pneumonia (333 cases), COVID 19 (257 cases), nasopharyngitis (231 cases), influenza (187 cases), rhinorrhea (123 cases), URTI (77 cases), streptococcal pharyngitis (50 cases), tonsillitis (38 cases), rhinitis (17 cases), cellulitis (93 cases), subcutaneous abscess (18 cases), erysipelas (18 cases), impetigo (14 cases), varicella (12 cases), viral infection (67), ear infection (66), conjunctivitis (28),				

	abscess (26), streptococcal infection (22), otitis media (30), EBV (18), meningitis (17), IM (11), rhinovirus (10).
**Malignancy**	Not reported.
**ILD**	Not reported.
**Drug reaction**	Not reported.
**SAE**	6,945 (24.4%).
**Discontinuation**	Not reported.
**Death**	1,604 (5.6%).
**Additional notes**	Hospitalization 8698 (30.5%), disability 432 (1.5%), threat to life 242 (0.9%).

**Risk of bias (<6 high, 6-11 moderate, >11 low risk of bias)**					
**Aim**	2	**Consecutive**	0	**Prospective**	1
**Endpoints**	2	**Assessment**	0	**Follow-up**	0
**Loss to follow-up**	0	**Calculation**	0	**Total**	5 (high)

CAN canakinumab, EBV Epstein Barr Virus, ILD interstitial lung disease, IM infectious mononucleosis, IQR interquartile range, JIA juvenile idiopathic arthritis, SAE serious adverse event

**Study**	Zhu 2024				
**Primary reference**	Zhu X, Fan J, Huang Y, Xu Y, Yang Z, Weng R, et al. Effectiveness and safety of canakinumab in cryopyrin-associated periodic syndrome: a retrospective study in China. Pediatr Rheumatol Online J. 2024;22(1):87.				
**Associated references**	Nil		**Study identifier**	Nil	
**Study characteristics**					
**Date of study**	Mar 2021 to Feb 2024	**Design**	Retrospective cohort study	**Condition**	CAPS
**Medication**	CAN (10)	**Number of patients (IL-1 treated)**	10 (10)	**Age**	Median age at onset was 2.5 days and the median age at diagnosis was 9.5 months
**Follow-up**	Median 22.5 months (range 8.5-27.5)	**Country**	China	**Sex**	4 M / 6 F

**Author's conclusions**	Canakinumab may be effective and tolerable for Chinese CAPS patients, helping to reduce the dosage of corticosteroids. However, additional trials on large samples are required to further evaluate its efficacy and safety in China.				
**Outcomes**					
**Infection**	Respiratory infection in 4 patients.				
**Malignancy**	Not reported.				
**ILD**	Not reported.				
**Drug reaction**	Three patients experienced occasional white fibrous papular rash at the first two doses of canakinumab, which disappeared in the later treatment. One patient had dry skin.				
**SAE**	None .				
**Discontinuation**	No patients discontinued canakinumab treatment due to adverse events..				
**Death**	None .				
**Additional notes**	Nil.				
**Risk of bias (<6 high, 6-11 moderate, >11 low risk of bias)**					
**Aim**	1	**Consecutive**	1	**Prospective**	1
**Endpoints**	1	**Assessment**	0	**Follow-up**	0
**Loss to follow-up**	1	**Calculation**	0	**Total**	5 (high)
CAN canakinumab, CAPS cryopyrin-associated periodic syndrome, ILD interstitial lung disease, SAE serious adverse event					
